# Ferroelectric Nematic Liquid Crystals Showing High Birefringence

**DOI:** 10.1002/advs.202414317

**Published:** 2025-01-13

**Authors:** Yaohao Song, Xiang Huang, Xinxin Zhang, Minghui Deng, Satoshi Aya, Mingjun Huang

**Affiliations:** ^1^ South China Advanced Institute for Soft Matter Science and Technology School of Emergent Soft Matter South China University of Technology Guangzhou 510640 China; ^2^ Guangdong Provincial Key Laboratory of Functional and Intelligent Hybrid Materials and Devices Guangdong Basic Research Center of Excellence for Energy and Information Polymer Materials South China University of Technology Guangzhou 510640 China

**Keywords:** ferroelectric nematic, high birefringence, liquid crystal

## Abstract

High birefringence nematic liquid crystals are particularly demanded for adaptive optics applications in the infrared spectrum because it enable a thinner cell gap for achieving fast response time and improved diffraction efficiency. The emerging ferroelectric nematic liquid crystals have attracted widespread interest in soft matter due to their unique combination of ferroelectricity and fluidity. However, the birefringence, which is one of the most important optical parameters in electro‐optic devices, is not large enough (<0.25) in most ferroelectric nematic materials. Here, a polar liquid crystal molecule library containing more than 60 molecules with a highly rigid and fluorinated nature is developed. The introduction of triple bonds constructs a long π‐electron conjugated mesogen skeleton, significantly improving the birefringence of polar liquid crystal phases. The birefringence and dispersion properties are systematically studied, demonstrating a strong dependence on chemical structures and the type of polar phases. Furthermore, through multi‐component mixing, polar liquid crystal mixtures with ultra‐wide temperature range and excellent stability at or near room temperature are obtained. They possess much higher birefringence than the existing ferroelectric liquid crystal materials. The unique combination of high birefringence and fluidic ferroelectricity is expected to promote the application of polar liquid crystals in electro‐optic technologies.

## Graphical abstract

A library of >60 polar liquid crystal molecules is developed with high birefringence, by introducing long π‐conjugated mesogen structures. A systematic analysis of birefringence and dispersion across chemical structures and liquid crystal phases is constructed. Multi‐component mixtures with high birefringence, at or below room temperature stability, and broad polar phase temperature windows offer promising potential for advanced electro‐optic applications.

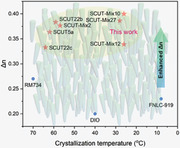

## 1 Introduction

Most modern displays for televisions, mobile phones, smartwatches, laptops, and other devices employ liquid crystals (LCs) as tunable media.^[^ ^1^ ^]^ In phase modulation, the phase retardation (δ) is defined by the equation: δ = 2π*d*Δ*n*/*λ*, where *d* is the cell thickness, Δ*n* is the LC birefringence, and *λ* is the wavelength. For typical display applications, a phase retardation of π is required and a Δ*n* value of ≈0.1 is sufficient. On the other hand, the LC materials have demonstrated widespread applications in adaptive optics in the infrared spectral range^[^ ^2^ ^]^ and the GHz or even THz ranges, such as beam steering devices,^[^ ^3^ ^]^ spatial light modulators,^[^ ^4^ ^]^ tunable waveguides,^[^ ^5^ ^]^ optical fibers,^[^ ^6^ ^]^ and filters^[^ ^7^ ^]^ among others. Especially in spatial light modulators, 2π phase modulation is necessary, along with requirements for a fast response time (<5 ms) and low operating voltage. To meet these requirements, a thinner cell combined with a larger Δ*n* is required.^[^ ^1e^ ^]^ LC molecules with high birefringence require a high degree of electronic polarizability anisotropy (α_e_), which is feasible in the mesogens consisting of a long conjugated π‐electron system.^[^ ^8^ ^]^ Efficient strategies in molecular designs include directly linking the benzene rings, introducing triple bonds as bridging bonds to form rigid aromatic mesogen cores and incorporating high electron density elements (e.g., N, S). However, due to their excessive rigidity and large mesogen length, these high‐birefringence LCs tend to crystallize at high temperatures. Moreover, they possess high viscosity and a worse response rate.

Introducing strong polarity or ferroelectricity is an important strategy to accelerate the response of LCs to the electric field, as demonstrated in the well‐known ferroelectric chiral smectic (SmC*) LCs.^[^ ^9^ ^]^ The fluid ferroelectrics, called ferroelectric nematics (N_F_), have become available recently by incorporating large dipole moments into mesogens.^[^ ^10^ ^]^ The equal distribution of two permissible director orientations, i.e., **n** and −**n**, is broken, facilitating spontaneous polarization along the long axes of the molecules.^[^ ^10d^ ^]^ The further progression from the N_F_ phase to other liquid‐matter ferroelectrics represents a remarkable journey in emerging polar soft matter, including the antiferroelectric intermediate (N_x_, also referred to N_s_, SmZ_A_, or M2 in some other papers),^[^ ^10^, ^11^ ^]^ the helielectric nematic (HN* or N_F_*),^[^ ^12^ ^]^ the ferroelectric smectic A (SmA_F_),^[^ ^13^ ^]^ and the recently reported heliconical ferroelectric nematic (N_TBF_ or ^HC^N_F_),^[^ ^14^ ^]^ polar heliconical smectic C (SmC_P_^H^)^[^ ^15^ ^]^ and the ferroelectric smectic C (SmC_F_)^[^ ^14b^ ^]^ phases. These polar LCs have attracted widespread attention in soft matter due to their extraordinary properties, including high polarization density,^[^ ^10^, ^13^, ^14^ ^]^ high dielectric constant,^[^ ^16^ ^]^ strong nonlinear optical response,^[^ ^12^, ^17^ ^]^ low driving electric field,^[^ ^10^, ^12^ ^]^ special electric field effect,^[^ ^18^ ^]^ and piezoelectricity,^[^ ^19^ ^]^ etc. Notably, Nishikawa et al. found that HN* LCs exhibit ultra‐fast electric field responses in the microsecond range.^[^ ^12b^ ^]^ Recent studies by Perera et al. indicate that HN* lenses exhibit ultra‐fast response characteristics in the tens to hundreds of microseconds range.^[^ ^20^ ^]^

**Figure 1:**
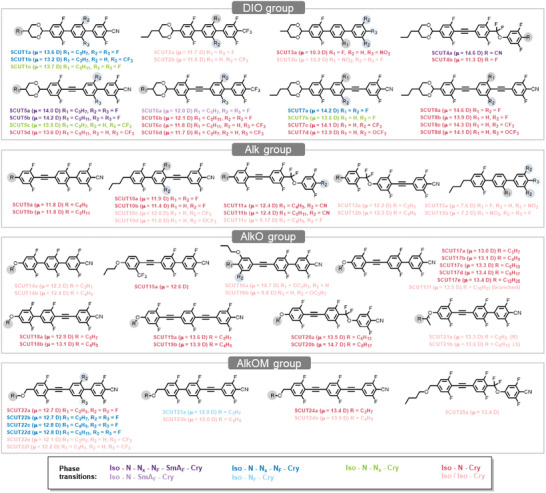
Molecular library of rigid polar LC materials. The molecular dipole moment values calculated by DFT are noted in brackets. The compounds are also marked with different colors, representing different phase transition pathways during the cooling process.

Birefringence is one of the most important physical parameters of LC materials, which directly determines the phase retardation. However, the birefringence of current N_F_ LCs remains relatively low. For example, the Δn of RM734 is 0.22–0.28 (@≈535 nm),^[^ ^12^, ^21^ ^]^ that of DIO is 0.20 (@550 nm),^[^ ^22^ ^]^ and that of FNLC‐919 is 0.22 (@535 nm).^[^ ^23^ ^]^ Other reported N_F_ LCs are mostly derived from the RM734 and DIO molecular skeletons. These molecules mainly use ester or difluoromethyl ether as bridge bonds. Herein, we develop a **SCUT** molecular family of more than 60 rigid aromatic LC molecules (**Figure** **1**). These LC molecules possess not only large dipole moments (>9 Debye, D) but also long π‐electron conjugated mesogens, in which triple bonds are extensively incorporated to construct tolane‐like structures. Furthermore, the extended highly rigid shape promotes more compact packing of LC molecules, resulting in a significant increase in the birefringence of the developed N_F_ LCs. As a result, these developed N_F_ LC materials exhibit high birefringence (≈0.4 @550 nm), good phase stability near room temperature, and an expanded phase temperature window. The significantly improved birefringence performance provides polar LCs with enhanced potential for innovations in LC electro‐optic devices.

## 2 Result and Discussion

The strategy for achieving high birefringence in polar LC molecules here is to construct a long π‐electron conjugated system with intrinsically high electronic polarizability anisotropy. Thus, this molecular library includes rigid mesogen cores such as biphenyl, terphenyl, tolane, phenyl tolane, and bitolane (Figure 1). The compounds are categorized into four groups based on the electron‐donating head groups: alkyldioxane (DIO), alkyl (Alk), alkoxy (AlkO), and alkoxymethyl (AlkOM) groups. The phase transition temperatures were determined using differential scanning calorimetry (DSC) (Figures S1–S3, Supporting Information), and the phase structures were identified from the 2D and 1D integrated X‐ray diffraction (XRD) patterns (Figures S4–S6, Supporting Information). The phase behavior of each molecule was then comprehensively evaluated by the polarized optical microscopy (POM) textures observed during the cooling process. Detailed phase transition pathways and temperatures for each compound in the molecular library are summarized in Figure 1 and Table S1 (Supporting Information). Although most molecules in the library possess large dipole moments exceeding the critical values,^[^ ^10e^ ^]^ only a few of them show stable polar LC phases (N_x_, N_F_, or SmA_F_). Increasingly detailed studies have shown that a large dipole moment alone is insufficient to generate the ferroelectric order in LC molecules. Other factors, such as the modulation of molecular surface charge density and molecular geometry should also be considered comprehensively.^[^ ^24^ ^]^

**Figure 2:**
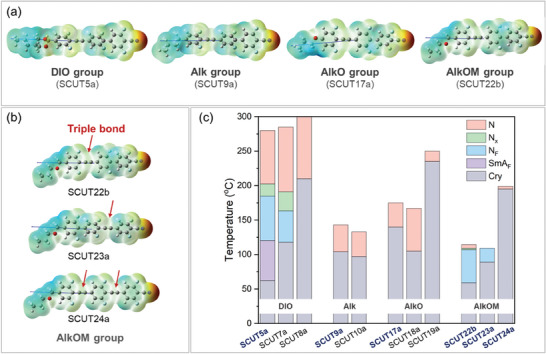
Molecular conformations and phase diagrams of representative SCUT molecules. a) Molecular conformations with different head groups (DIO, Alk, AlkO, and AlkOM). b) Molecular conformations of molecules with different triple bond positions and numbers in the AlkOM group. c) Corresponding phase diagrams of representative LC molecules.

Upon examining the phase behaviors of different groups, none of the molecules in the Alk and AlkO groups exhibit polar LC phases. Apparently, the influence of crystallization becomes more pronounced in these highly rigid and conjugated molecular systems. The rigid structure of the long conjugated π‐electron system enhances π‐π interaction between molecules, leading to a greater tendency for crystallization. Most molecules exhibit high clearing points and rapidly crystallize before transforming into the N_F_ phase upon cooling. Nevertheless, many polar LCs are identified in the DIO and AlkOM groups. **Figure** **2**a illustrates the molecular conformations of SCUT5a, SCUT9a, SCUT17a, and SCUT22b for comparison. They have similar backbone structures of mesogens but different head groups. The 1,3‐dioxane group in SCUT5a is a widely used nonplanar cyclic structure that significantly reduces π‐π interaction among rigid mesogens, suppressing the crystallization capability while maintaining high shape anisotropy conducive to polar LC phase formation. Replacing the rigid dioxane electron‐donating groups with more flexible alkyl or alkoxy chains effectively lowers the phase temperatures, albeit at the expense of shape anisotropy that promotes LC phase formation (Table S1, Supporting Information). Among the three linear aliphatic head groups (Alk, AlkO, and AlkOM), the most distorted conformation is observed in the AlkOM group of SCUT22b (Figure 2a), endowing the lowest melting and crystallization temperature (Figure 2c). In comparison, with the Alk group primarily consists of *trans* conformation, the additional oxygen atom at the *β*‐position of the aromatic ring introduces a higher fraction of *gauche* conformations in the AlkOM group, effectively disrupting the regularity of the aliphatic head group and suppressing crystallization. Various polar LC phases emerge in molecules of the AlkOM group when crystallization is delayed upon cooling. Surprisingly, moving the oxygen one atom position away from the aromatic ring causes dramatic conformation change and phase behavior difference between AlkO and AlkOM groups. This strategy of incorporating AlkOM groups may serve as a general approach for stabilizing polar LC phases at low temperatures.

To develop polar LCs with high birefringence, special attention is given to molecules containing the tolane group. It has been observed that the position and number of triple bonds significantly influence the phase behaviors. Figure 2b shows the DFT‐calculated molecular conformations of SCUT22b, SCUT23a, and SCUT24a, all of which belong to the AlkOM group. SCUT22b exhibits a wide temperature window (≈50 °C) for the N_F_ phase. Shifting the triple bond toward the terminal nitrile group in SCUT23a results in a significantly increased crystallization temperature and narrows the N_F_ phase window. Additionally, this shift results in high‐temperature thermal instability of the samples (Figure S7, Supporting Information). Our previous work has demonstrated that this thermal instability also occurs when the bridging bond adjacent to a strong electron‐withdrawing group is an ester group.^[^ ^25^ ^]^ The introduction of additional triple bonds is anticipated to yield a further increase in birefringence. However, due to excessive shape anisotropy and rigidity, crystallization predominates in SCUT24a, with its crystallization temperature exceeding 200 °C. The trend of the triple bond effect is consistent across all molecular groups (Table S1, Supporting Information). In this work, we fail to obtain a polar N_F_ LC phase in any of the molecules with two triple bonds.

In the chemical structure modification of polar LCs, the type and position of other functional groups are also important. The twisted conformation of the ─CF_2_O─ bridging bond in the UUQU‐4N molecule and other molecules with similar backbones significantly reduces the phase transition temperature.^[^ ^26^ ^]^ The introduction of the ─CF_2_O─ bridging bond in SCUT4a can notably lower the clear point of the molecule by ≈100 °C (compared to SCUT5a) while maintaining the same phase transition pathway. Replacing the electron‐withdrawing terminal group with ─CF_3_ in SCUT6a may be a good modification approach. Although there is a loss in dipole moment compared to SCUT5a which leads to an increase in crystallization temperature, it still demonstrates a stable SmA_F_ phase. Notably, due to the disappearance of the N_F_ phase with a high density of disclination lines, the SmA_F_ phase of SCUT6a inherits the flat texture of the N phase, resulting in a large area of defect‐free, uniform SmA_F_ textures. Extending the flexible carbon chain is a very effective approach for lowering the temperature of traditional non‐polar N LCs. However, in polar LCs, excessively long flexible carbon chains are not preferred, as this leads to a decrease in molecular dipole density, which severely impacts the stability of the polar LC phase.^[^ ^10f^ ^]^ Based on the results from the continuous modulation of flexible carbon chain lengths in SCUT22a‐SCUT22d, the optimal carbon chain length for polar LC molecules appears to be ≈3–4 carbon atoms.

**Figure 3:**
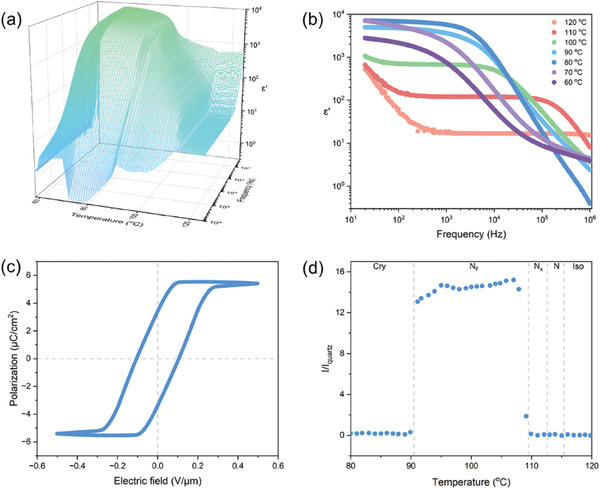
The measurements of ferroelectricity and polarity of SCUT22b molecule. a) Temperature and frequency dependence of the dielectric constant. b) Frequency dependence of dielectric constant at selected temperatures in a). c) The P–E hysteresis loop measured in the N_F_ phase (80 °C). Cell thickness: 20 µm. *V*_p_ = 10 V, *f* = 200 Hz. d) Temperature dependence of SHG signal intensity measured in syn‐parallel alignment cell. Cell thickness: 5 µm.

The characteristic POM textures of molecules exhibiting polar LC phases are shown in Figures S8–S18 (Supporting Information). Taking SCUT22b as an example, a banded texture with line domain wall defects was observed at 90 °C, indicating the presence of the N_F_ phase (Figure S15, Supporting Information). Additional measurements were also conducted to assess its ferroelectricity and strong polarity (**Figure** **3**). Analysis of the temperature and frequency dependence of the dielectric constant (ɛ″), the ɛ″ of SCUT22b is low (ɛ' < 20 @1 kHz) in the non‐polar N phase and increases to ≈100 upon transitioning into the antiferroelectric N_x_ phase (Figure 3a,b). As the temperature approaches the N_F_ phase transition, ɛ' increases rapidly to levels of 10^3^–10^4^ @1 kHz, and the response frequency also shifts toward lower frequency as the temperature continues to decrease. Subsequently, we conducted polarization switching tests, resulting in a parallelogram‐shaped P‐E loop. The measured polarization density reaches 5.4 µC cm^−2^ (@200 Hz, 80 °C), confirming the excellent ferroelectric property of SCUT22b. SCUT22b exhibits a strong second harmonic generation (SHG) response in the N_F_ phase, ≈15 times greater than that of fused silica. All other ferroelectric LC molecules also demonstrate a strong SHG response in the polar phases.

**Figure 4:**
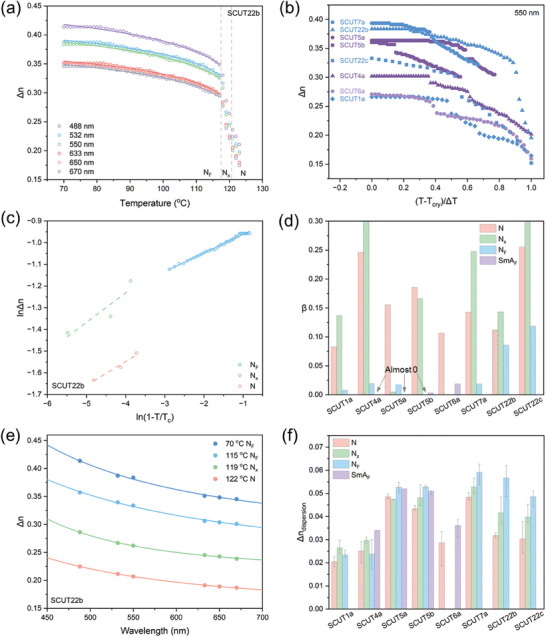
Birefringence and dispersion properties of polar LC molecules. a) Temperature dependencies of birefringence in SCUT22b at different wavelengths (488, 532, 550, 633, 650, and 670nm) upon cooling. The line curves are fitted based on Equation (1). Syn‐parallel alignment EHC LC cell; cell thickness: 1.9 µm. b) The temperature dependence of birefringence for various polar LC molecules. The horizontal axis is (*T*–*T*_cry_)/Δ*T*, where *T*_cry_ is the crystallization temperature and Δ*T* (Δ*T* = *T*_c_ – *T*_cry_, *T*_c_ is the clearing point) is the temperature window of the whole LC phase. Wavelength: 550 nm. c) This linear fitting of the temperature dependence of birefringence in SCUT22b. N, N_x_, and N_F_ phases are fitted separately. Wavelength: 550nm. d) The parameter β obtained by linear fitting of temperature‐dependent birefringence based on Equation (2). The shaded data columns indicate that a narrow temperature window may bring certain errors to the data. Wavelength: 550 nm. e) The dispersion of birefringence in different LC phases of SCUT22b. The line curves are fitted based on Equation (3). f) Calculated birefringence dispersion Δ*n*_dispersion_ of each LC phase for various polar LC molecules based on Equation (4). Δ*n*_dispersion_ = Δ*n*_short_ – Δ*n*_long_, where Δ*n*_short_ and Δ*n*_long_ represent the calculated birefringence values at 488 and 632.8 nm from the fitted Cauchy dispersion formula, respectively.

For each molecule exhibiting the N_F_ phase, birefringence at different wavelengths was measured during cooling under POM using a Berek compensator. The results are summarized in **Figure** **4** and Figures S8d–S16d (Supporting Information). LC molecules containing rigid phenyl tolane mesogens in this library exhibit high birefringence values. Taking SCUT22b as an example, the temperature dependence of its birefringence in the visible spectrum is shown in Figure 4a. As the cooling process or phase transition proceeds, birefringence gradually increases. A Δn value of 0.39 (@550 nm) was observed at 70 °C. Additionally, a step‐like increase in birefringence is observed during the transition from the N phase to the N_F_ phase upon cooling. This step‐like increase likely arises from the distinct molecular packing arrangements between the polar N_F_ and nonpolar N phases, resulting in a higher material density in the N_F_ state.^[^ ^27^ ^]^ Figure 4b shows the temperature dependence of birefringence for polar LC molecules with various phase transition pathways. The step‐like increase in birefringence is commonly observed in molecules undergoing the N‐N_x_‐N_F_ transition (indicated in blue), as in SCUT22b. In molecules exhibiting an N‐N_x_‐N_F_‐SmA_F_ transition (indicated in deep purple), a second step‐like increase in birefringence is captured at the transition of N_F_‐SmA_F_, as in SCUT4a (Figure S10d, Supporting Information) and SCUT5b (Figure S12d, Supporting Information). However, the absolute Δ*n* increment here is smaller than that seen in the N‐N_F_ transition. The higher birefringence observed in the SmA_F_ compared to the N_F_ phase is attributed to increased molecular packing density and enhanced orientational order in the SmA_F_ phase, as demonstrated in our previous work.^[^ ^13a^ ^]^ Given the progressive increase in birefringence from the N to N_F_ and SmA_F_ phases, a direct N‐SmA_F_ transition is expected to produce the largest step‐like increase in Δ*n*, as observed in SCUT6a (Figure 4b; Figure S13d, Supporting Information).

Examining the impact of chemical structure on birefringence, we find that the Δ*n* values of all other samples exceed that of SCUT1, indicating that the introduction of a triple bond indeed achieves a significant increase (Figure 4b). SCUT7a exhibits the highest Δn value, substantially exceeding that of SCUT5a, which has a similar chemical structure but with the triple bond position shifted toward the mesogen end (─CN). The position of the triple bond appears to influence both the phase behavior and the birefringence magnitude of LC molecules. Furthermore, a comparison between SCUT5a and SCUT7a shows that the ─CF_2_O─ bridging bond significantly impairs birefringence (Figure 4b). The ─CF_2_O─ linker disrupts the π‐electron conjugated system, reducing electronic polarizability, and also induces a more distorted molecular conformation, which in turn decreases orientational order and packing density in polar LC phases. Together, these two factors contribute to lower electronic polarizability anisotropy and birefringence. Additionally, replacing the ─CN end group in SCUT5a with ─CF_3_ in SCUT6a results in a significant loss of birefringence, likely due to the lower electronic polarizability of the ─CF_3_ group and its bulkier size, which may lead to a looser molecular arrangement.

Furthermore, fitting the temperature dependence of birefringence can yield additional insights. The experimental data were fitted using Equation (1) to obtain the extrapolated birefringence Δn_0_ and the exponent β.^[^ ^28^ ^]^

(1) $$\Delta n=\Delta n_{0}S=\Delta n_{0}{\left(1-\frac{T}{T_{c}}\right)}^{\beta }$$

where Δ*n*_0_ is the birefringence at temperature *T* = 0 K, *S* is the molecular order parameter, *T*_c_ is the clearing point of LC, and the exponent *β* is a material constant. Detailed statistics of the fitting parameter are shown in Table S2 (Supporting Information). Taking the logarithm of both sides of Equation (1) transforms it into Equation (2):

(2) $$ln\Delta n=ln\Delta n_{0}+lnS=ln\Delta n_{0}+\beta ln\left(1-\frac{T}{T_{c}}\right)$$

The data were segmented, logarithmized in each segment, and subsequently fitted linearly. This logarithmic transformation followed by linear fitting facilitates a more direct analysis of the *β* parameter, which is inversely related to the order parameter of each LC phase. The parameter *β* corresponds to the slope of the linear fit, as shown in Figures 4c and S19 (Supporting Information). The calculated *β* values at 550 nm for various polar LC molecules are shown in Figure 4d. It should be noted that the narrow temperature window of some N_x_ phases introduces fitting errors, indicated by the shaded columns. As shown in Figure 4d, the β values are relatively high for nonpolar N and antiferroelectric N_x_ phases. Conversely, *β* values significantly decrease upon transition to the polar LC phase (i.e., N_F_ and SmA_F_). In the N_F_ and SmA_F_ phases, lower *β* values correspond to a diminished rate of birefringence variation, indicating reduced temperature sensitivity in the birefringence of polar LC phases. Notably, *β* values for SCUT22b and SCUT22c (AlkOM group) in the N_F_ phase remain relatively high compared to those of other molecules in the DIO group such as SCUT7a. This may arise from the highly twisted conformations in AlkOM group molecules, resulting in a comparatively large free volume. As the free volume decreases rapidly upon cooling, the birefringence maintains a high growth rate, even in the N_F_ phase. This suggests that AlkOM group molecules hold significant potential for enhanced birefringence at lower temperatures.

Dispersion is intrinsic to birefringent materials, particularly those with high birefringence. Figure 4e illustrates the wavelength‐dependent birefringence of SCUT22b across various LC phases, with similar data for other molecules presented in Figures S8e–S16e (Supporting Information). The data are fitted using the Cauchy dispersion formula^[^ ^29^ ^]^ as follows:

(3) $$\Delta n\left(\lambda \right)=A+\frac{10^{4}\cdot B}{\lambda^{2}}+\frac{10^{9}\cdot C}{\lambda^{4}}$$

In the formula, *A*, *B*, and *C* represent the Cauchy coefficients: *A* is a dimensionless parameter, indicating that as λ → ∞, Δ*n*(λ) → *A*; *B* (nm^2^) and *C* (nm^4^) modulate the curvature and amplitude of birefringence of medium wavelengths in visible light and shorter wavelengths in the UV range, respectively. Figures 4b and S8e–S16e (Supporting Information) illustrate the wavelength‐dependent trend in birefringence, allowing extrapolation of birefringence behavior in the ultraviolet and infrared regions using the fitted parameters. To assess dispersion characteristics across different molecules and LC phases, the most direct approach involves comparing birefringence differences at two key wavelengths. Typically, blue light (488 nm) and red light (632.8 nm) are selected to represent short and long wavelengths, respectively. Birefringence dispersion (Δ*n*_dispersion_) is quantified by calculating the birefringence difference between these wavelengths, as shown in Equation (4):

(4) $$\Delta n_{dispersion}=\Delta n_{short}-\Delta n_{long}$$

where Δ*n*_short_ and Δ*n*_long_ represent the birefringence values at 488 and 632.8 nm, respectively. The parameters in Equation (2) were obtained by fitting the experimental data, and the specific wavelength values (i.e., 488 and 632.8 nm) were then substituted to determine the corresponding birefringence values. The Δ*n*_dispersion_ for each LC phase of these samples is shown in Figure 4f and Table S3 (Supporting Information). Despite a few exceptions, an evident trend in Δ*n*_dispersion_ emerges: N < N_x_ < N_F_ ≈ SmA_F_. The higher birefringence observed in polar LC phases appears to correlate with increased dispersion. This trend is generally consistent for each LC molecule examined; for example, SCUT7a exhibits both the highest Δ*n* and Δ*n*_dispersion_, while SCUT1a has the lowest values.

While integrating fluorinated tolane structures effectively enhances birefringence, their high rigidity also induces a strong tendency toward crystallization. Among the molecules exhibiting polar LC phases, SCUT22b‐22d from the AlkOM group show relatively low crystallization temperatures (≈60 °C), likely due to the distorted conformation of the head chains. Achieving a stable N_F_ phase at room temperature in the single‐component system with conjugated phenyl tolane molecular backbone remains challenging. Therefore, we systematically explored multi‐component mixtures based on the current library of rigid polar LC molecules, with particular attention given to SCUT22b‐22d (Table S4, Supporting Information). The POM textures of selected polar LC mixtures are shown in Figure S20 (Supporting Information). Through multi‐component mixing, properties such as reduced clearing points, suppressed crystallization temperature, expended temperature range of polar LC phases, and high birefringence values (0.3–0.4 @550 nm) are obtained. Some polar LC formulations are stable at room temperature or crystallize just below room temperature. Notably, SCUT‐Mix10 shows the widest N_F_ phase temperature range (>120 °C); SCUT‐Mix26 has the lowest clearing point, while the crystallization temperatures of SCUT‐Mix14, SCUT‐Mix26, SCUT‐Mix33, and SCUT‐Mix34 are below room temperature. Due to their structural similarity, these polar LC molecules exhibit good miscibility without phase separation. Taking SCUT‐Mix14 and SCUT‐Mix33 as examples, we measured the stability of materials through multiple methods. We repeated the POM image capture on the same LC cell, which was stored at room temperature (RT, 25–30 °C) for over a year (Figure S21, Supporting Information). Due to the possibility of slow relaxation of the LC materials in the LC cell, we were unable to capture the exact same area based on the POM texture features. However, we could still confirm that the LC materials did not exhibit crystallization or phase separation, demonstrating excellent stability. Additionally, we performed continuous recordings of the POM textures from the same region for ten days (Figure S22, Supporting Information). After the POM texture stabilized following prolonged relaxation, no significant changes were observed, further confirming the material's long‐term stability. Furthermore, the material was heated to a high temperature (80 °C) within the N_F_ phase temperature window at different rates, held at that temperature for 1 hour, and then cooled to RT at various rates (Figure S23, Supporting Information). The POM texture characteristics remained nearly unchanged. Upon heating the material to the Iso phase and cooling it at different rates, no significant phase separation was observed in the POM texture (Figure S24, Supporting Information), and the DSC phase transition temperatures fluctuated only within the measurement error (Figure S25, Supporting Information), demonstrating the excellent reproducibility of the material. The slight decrease in phase transition temperature observed with rapid cooling rate may be attributed to supercooling effects. Moreover, we found that a slow cooling process helps to eliminate disclination line defects within the material, leading to a more uniform and smooth texture over large areas (Figure S23, Supporting Information).

**Figure 5:**
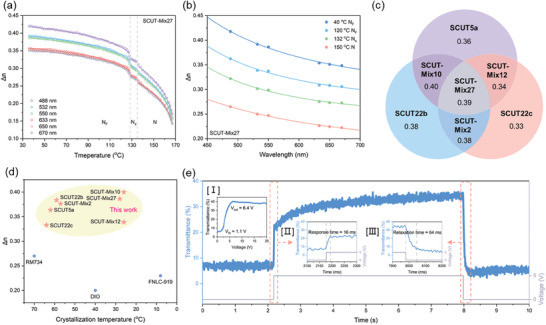
The birefringence and electro‐optic properties of polar LC mixture materials. a) Temperature dependencies of birefringence of SCUT‐Mix27 at different wavelengths (488, 532, 550, 633, 650, and 670 nm). Syn‐parallel alignment EHC LC cell; cell thickness: 2.5 µm. b) Variation of birefringence with wavelength of SCUT‐Mix27 in different LC phases. c) Schematic birefringence diagram of the representative high‐birefringence polar LC molecules and their binary/ternary mixtures. The sample names are highlighted in bold, with the numbers beneath each name representing the birefringence of the material at the wavelength of 550 nm before crystallization. d) Road maps of the birefringence versus the crystallization temperature. e) Electro‐optic response characteristics of SCUT‐Mix10 in IPS mode. The inset figures are as follows: [I] Voltage‐transmittance (V‐T) curve; [II] Enlarged turn‐on response; [III] Enlarged turn‐off relaxation. The electro‐optic response of the N_F_ phase of SCUT‐Mix10 was measured in a syn‐parallel aligned LC cell with comb‐shaped ITO electrodes, where the alignment direction was parallel to the electrodes, i.e., perpendicular to the applied electric field. Temperature: 30 °C. Electrode gaps: 1 mm. Applied voltage type: DC. Cell thickness: 6.1 µm.

Here, SCUT‐Mix27 is selected as a representative sample for comprehensive characterization. SCUT‐Mix27 is formulated by evenly mixing SCUT5a, SCUT22b, and SCUT22c in a weight ratio of 1:1:1. The phase behavior of SCUT‐Mix27 generally aligns with that of each individual component, except that it does not further transition to the SmA_F_ phase like SCUT5a. The characteristic textures of N, N_x_, and N_F_ phases for SCUT‐Mix27 are shown in Figure S26a–c (Supporting Information). Notably, the alignment in the N_F_ phase is highly uniform, exhibiting a low density of disclination line defects (Figure S26c, Supporting Information). The dynamic polarity in the N_F_ phase was probed by the dielectric measurement (Figure S26d, Supporting Information). The dielectric constant measurement reveals a gradual increase over a broad temperature range near the N_F_ phase transition. A notably high dielectric constant of over 4000 (@1 kHz) was recorded in the N_F_ phase. Figure S26e (Supporting Information) shows the parallelogram P‐E hysteresis loop, reaching a saturation polarization value of 5 µC cm^−2^. Additionally, SCUT‐Mix27 demonstrates a strong SHG signal within the N_F_ phase temperature range, ≈15–20 times that of fused silica (Figure S26f, Supporting Information). The birefringence properties of SCUT‐Mix27 were evaluated upon cooling (**Figure** **5**a). When undergoing the nonpolar‐polar phase transition (N – N_x_ – N_F_), a characteristic step increase in Δ*n* was captured, consistent with other polar LC molecules. Since the crystallization temperature is suppressed below 30 °C, Δ*n* approaches 0.4 (@550 nm) near this temperature. The dispersion data are fitted using formula (3), with the fitted curve shown in Figure 5b. The overall trend closely resembles that of the single‐component LC shown in Figure 4e.

The birefringence and dispersion contributions of individual components in polar LC mixtures are also worth exploring. We conducted binary and ternary mixing at equal molar ratios for three selected molecules from the molecular library: SCUT5a, SCUT22b, and SCUT22c. Only SCUT‐Mix2 crystalizes at ≈ 60 °C, while the other mixtures showed good stability near RT (Table S4, Supporting Information), indicating that the SCUT5a molecule plays a crucial role in enhancing the low‐temperature stability of the mixture LC materials. The temperature‐dependent birefringence during the cooling process of these four mixtures (Figure 5a; Figure S27a,c,e, Supporting Information), as well as the dispersion properties of each LC phase (Figure 5b; Figure S27b,d,f, Supporting Information), were comprehensively analyzed. The detailed statistical data for the temperature‐dependent birefringence fitting parameters are provided in Table S5 (Supporting Information). The birefringence of each molecule and their mixtures are schematically presented in Figure 5c. Among these three molecules, SCUT22b exhibits the highest birefringence, contributing the most to the overall birefringence in mixtures. The three mixtures containing SCUT22b show birefringence values higher than that of any single molecule. SCUT‐Mix2 demonstrates the lowest dispersion (Figure S28, Table S6, Supporting Information), likely due to the relatively low dispersion of the SCUT22c molecule (Figure 4f), which helps optimize the dispersion properties of the mixture. Combining the advantages of all three selected molecules, the ternary mixture SCUT‐Mix27 exhibits the best overall performance, including excellent room‐temperature stability (low crystallization temperature at 28 °C), high birefringence (Δn = 0.39@550 nm), and low dispersion (Δ*n*_dispersion_ = 0.055, N_F_).

We measured the Δ*n* values of widely studied RM734 and DIO molecules across different wavelengths during cooling (Figure S29, Supporting Information). At 550 nm, the Δ*n* of RM734 in the N_F_ phase was measured to be 0.27 (70 °C), and that of DIO was 0.20 (40 °C), both in agreement with previously reported values.^[^ ^12^, ^21^, ^22^ ^]^ Here, we compare the birefringence properties of various materials in the roadmap (Figure 5d), taking into account both birefringence magnitude and crystallization temperature. The SCUT series molecules exhibit birefringence values significantly higher than those of existing polar LC materials. Through multi‐component mixing, SCUT‐Mix27 and SCUT‐Mix10 achieve both high birefringence and a reduced crystallization temperature, close to or even falling below room temperature.

Finally, a simple electro‐optic device with in‐plane switching (IPS) mode is used to demonstrate the characteristics of high birefringence polar LC materials. The electro‐optic device employs comb‐shaped ITO electrodes to apply an in‐plane electric field, with electrode gaps of 1 mm. The device surfaces are syn‐parallel anchored and the orientation direction is perpendicular to the electric field. The SCUT‐Mix10 material, exhibiting the highest birefringence as well as good stability, was selected for demonstration of the electro‐optic device at RT (30 °C). At 0 V, the initial orientation of the LC molecules is parallel to the polarization axis of the polarizer, resulting in the minimum transmittance (≈ 6%) (Figure 5e‐I). Due to the characteristic banded texture of the N_F_ phase, the LC molecules along the disclination lines are not perfectly aligned along the orientation direction. Additionally, the chiral ground state of the N_F_ phase causes the LC molecules to prefer a twisted arrangement, making perfect parallel alignment difficult.^[^ ^30^ ^]^ These two factors combined prevent complete extinction in the initial state. The analyzer and polarizer are positioned orthogonally to each other. When an in‐plane electric field is applied and exceeds a certain threshold voltage, the LC molecules begin to orient along the direction of the electric field. The measured threshold voltage is ≈ 1.1 V, and considering the wide electrode gaps (1 mm), a small electric field of only 1.1 × 10^3^ V m^−1^ is sufficient to drive the LC molecules to tilt, which is two orders of magnitude lower than traditional non‐polar N LC materials. This is attributed to the spontaneous polarization of the LC molecules, they are able to overcome elastic forces and rotate even under very low electric fields. A saturation voltage of only ≈6.4 V (E_sat_ ≈ 6.4 × 10^3^ V m^−1^) is required to achieve the maximum transmittance (>40%) (Figure 5e–I). A voltage (20 V) far exceeding the saturation voltage was applied to the electro‐optic device, and the normalized transmittance after removing the voltage was continuously recorded. The phase modulation generated by the IPS electro‐optic device is calculated to be ≈1.4π using Malus's Law. By continuously recording the transmittance changes during the application and removal of the voltage (Figure 5e), the N_F_ LC exhibits rapid response (response time ≈16 ms, Figure 5e‐II) and relaxation speeds (relaxation time ≈ 64 ms, Figure 5e‐III). The simple electro‐optic device demonstrates that our high birefringence polar LC materials can achieve significant transmittance/phase modulation with a drastically reduced electric field, showing great potential for applications in next‐generation LC electro‐optic devices.

## 3 Conclusion

In summary, we developed a library of LC molecules containing over 60 molecules possessing large dipole moments (>9 D). Notably, tolane‐like π‐electron conjugated mesogens with high electronic polarizability were introduced into the library. We found the head groups significantly influence the phase behaviors of LC molecules, with polar LC phases observed exclusively in the DIO and AlkOM groups. In particular, the AlkOM group, characterized by its highly distorted conformation, effectively suppresses crystallization and broadens the temperature window of polar LC phases, potentially offering a general strategy for stabilizing polar LC phases at low temperatures. Regarding the birefringence dependence on chemical structure, introducing triple bonds significantly enhances the birefringence of polar LCs, whereas the ─CF_2_O─ linker or ─CF_3_ end group notably reduces this property. Across various LC phases, a step‐like increase in birefringence is commonly observed during the N – N_F_ and N_F_ – SmA_F_ transition, reflecting a progressive enhancement in birefringence from N to N_F_ and further to SmA_F_. However, high birefringence is consistently accompanied by increased dispersion across different wavelengths. Through multi‐component mixing, we developed N_F_ LC materials that exhibit high birefringence (≈ 0.4 @550 nm), good phase stability near room temperature, and an expanded phase temperature window. The significantly enhanced birefringence performance and the substantial reduction in the driving electric field provide polar LCs with greater potential for triggering innovations in LC electro‐optic devices, such as high‐frame‐rate dynamic displays (wearable AR/VR), high‐efficiency spatial light field modulators, etc.

## Conflict of Interest

The authors declare no conflict of interest.

## Supporting information



Supporting Information

## Data Availability

The data that support the findings of this study are available in the supplementary material of this article.
