# Supplementary material for: Ferroelectric Nematic Liquid Crystals Showing High Birefringence
Source: Adv Sci (Weinh). 2025 Jan 13;12(9):2414317. doi: 10.1002/advs.202414317 (PMC11884546; doi:10.1002/advs.202414317)
Supplement: Supplementary file 1 — Supporting Information [file ADVS-12-2414317-s001.docx]

Supporting Information

Ferroelectric nematic liquid crystals showing high birefringence

Yaohao Song, Xiang Huang, Xinxin Zhang, Minghui Deng, Satoshi Aya, and Mingjun Huang*

Y. Song, X. Huang, X. Zhang, M. Deng, S. Aya, M. Huang

South China Advanced Institute for Soft Matter Science and Technology, School of Emergent Soft Matter, South China University of Technology, Guangzhou 510640, China
E-mail: huangmj25@scut.edu.cn

S. Aya, M. Huang
Guangdong Provincial Key Laboratory of Functional and Intelligent Hybrid Materials and Devices, Guangdong Basic Research Center of Excellence for Energy and Information Polymer Materials, South China University of Technology, Guangzhou 510640, China

**Content list:**

Materials and methods

Figure S1-S30

Table S1-S6

Synthesis

Scheme S1-S3

^1^H, ^19^F and ^13^C NMR spectra

Scheme S4-S78

Reference

**Materials and Methods**

***Materials***

Unless stated below, all materials and reagents were obtained from Energy Chemical, Bidepharm, Innochem, Adamas, Sigma-Aldrich, TCI, and Aladdin. All commercial chemicals and solvents were used as received without purification unless stated otherwise.

***Methods***

Nuclear Magnetic Resonance (NMR) Spectroscopy

^1^H, ^19^F and ^13^C NMR spectra of the samples were obtained in CDCl_3_ (Innochem, 99.8 atom % D, with 0.03%(v/v) TMS) solvents utilizing JASTEC JMTC-500/54/JJ 500 MHz NMR spectrometer. ^1^H NMR spectra were referenced to the residual proton impurities in CDCl_3_ at δ 7.26 ppm and ^13^C NMR spectra were referenced to ^13^CDCl_3_ at δ 77.16 ppm.

Differential Scanning Calorimetry (DSC)

The thermal properties of all the samples were characterized by utilizing a TA Instruments DSC 2500 with an Intercooler 2P apparatus. The temperature and heat flow scales were calibrated at different heating and cooling rates (10 K min^-1^) using a series of standard materials. For each run of the experiments, the initial mass of the samples used was about 2 -3 mg under nitrogen flow.

Thermogravimetric analysis (TGA)

TGA was monitored on a TGA5500 (TA, USA) in nitrogen with a heating rate of 10 °C/min and a temperature range of 30 - 600 °C.

Density Functional Theory (DFT) Calculation

All DFT calculations were performed with the Gaussian 16 series of programs (Gaussian 16W, Version 1.1). The DFT method of opt b3lyp/6-311+g(d,p) geom=connectivity empiricaldispersion=gd3 basis set was used.

Birefringence (Δn)

The wavelength retardation of the LCs was measured by POM (Olympus BX51) with the Berek compensator (model from Nichika Corporation), which can be utilized to ascertain the birefringence value of LCs of known thickness. The retardation was recorded under the control of the Instec mK2000 hot stage. The birefringence at different wavelengths is measured by inserting a bandpass filter of the corresponding wavelength into the microscope light source. The filter used is the GCC-2010 series 10 nm narrow bandpass filter from Daheng Optics. The birefringence was measured at wavelengths of 488 nm, 532 nm, 550 nm, 632.8 nm, 650 nm, and 670 nm.

Polarized optical microscope (POM)

The textures of all samples during the cooling process were observed by POM (Olympus BH2). The temperature was controlled by the hot stage (Instec mK2000). Photographs of POM were taken by camera (Nikon D5300).

Dielectric spectroscopy

The dielectric spectroscopy was measured in homemade 20 μm thick ITO-coated natural LC cells, applying an electric field perpendicular to the plane of the ITO glass. The voltage applied to the ITO cell is 50 mV. The dielectric spectroscopy was conducted by using an LCR meter (E4980A, Keysight). The collection of the frequency and temperature sweeping data is automated by homemade software written in Labview.

The polarization switching measurement

The polarization switching measurements (P-E hysteresis loops) were conducted using the ferroelectricity measurement system (TOYO FCE10-S, Japan) combined with a waveform generator (Tabor Electronic, WW5064), high-voltage linear amplifier (pendulum, A800), and analyzer (TOYO, Model 6252).

SHG measurement

We used a fundamental beam from a Q-switched pulsed laser (MPL-III-1064-20µJ) with a central wavelength of 1064 nm, maximum power of 200 mW, pulse duration of 5 ns, and 100 Hz repetition. The p-polarized fundamental beam was directed at LC cells. The SH light is detected in the transmission geometry by a photomultiplier tube (DH-PMT-D100V, Daheng Optics). For the temperature scanning, the SH signal is recorded under the control of a homemade Labview program. The SHG intensity is referenced to a 0.5 mm Y-cut quartz, with the SHG intensity reported as a multiple of the relative quartz intensity (I/I_quartz_). The phase state of the LC material determines the temperature dependence of the SHG intensity. During the cooling process, both the Iso and N phases are non-polar and exhibit no SHG response. In the antiferroelectric N_x_ phase, where the macroscopic polarization is canceled, there is also no SHG response. As the material transitions to the ferroelectric N_F_ and SmA_F_ phases, the development of strong static polarization leads to a significant increase in the SHG signal. Upon crystallization, the SHG intensity diminishes or vanishes entirely.

Sample preparation in LC cell

For POM observation and SHG measurement, samples were introduced to homemade planar LC cells with syn-parallel rubbing conditions. The homemade cells were made with two glass plates, controlled spacers, planar alignment conditions, and a glue seal. For alignment materials, we used KPI-3000 (Shenzhen Haihao Technology Co. Ltd). The rubbing alignment conditions are controlled by Sichuan Shiji Zhongke Photoelectric Technology Co., Ltd liquid crystal alignment rubbing machine (ZKY-LCDZBX-MC). Homemade 20 μm thick natural cells with ITO layer were used in polarization switching and dielectric spectroscopy measurements. To ensure the accuracy of the birefringence data, the birefringence measurement used commercial EHC parallel alignment 2 μm LC cells (KSRO-02/B111P7NSS05).


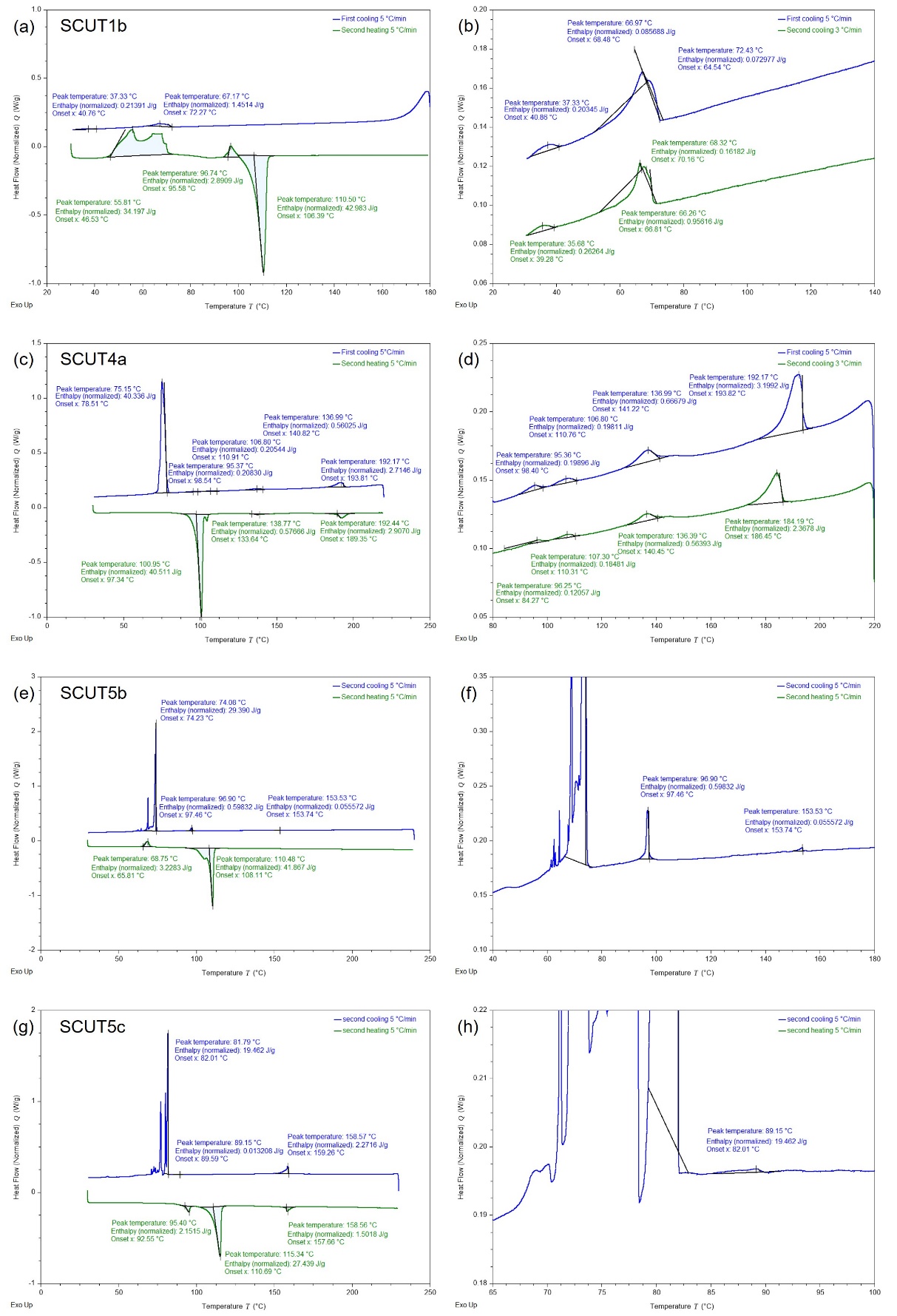


**Figure S1.** The DSC profiles of polar LCs. The entire cooling process profiles and the enlarged profiles of (a-b) SCUT1b; (c-d) SCUT4a; (e-f) SCUT5b; (g-h) SCUT5c. Cooling rate: 5 K/min. For the DSC profiles of SCUT1a and SCUT5a, please refer to our previous articles ^[1]^.


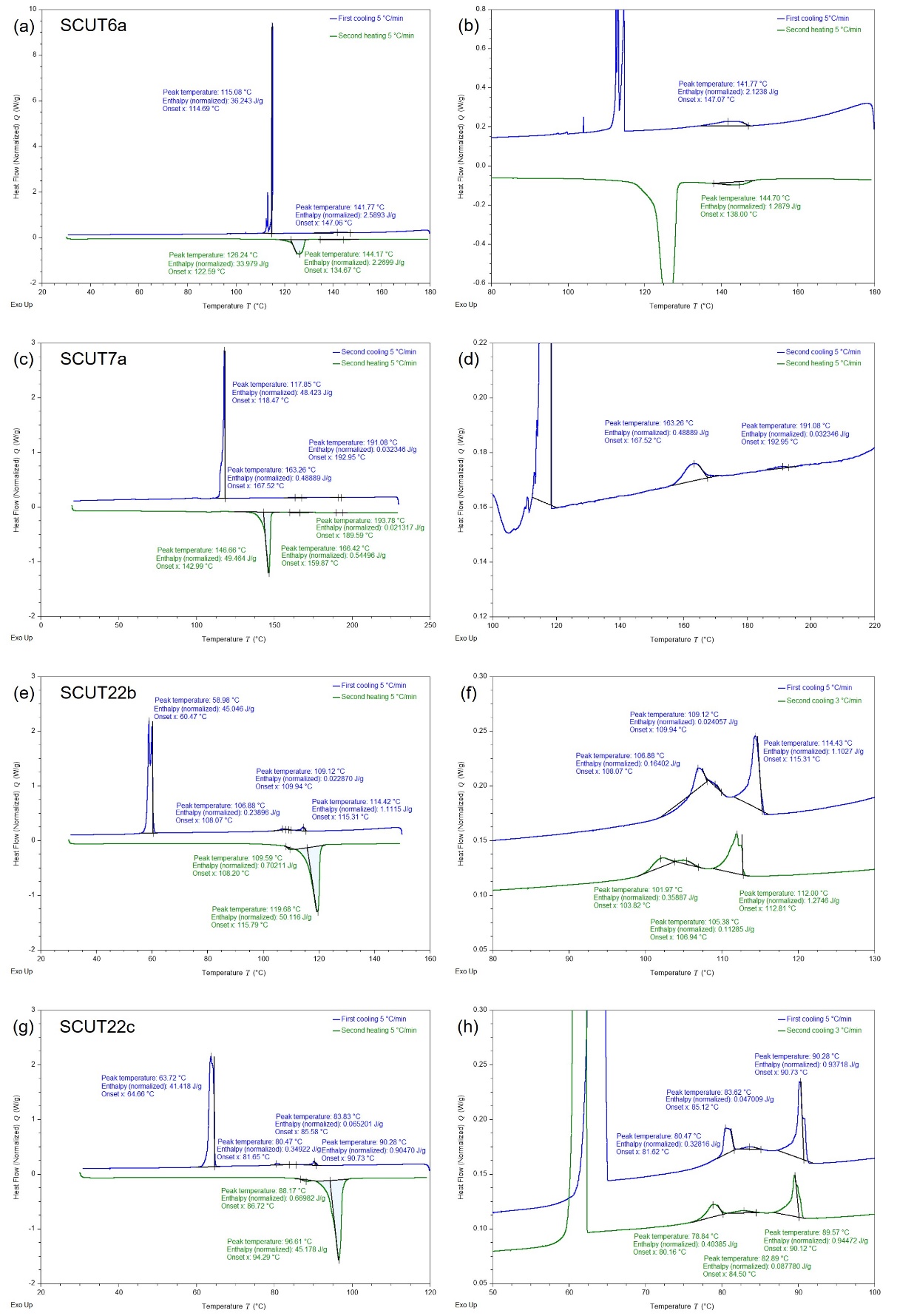


**Figure S2.** The DSC profiles of polar LCs. The entire cooling process profiles and the enlarged profiles of (a-b) SCUT6a; (c-d) SCUT7a; (e-f) SCUT22b; (g-h) SCUT22c. Cooling rate: 5 K/min.


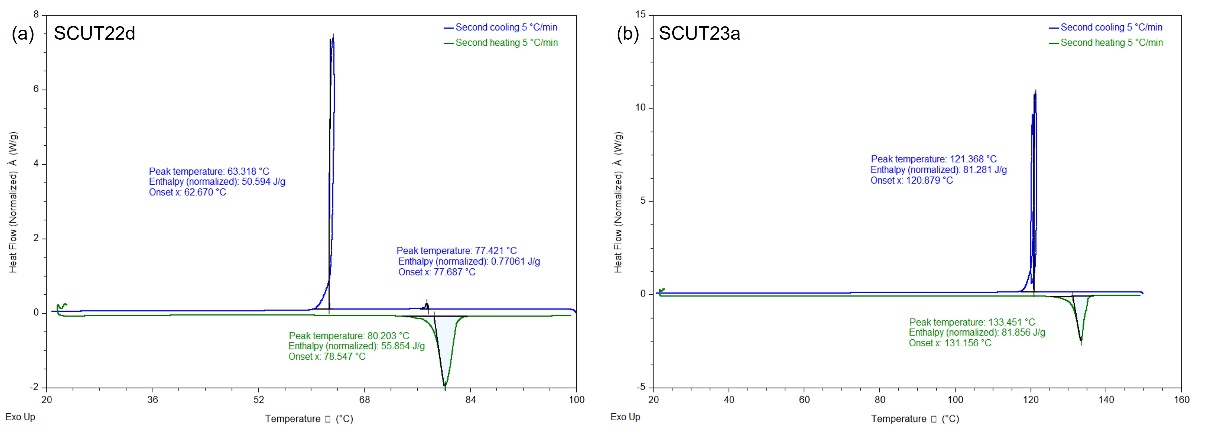


**Figure S3.** The DSC profiles of polar LCs. The entire cooling process profiles of (a) SCUT22d; (b) SCUT23a. Cooling rate: 5 K/min.


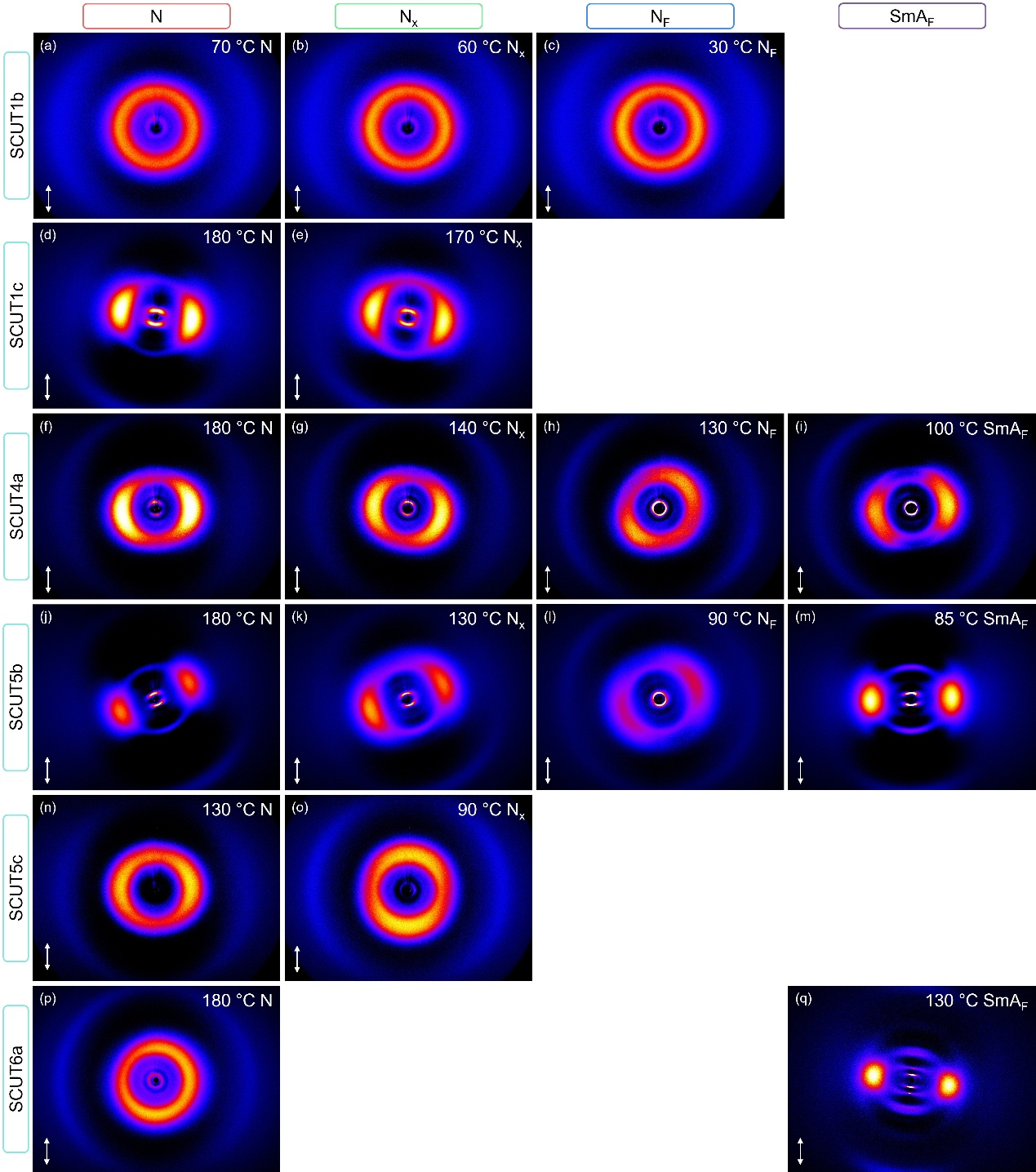


**Figure S4.** 2D XRD patterns of polar LCs during cooling. Each row, from top to bottom, corresponds to an individual LC molecule: (a-c) SCUT1b, (d-e) SCUT1c, (f-i) SCUT4a, (j-m) SCUT5b, (n-o) SCUT5c, and (p-q) SCUT6a. Each column represents a different LC phase, arranged from left to right as N, N_x_, N_F_, and SmA_F_. Blank areas indicate the absence of a particular LC phase for the respective molecule. The axis at the bottom left indicates the capillary direction in which the sample was melt-loaded. Even for the same LC phase, different samples can exhibit varying sensitivities to the capillary tube’s orientational effect, resulting in notable differences in the observed patterns.


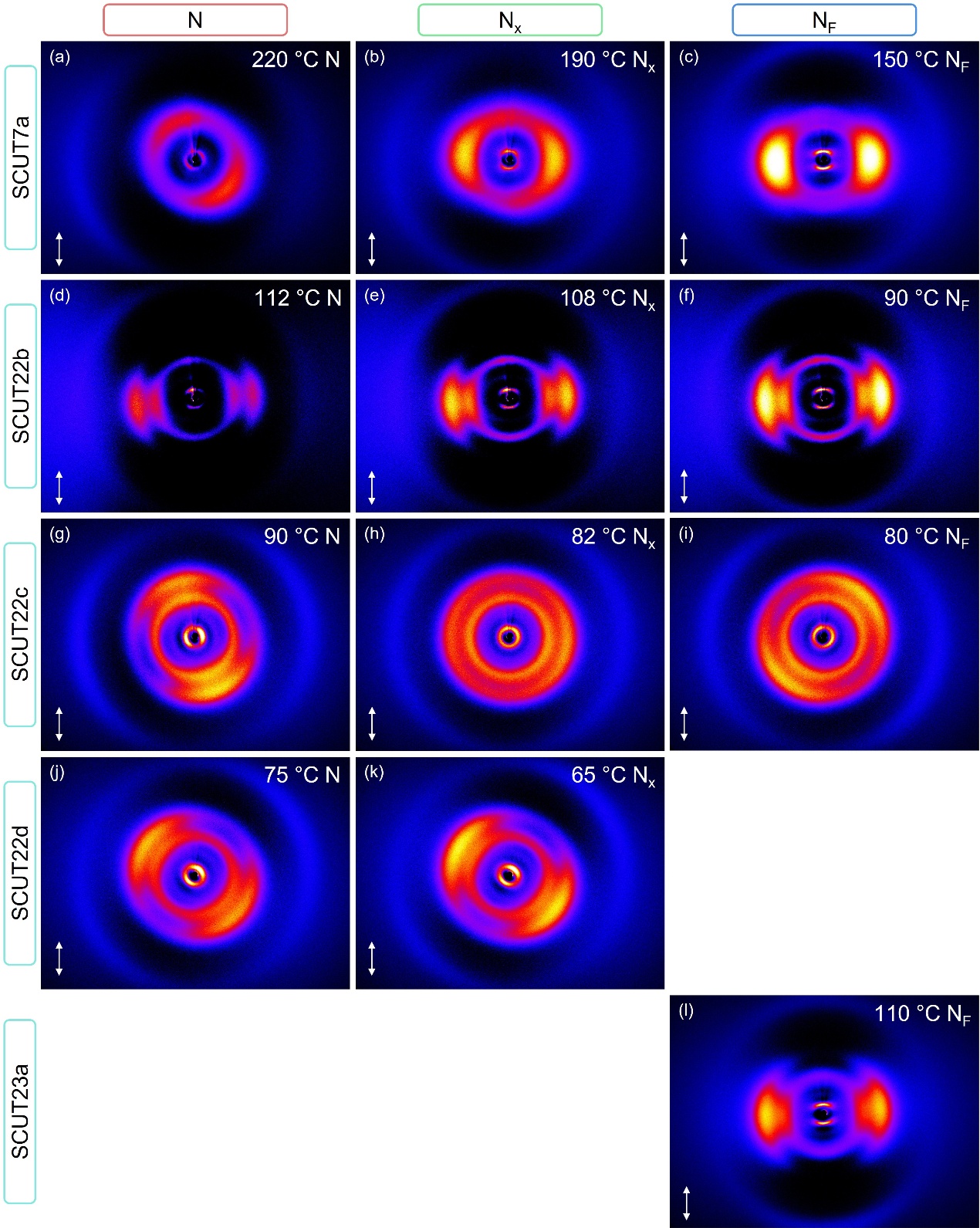


**Figure S5.** 2D XRD patterns of polar LCs during cooling. Each row, from top to bottom, corresponds to an individual LC molecule: (a-c) SCUT7a, (d-f) SCUT22b, (g-i) SCUT22c, (j-k) SCUT22d, (l) SCUT23a. Each column represents a different LC phase, arranged from left to right as N, N_x_, and N_F_. Blank areas indicate the absence of a particular LC phase for the respective molecule. The axis at the bottom left indicates the capillary direction in which the sample was melt-loaded.


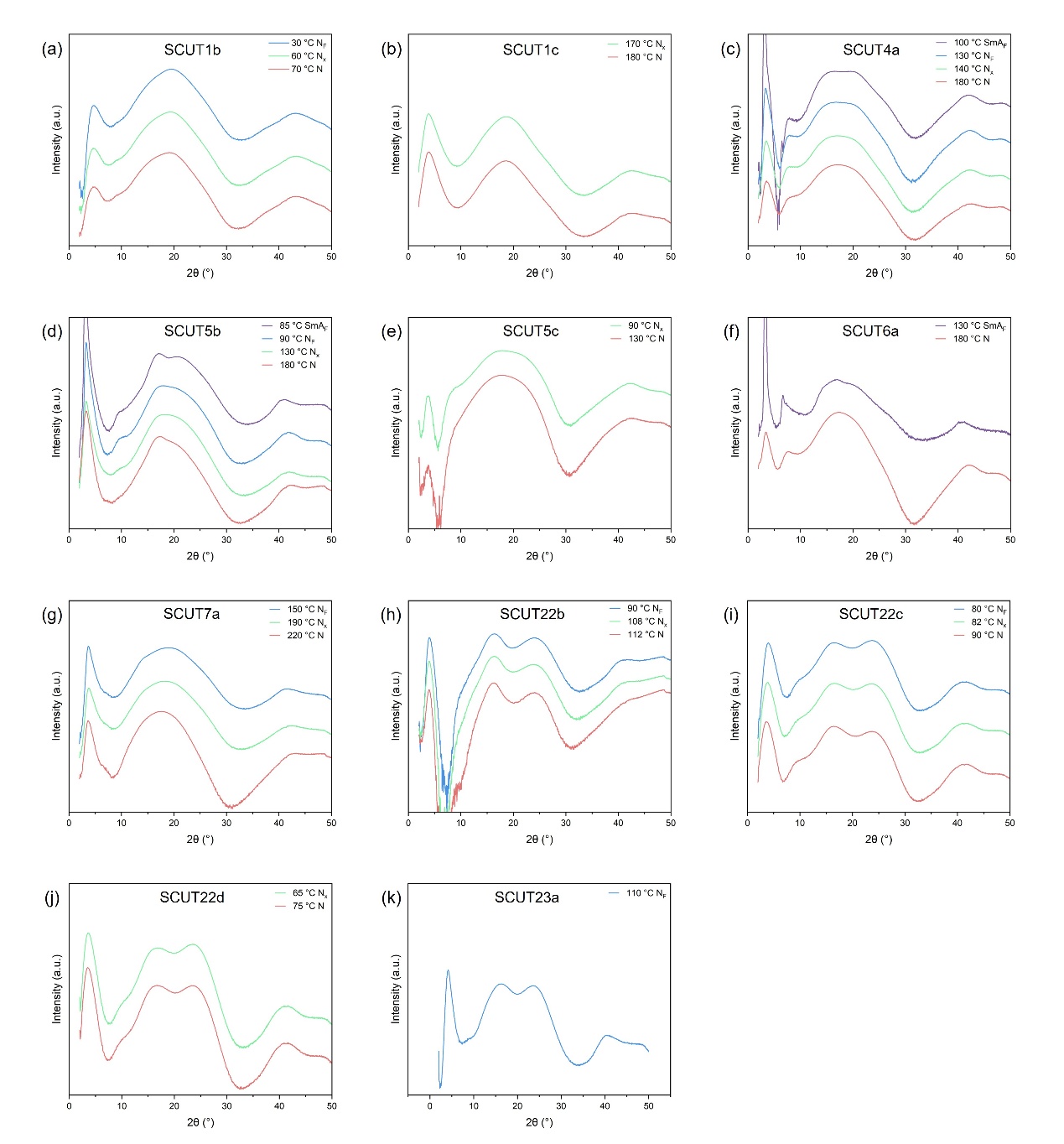


**Figure S6.** 1D XRD curve obtained by integrating the 2D XRD pattern from Figure S4-S5. (a) SCUT1b, (b) SCUT1c, (c) SCUT4a, (d) SCUT5b, (e) SCUT5c, (f) SCUT6a, (g) SCUT7a, (h) SCUT22b, (i) SCUT22c, (j) SCUT22d, and (k) SCUT23a. For the 2D and 1D XRD patterns of SCUT1a and SCUT5a, please refer to our previous articles ^[1]^.


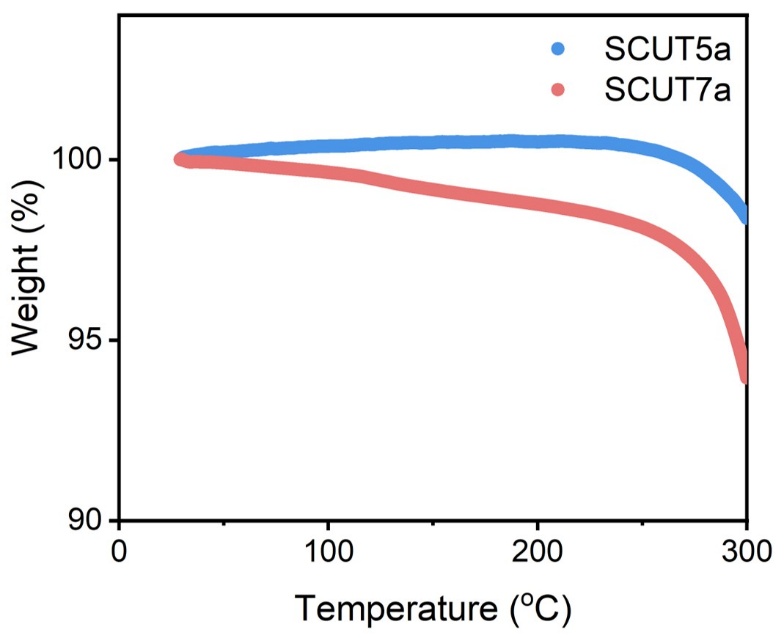


**Figure S7.** TGA curves of SCUT5a and SCUT7a. The chemical structures of SCUT5a and SCUT7a differ only in the position of the triple bond. The triple bond is placed closer to the terminal strong electron-withdrawing group -CN, which leads to the poorer thermal stability of SCUT7a and slowly decomposes during the heating process.


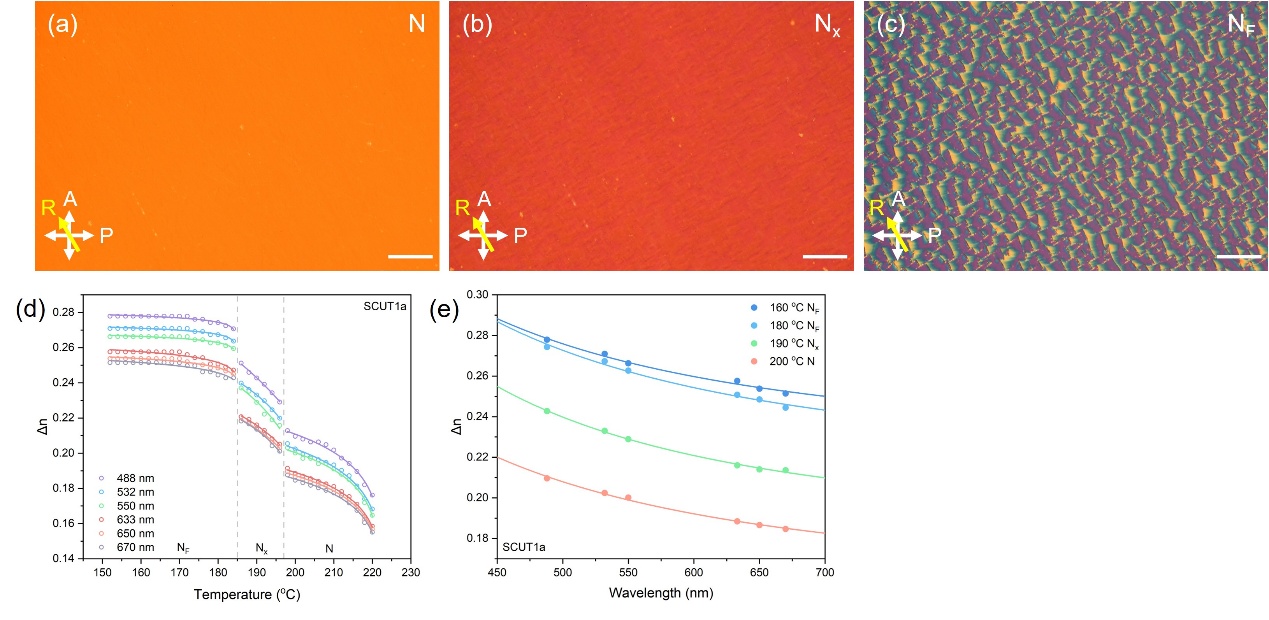


**Figure S8.** Properties of the SCUT1a molecule. (a-c) POM texture during the cooling process. (a) 210 °C, N phase; (b) 190 °C, N_x_ phase; (c) 170 °C, N_F_ phase. Scale bar: 100 μm. (d) During cooling temperature dependencies of birefringence at different wavelengths (488 nm, 532 nm, 550 nm, 633 nm, 650 nm, 670 nm). The curve in the figure is fitted by equation (1) in the main text. (e) Variation of birefringence with wavelength in different LC phases. The curve in the figure is fitted by formula (3) in the main text. Syn-parallel alignment EHC LC cell, cell thickness: 2.3 μm. For more detailed properties of SCUT1a such as SHG, dielectric constant, P-E hysteresis loop, etc., please refer to our previous articles ^[1]^.


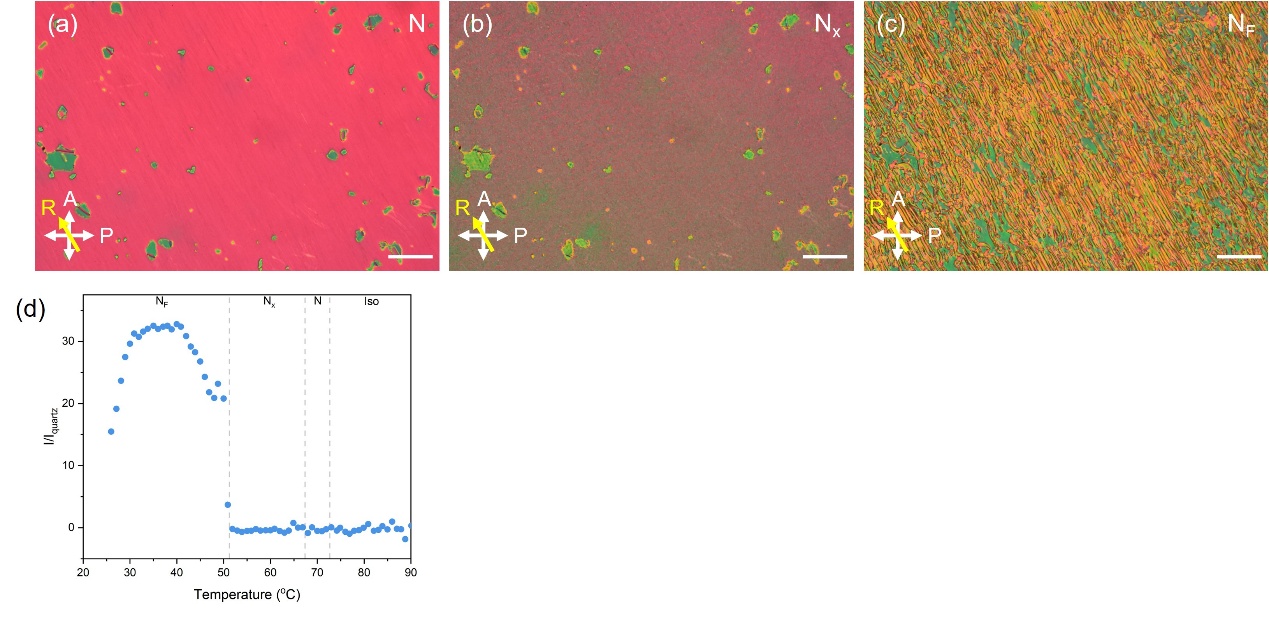


**Figure S9.** Properties of the SCUT1b molecule. (a-c) POM texture during the cooling process. (a) 70 °C, N phase; (b) 60 °C, N_x_ phase; (c) 30 °C, N_F_ phase. Scale bar: 100 μm. (d) Temperature dependence of SHG signal intensity. POM observation and SHG measurement using syn-parallel alignment homemade LC cell, cell thickness: 10.7 μm. The SCUT1b molecule exhibits a high density of defect lines in the N_F_ phase, which cannot be suppressed even in very thin cells. The excessive defect lines introduce significant errors in birefringence. As a result, birefringence measurement was not performed on this molecule to avoid potential inaccuracies in the data.


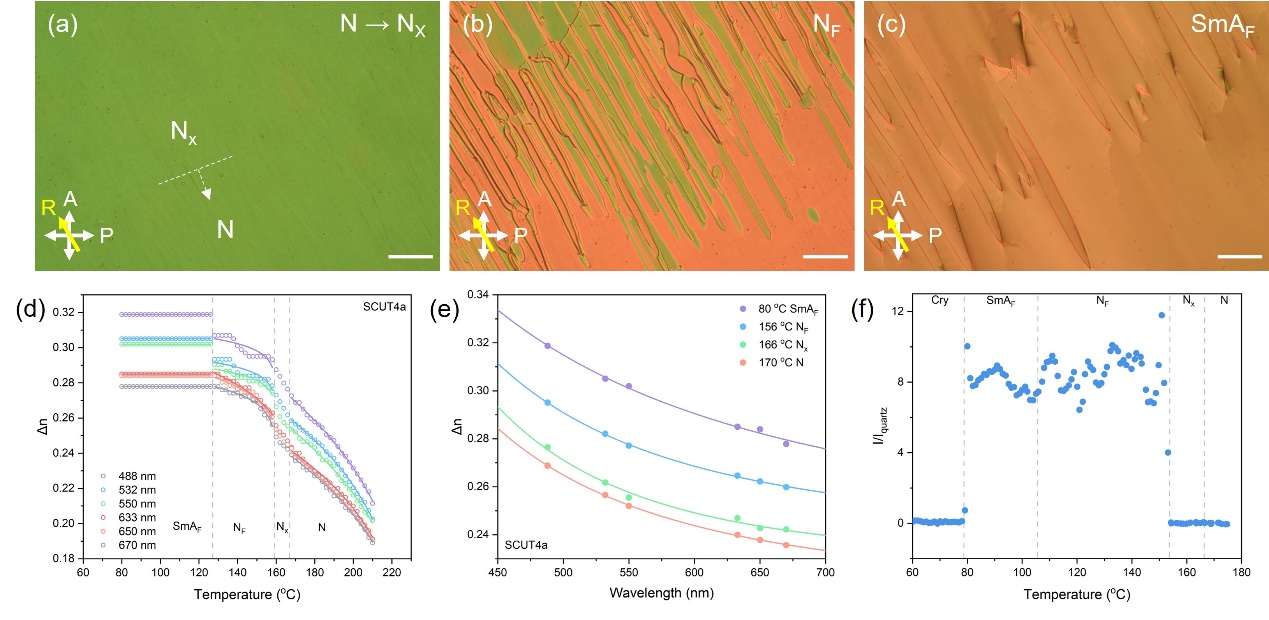


**Figure S10.** Properties of the SCUT4a molecule. (a-c) POM texture during the cooling process. (a) 146 °C, N phase transition to N_x_ phase; (b) 130 °C, N_F_ phase; (c) 90 °C, SmA_F_ phase. Scale bar: 100 μm. (d) During cooling, temperature dependencies of birefringence at different wavelengths (488 nm, 532 nm, 550 nm, 633 nm, 650 nm, 670 nm). The curve in the figure is fitted by equation (1) in the main text. (e) Variation of birefringence with wavelength in different LC phases. The curve in the figure is fitted by formula (3) in the main text. Birefringence measurement using syn-parallel alignment EHC LC cell, cell thickness: 2.2 μm. (f) Temperature dependence of SHG signal intensity. POM observation and SHG measurement using syn-parallel alignment homemade LC cell, cell thickness: 8.1 μm.


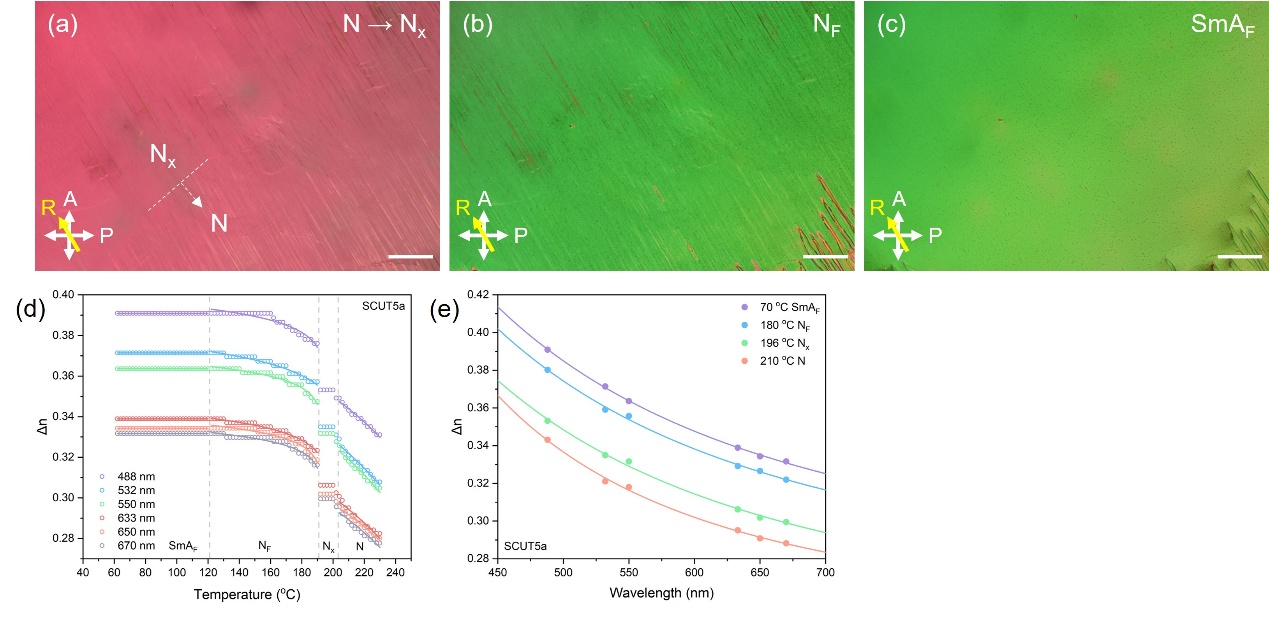


**Figure S11.** Properties of the SCUT5a molecule. (a-c) POM texture during the cooling process. (a) 202 °C, N phase transition to N_x_ phase; (b) 130 °C, N_F_ phase; (c) 70 °C, SmA_F_ phase. POM observation using syn-parallel alignment homemade LC cell, cell thickness: 3.8 μm. Scale bar: 100 μm. (d) During cooling, temperature dependencies of birefringence at different wavelengths (488 nm, 532 nm, 550 nm, 633 nm, 650 nm, 670 nm). The curve in the figure is fitted by equation (1) in the main text. (e) Variation of birefringence with wavelength in different LC phases. The curve in the figure is fitted by formula (4) in the main text. Birefringence measurement using syn-parallel alignment EHC LC cell, cell thickness: 2.3 μm. For more detailed properties of SCUT5a such as SHG, dielectric constant, P-E hysteresis loop, etc., please refer to our previous articles ^[2]^.


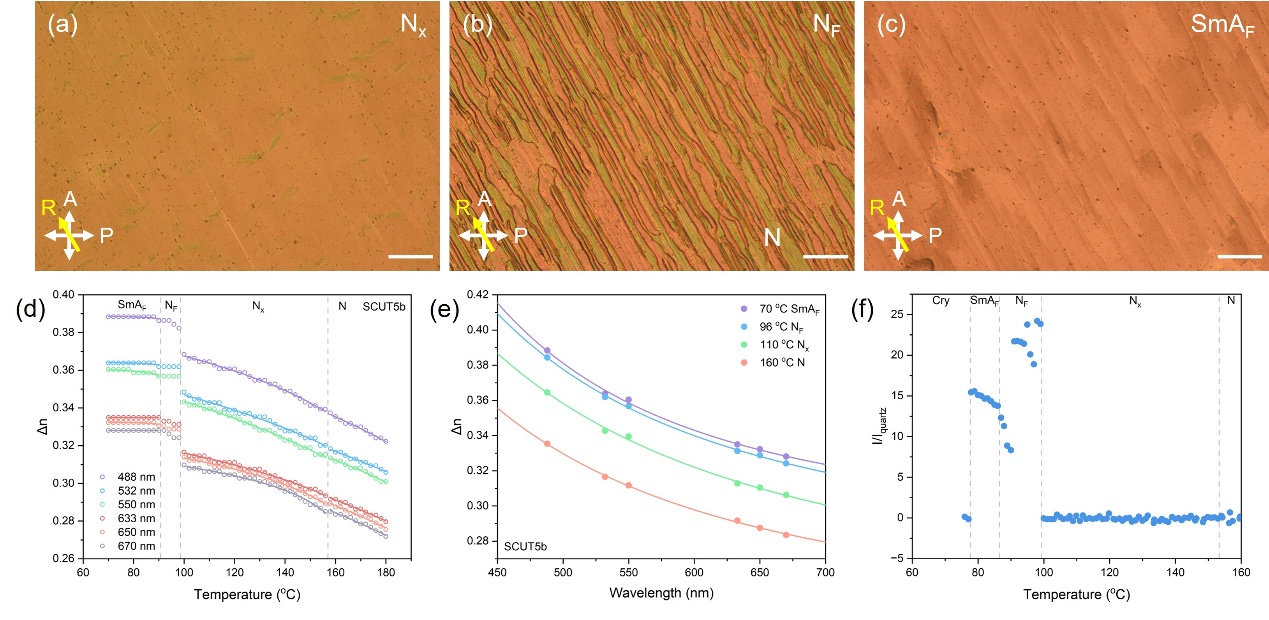


**Figure S12.** Properties of the SCUT5b molecule. (a-c) POM texture during the cooling process. (a) 130 °C, N_x_ phase; (b) 92 °C, N_F_ phase; (c) 75 °C, SmA_F_ phase. Scale bar: 100 μm. (d) During cooling, temperature dependencies of birefringence at different wavelengths (488 nm, 532 nm, 550 nm, 633 nm, 650 nm, 670 nm). The curve in the figure is fitted by equation (1) in the main text. (e) Variation of birefringence with wavelength in different LC phases. The curve in the figure is fitted by formula (3) in the main text. Birefringence measurement using syn-parallel alignment EHC LC cell, cell thickness: 2.5 μm. (f) Temperature dependence of SHG signal intensity. POM observation and SHG measurement using syn-parallel alignment homemade LC cell, cell thickness: 8.1 μm.


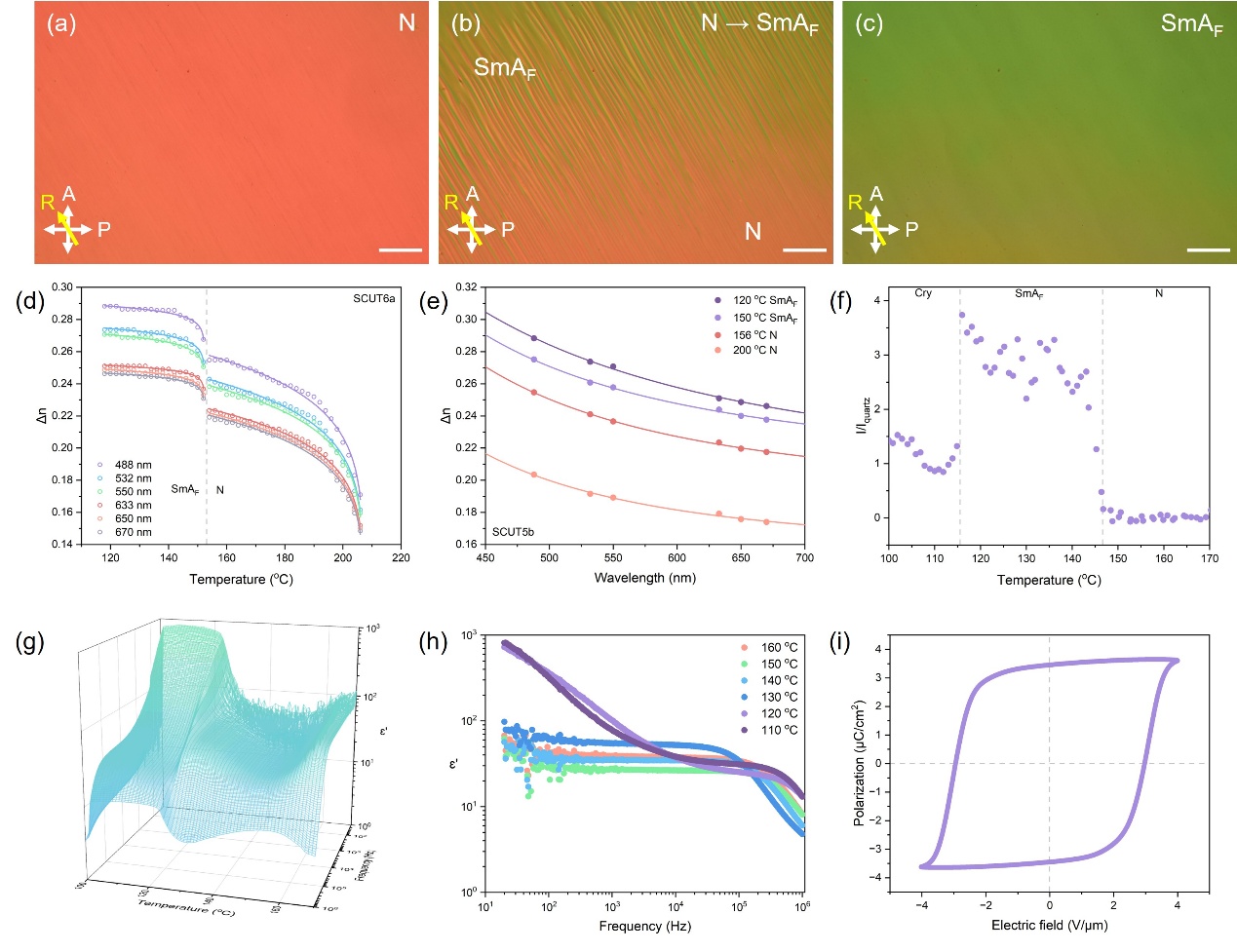


**Figure S13.** Properties of the SCUT6a molecule. (a-c) POM texture during the cooling process. (a) 160 °C, N phase; (b) 151 °C, N phase transition to SmA_F_ phase; (c) 150 °C, SmA_F_ phase. Scale bar: 100 μm. (d) During cooling, temperature dependencies of birefringence at different wavelengths (488 nm, 532 nm, 550 nm, 633 nm, 650 nm, 670nm). The curve in the figure is fitted by equation (1) in the main text. (e) Variation of birefringence with wavelength in different LC phases. The curve in the figure is fitted by formula (3) in the main text. Birefringence measurement using syn-parallel alignment EHC LC cell, cell thickness: 2.2 μm. (f) Temperature dependence of SHG signal intensity. POM observation and SHG measurement using syn-parallel alignment homemade LC cell, cell thickness: 6.2 μm. (g) Temperature and frequency dependence of the dielectric constant. (h) Frequency dependence of dielectric constant at selected temperatures in (g). (i) The P-E hysteresis loop measured in the N_F_ phase (140 °C). V_p_ = 80 V, f = 200 Hz. Dielectric constant and P-E hysteresis loop measured in ITO natural cell, cell thickness: 20 μm.


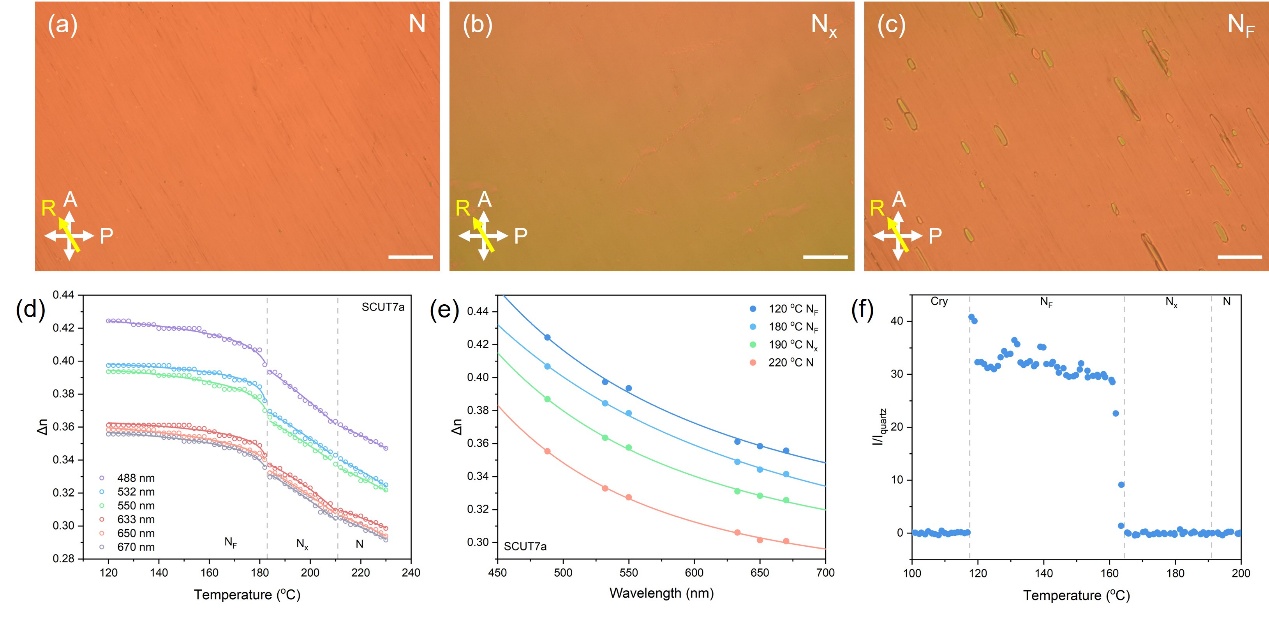


**Figure S14.** Properties of the SCUT7a molecule. (a-c) POM texture during the cooling process. (a) 220 °C, N phase; (b) 200 °C, N_x_ phase; (c) 130 °C, N_F_ phase. Scale bar: 100 μm. (d) During cooling, temperature dependencies of birefringence at different wavelengths (488 nm, 532 nm, 550 nm, 633 nm, 650 nm, 670 nm). The curve in the figure is fitted by equation (1) in the main text. (e) Variation of birefringence with wavelength in different LC phases. The curve in the figure is fitted by formula (3) in the main text. Birefringence measurement using syn-parallel alignment EHC LC cell, cell thickness: 2.3 μm. (f) Temperature dependence of SHG signal intensity. POM observation and SHG measurement using syn-parallel alignment homemade LC cell, cell thickness: 7.2 μm.


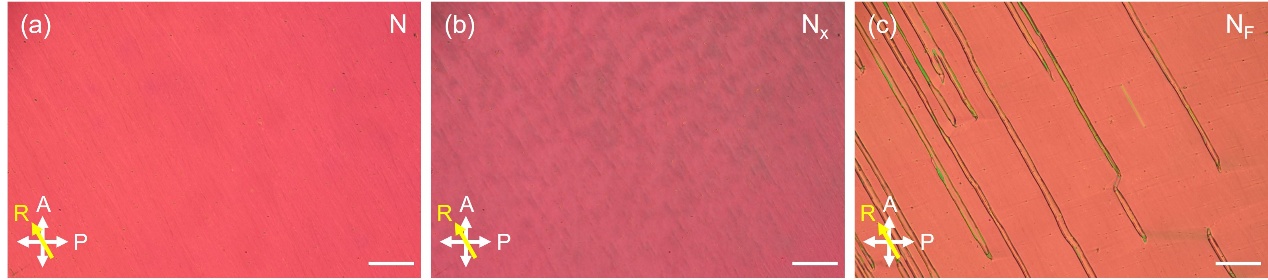


**Figure S15.** POM texture of the SCUT22b molecule. (a-c) POM texture during the cooling process. (a) 115 °C, N phase; (b) 114 °C, N_x_ phase; (c) 90 °C, N_F_ phase. Syn-parallel alignment, cell thickness: 4.7 μm. Scale bar: 100 μm.


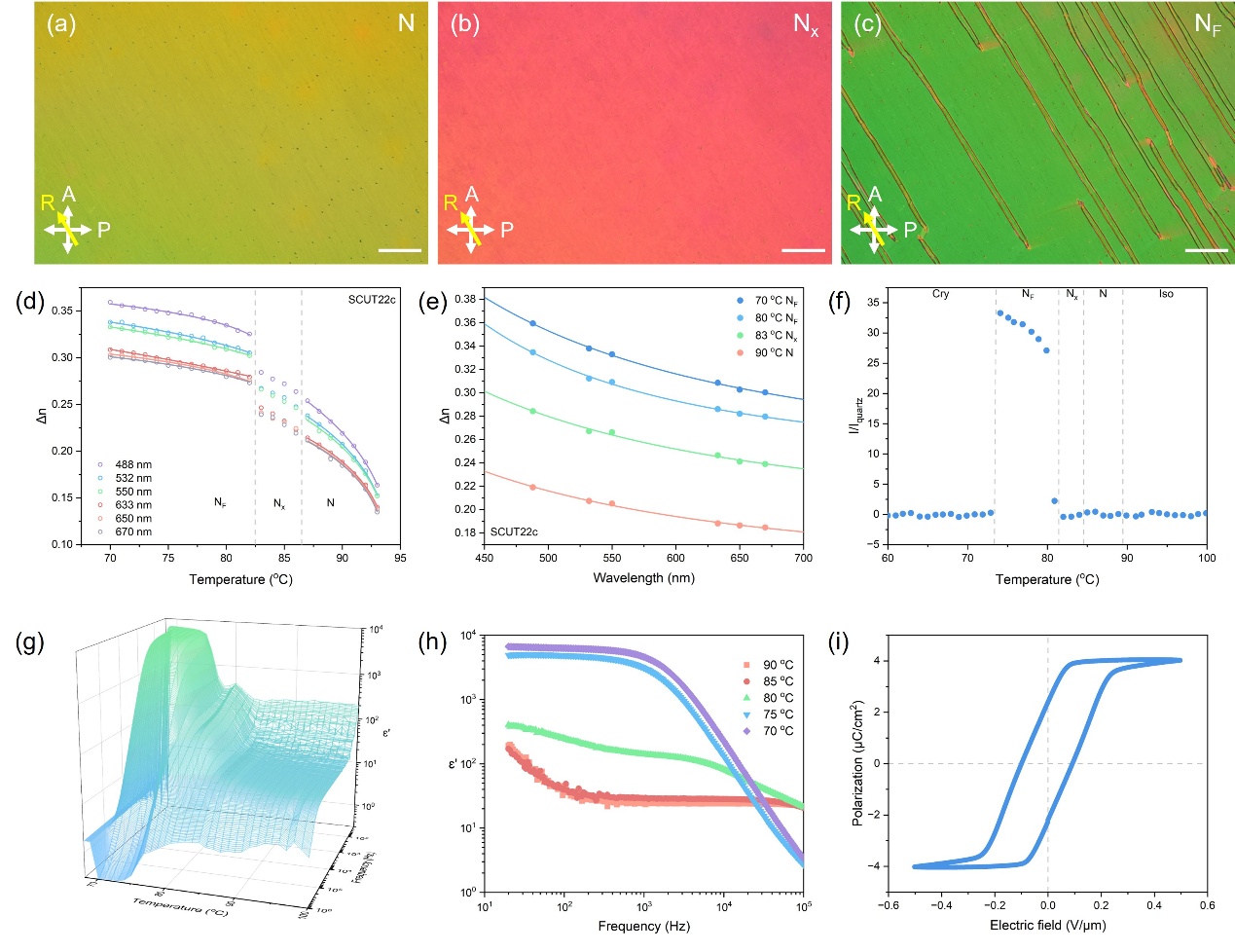


**Figure S16.** Properties of the SCUT22c molecule. (a-c) POM texture during the cooling process. (a) 90 °C, N phase; (b) 86 °C, N_x_ phase; (c) 70 °C, N_F_ phase. Scale bar: 100 μm. (d) During cooling, temperature dependencies of birefringence at different wavelengths (488 nm, 532 nm, 550 nm, 633 nm, 650 nm, 670 nm). The curve in the figure is fitted by equation (1) in the main text. (e) Variation of birefringence with wavelength in different LC phases. The curve in the figure is fitted by formula (3) in the main text. Birefringence measurement using syn-parallel alignment EHC LC cell, cell thickness: 2.6 μm. (f) Temperature dependence of SHG signal intensity. POM observation and SHG measurement using syn-parallel alignment homemade LC cell, cell thickness: 4.3 μm. (g) Temperature and frequency dependence of the dielectric constant. (h) Frequency dependence of dielectric constant at selected temperatures in (g). (i) The P-E hysteresis loop measured in the N_F_ phase (70 °C). V_p_ = 10 V, f = 200 Hz. Dielectric constant and P-E hysteresis loop measured in ITO natural cell, cell thickness: 20 μm.


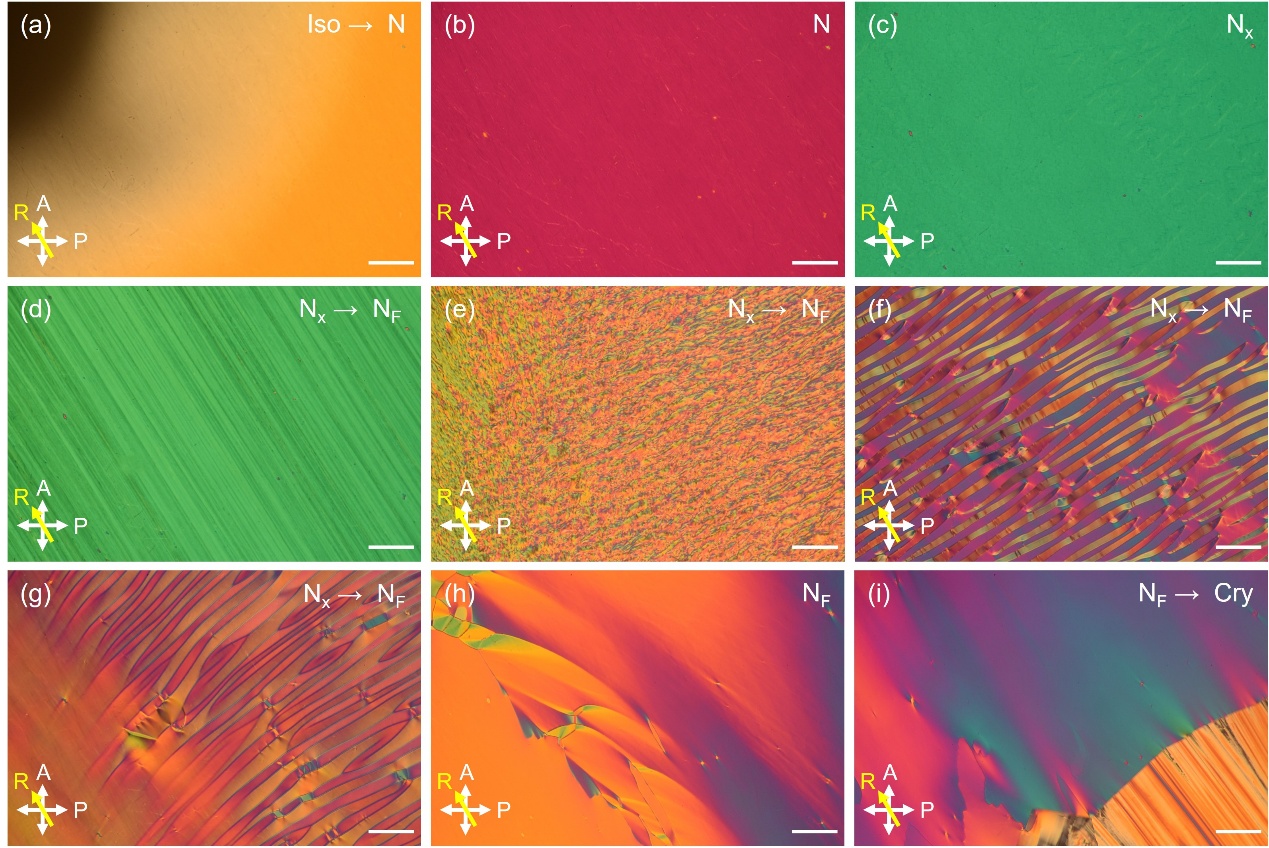


**Figure S17.** POM texture of the SCUT22d molecule. (a-f) POM texture during the cooling process. (a) 79 °C, phase transition from Iso to N phase; (b) 75 °C, N phase; (c) 65 °C, N_x_ phase; (d) 62 °C, phase transition from N_x_ to N_F_ phase; (e) 59 °C, phase transition from N_x_ to N_F_ phase; (f) 58.6 °C, phase transition from N_x_ to N_F_ phase; (g) 58 °C, phase transition from N_x_ to N_F_ phase; (h) 57 °C, N_F_ phase; (d) 56 °C, phase transition from N_F_ to crystallization. Syn-parallel alignment, cell thickness: 2.6 μm. Scale bar: 100 μm. The SCUT22d molecule can present a stable N_F_ phase during the cooling process, but the N_F_ phase temperature window is narrow.


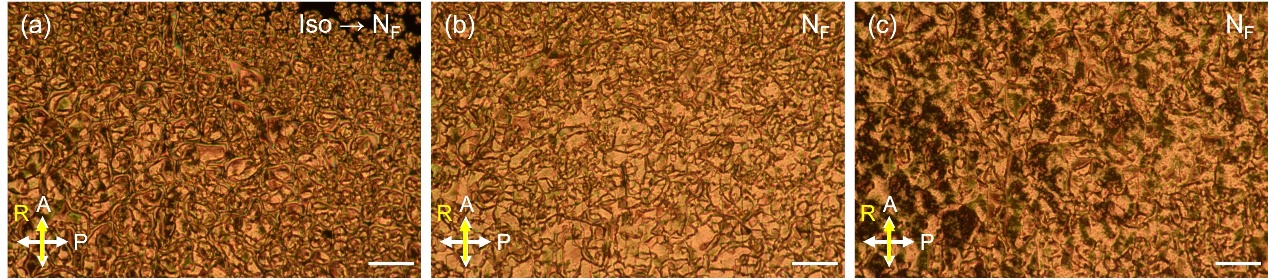


**Figure S18.** POM texture of the SCUT23a molecule. (a-c) POM texture during the cooling process. (a) 113 °C, phase transition from Iso to N phase; (b) 110 °C, N_F_ phase; (c) 109 °C, N_F_ phase. Syn-parallel alignment, cell thickness: 6.8 μm. Scale bar: 100 μm. During the cooling process, SCUT23a molecules directly transition from the Iso to the N_F_ phase through nucleation and can present a stable N_F_ phase, but the N_F_ phase temperature window is narrow.


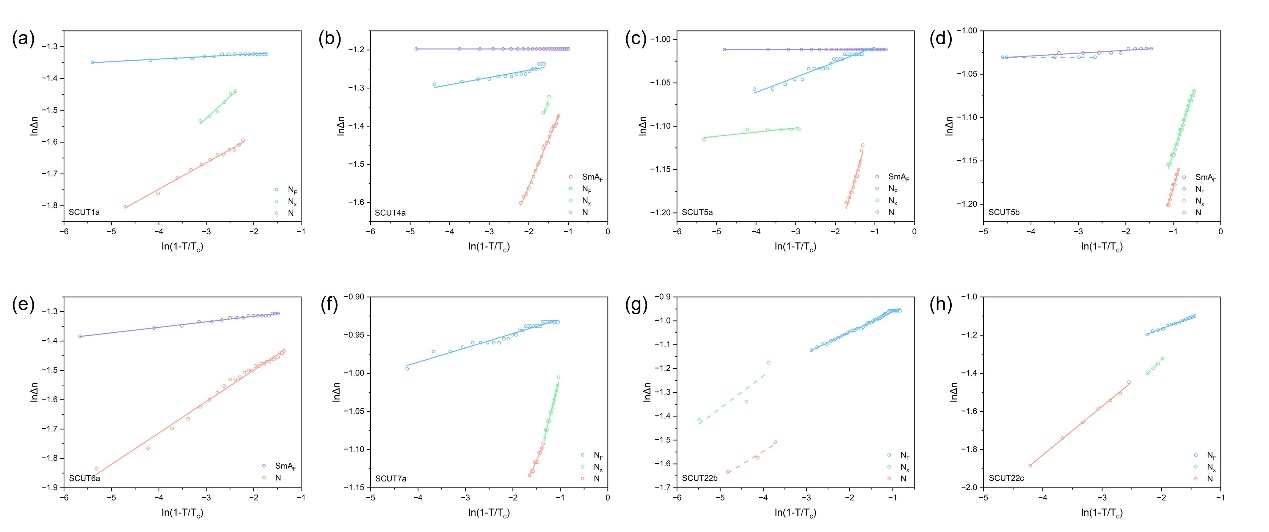


**Figure S19.** Taking 550 nm as an example, the temperature dependence of birefringence is logarithmized and piecewise fitted using equation (2). (a-h) individual display of the fitting results of all polar liquid crystal molecules in the molecular library. Respectively, (b) SCUT1a; (c) SCUT4a; (d) SCUT5a; (e) SCUT5b; (f) SCUT6a; (g) SCUT7a; (h) SCUT22b; (i) SCUT22c. A normal reliable linear fit uses a straight solid line. The LC phase temperature window of some molecules is too narrow, and the fitting straight line may have a certain error, which is represented by a dotted line. It can be seen that the slope of each molecule in the N_F_ and SmA_F_ phases is significantly smaller than that in the N and N_x_ phases, corresponding to the rule that the fitting parameter β is smaller in the polar LC phase.


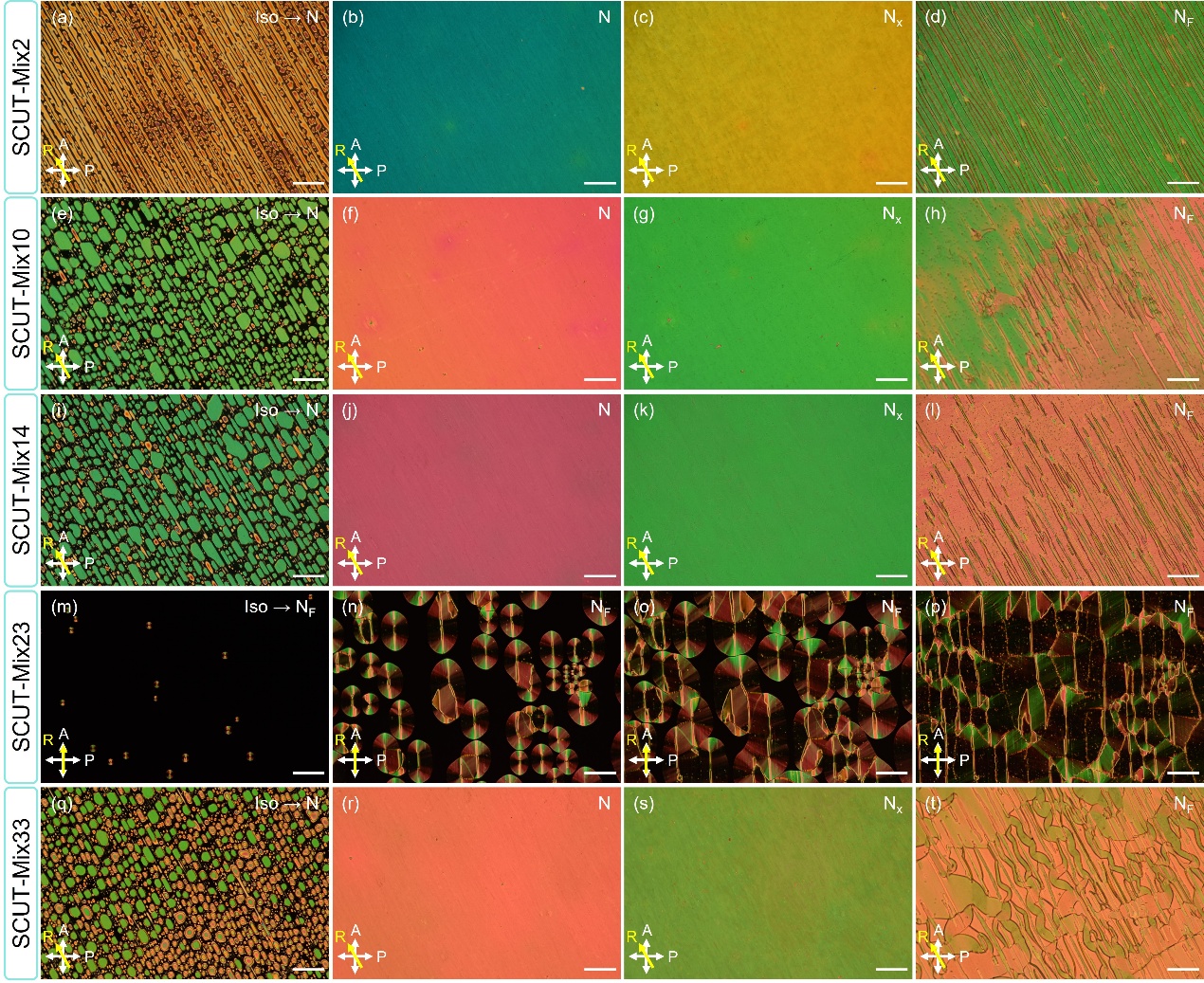


**Figure S20.** POM texture of polar LC mixture materials. (a-d) POM texture of SCUT-Mix2 during the cooling process. (a) 104 °C, phase transition from Iso to N phase; (b) 102 °C, N phase; (c) 99 °C, N_x_ phase; (d) 60 °C, N_F_ phase. Syn-parallel alignment, cell thickness: 3.5 μm. (e-h) POM texture of SCUT-Mix10 during the cooling process. (e) 202 °C, phase transition from Iso to N phase; (f) 190 °C, N phase; (g) 161 °C, N_x_ phase; (h) 30 °C, N_F_ phase. Syn-parallel alignment, cell thickness: 4.4 μm. (i-l) POM texture of SCUT-Mix14 during the cooling process. (i) 227 °C, phase transition from Iso to N phase; (j) 180 °C, N phase; (k) 145 °C, N_x_ phase; (l) 28 °C, N_F_ phase. Syn-parallel alignment, cell thickness: 4.4 μm. (m-p) POM texture of SCUT-Mix23 during the cooling process. (m) 70 °C, phase transition from Iso to N_F_ phase; (n) 65 °C, N_F_ phase; (o) 60 °C, N_F_ phase; (p) 35 °C, N_F_ phase. Syn-parallel alignment, cell thickness: 4.3 μm. (q-t) POM texture of SCUT-Mix33 during the cooling process. (q) 133 °C, phase transition from Iso to N phase; (r) 115 °C, N phase; (s) 103 °C, N_x_ phase; (t) 28 °C, N_F_ phase. Syn-parallel alignment, cell thickness: 7.9 μm. Scale bar: 100 μm.


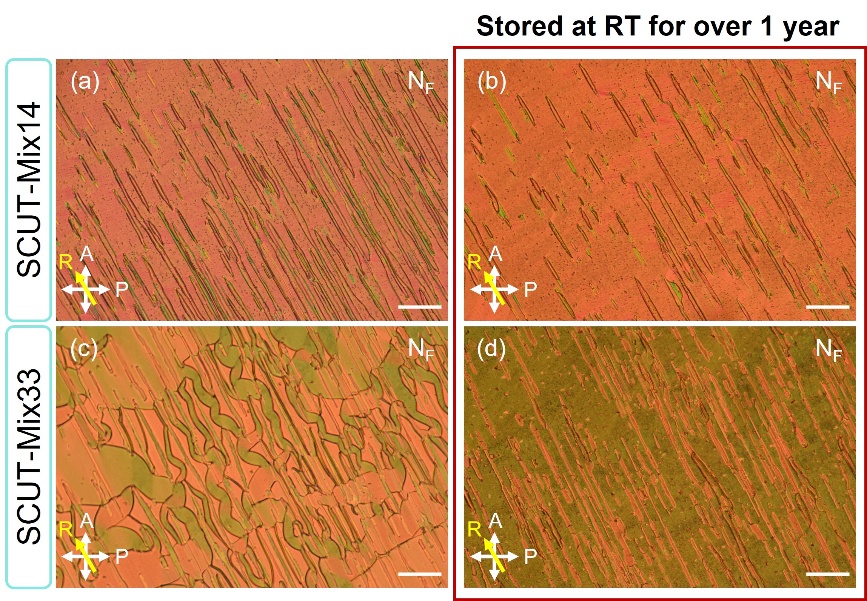


**Figure S21.** The POM evidence for the excellent stability of the polar LC mixtures. The POM textures of (a) SCUT-Mix14 and (c) SCUT-Mix33 observed after cooling to room temperature (RT). The POM textures of the polar LC mixtures of (b) SCUT-Mix14 and (d) SCUT-Mix33, after being stored in the LC cells at RT (25-30 °C) for over a year. Syn-parallel alignment, cell thickness of SCUT-Mix14 is 4.4 μm, and cell thickness of SCUT-Mix33 is 7.9 μm. Scale bar: 100 μm. Notably, the two images were taken in the same LC cell, but due to the gradual, continuous changes in the texture, it was impossible to locate the exact region of the initial image based solely on the texture features. After being stored at RT for over a year, no significant phase separation was observed from the POM textures, demonstrating the excellent stability of the polar LC mixture materials.


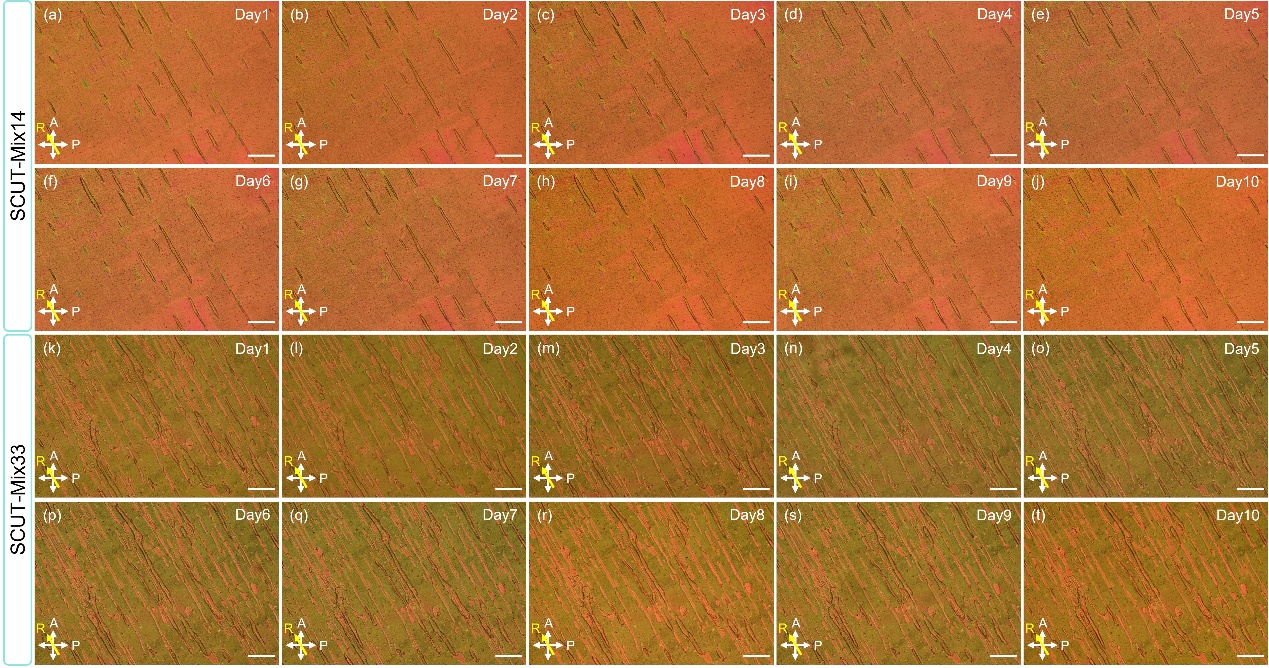


**Figure S22.** POM textures of (a-j) SCUT-Mix14 and (k-t) SCUT-Mix33 were recorded for the same region over a continuous 10-day period at RT (25-30 °C). Syn-parallel alignment, cell thickness of SCUT-Mix14 is 4.4 μm, and cell thickness of SCUT-Mix33 is 7.9 μm. Scale bar: 100 μm. The images shown here are from the same LC cell used in Figure S21, which had been stored at RT for over a year. Continuous observation over 10 days of LC cells stored at RT for over a year revealed almost no noticeable changes after the texture stabilized, demonstrating the excellent stability of the polar LC mixture materials.


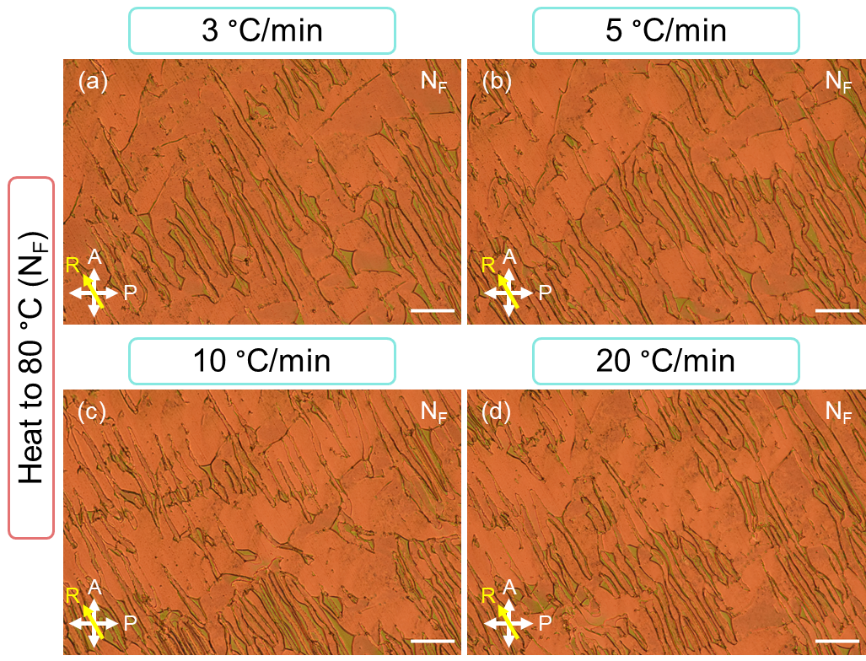


**Figure S23.** The POM texture of the N_F_ phase at room temperature of SCUT-Mix33, after being heated to the high-temperature N_F_ phase (80 °C) and cooled to RT (30 °C) at different rates. (a) 3 °C/min, (b) 5 °C/min, (c) 10 °C/min, (d) 20 °C/min. Syn-parallel alignment, cell thickness: 5.0 μm. Scale bar: 100 μm. The material was subjected to repeated heating and cooling cycles at different rates within the N_F_ phase temperature window, with no evidence of crystallization or phase separation, demonstrating excellent reproducibility.


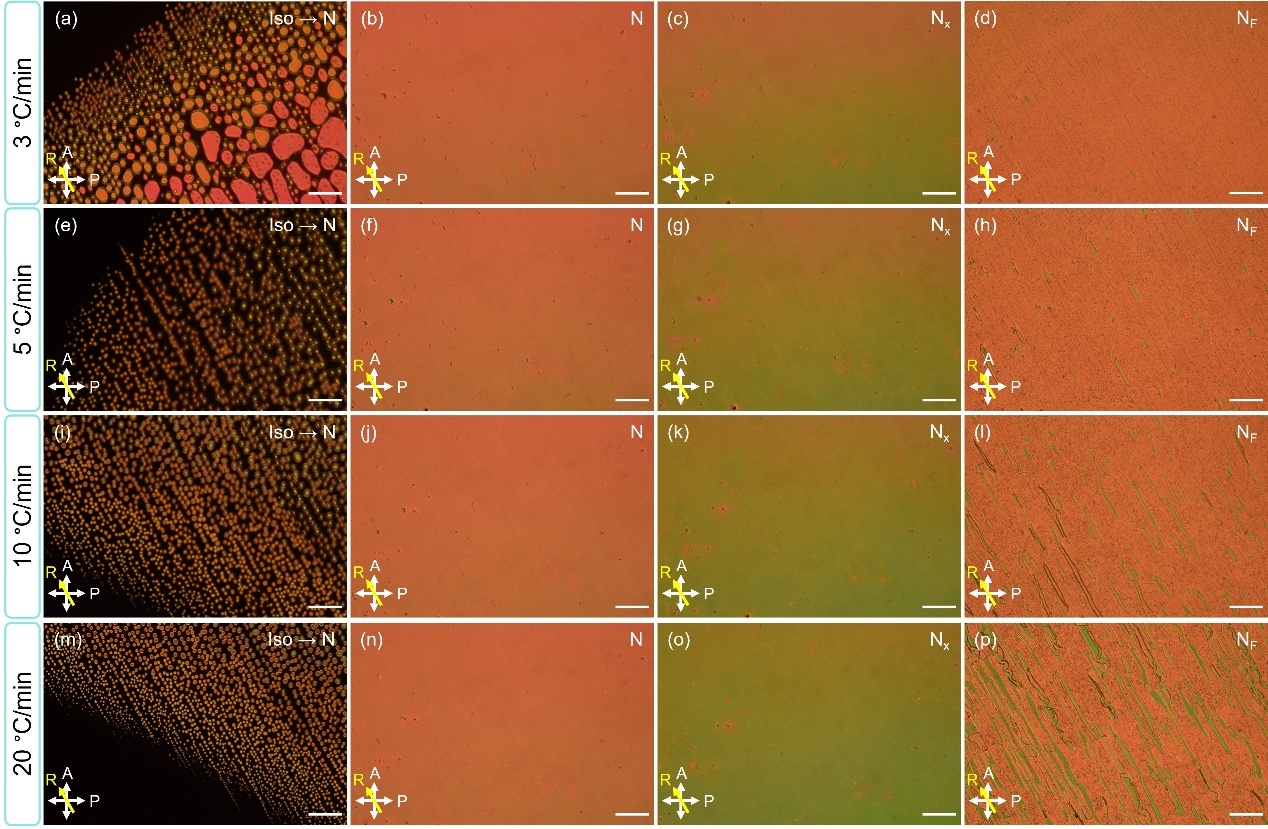


**Figure S24.** POM textures of SCUT-Mix14 during the cooling process at different cooling rates. (a-d) 3 °C/min: (a) 238.5 °C, phase transition from Iso to N phase; (b) 160 °C, N phase; (c) 150 °C, N_x_ phase; (d) 30 °C, N_F_ phase. (e-h) 5 °C/min: (e) 238 °C, phase transition from Iso to N phase; (f) 160 °C, N phase; (g) 150 °C, N_x_ phase; (h) 30 °C, N_F_ phase. (i-l) 10 °C/min: (i) 236 °C, phase transition from Iso to N phase; (j) 160 °C, N phase; (k) 150 °C, N_x_ phase; (l) 30 °C, N_F_ phase. (m-p) 20 °C/min: (m) 233.5 °C, phase transition from Iso to N phase; (n) 160 °C, N phase; (o) 150 °C, N_x_ phase; (p) 30 °C, N_F_ phase. Syn-parallel alignment, cell thickness: 5.0 μm. Scale bar: 100 μm. As the cooling rate increases, the phase transition temperature decreases, possibly due to supercooling phenomenon. Slower cooling rates result in fewer texture defects in the LC material, as the sufficiently slow phase transition promotes molecular packing and helps eliminate defects. Additionally, the defects already formed are given enough time to relax and be eliminated. During the cooling process at different cooling rates, the phase transition sequence remained consistent, and no phase separation was observed from the POM textures, demonstrating the excellent reproducibility of the polar LC mixture materials SCUT-Mix14.


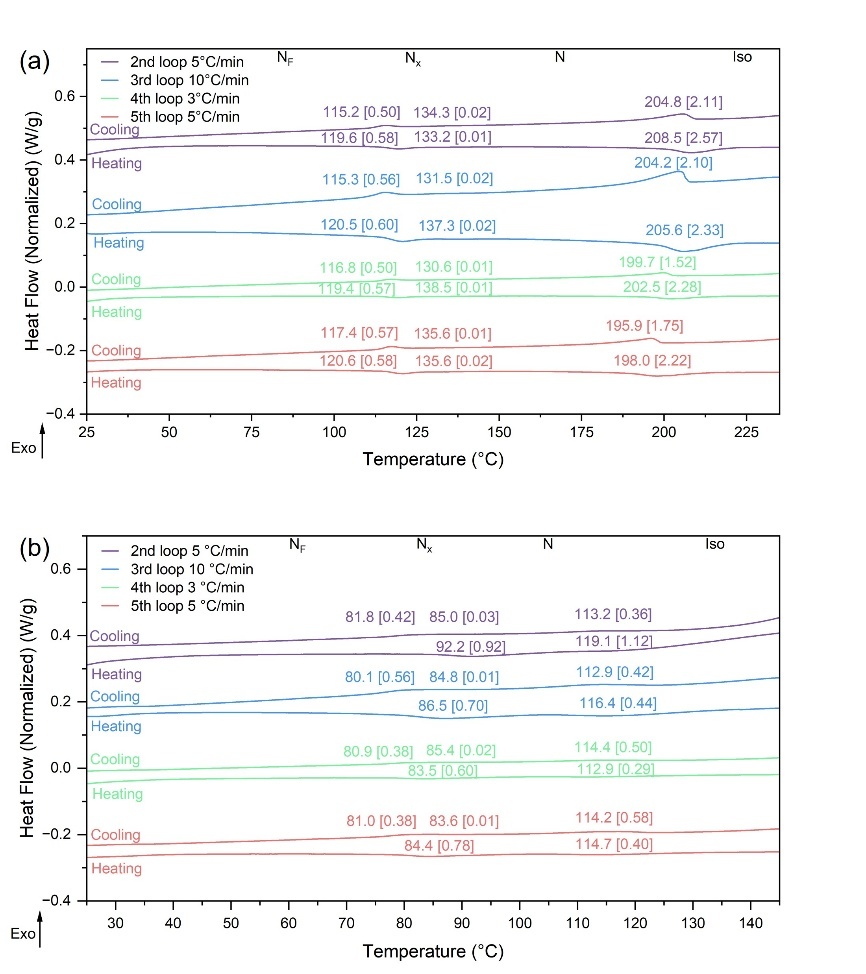


**Figure S25.** DSC profiles of (a) SCUT-Mix14 and (b) SCUT-Mix33 with different heating and cooling rate. The transition temperature and corresponding enthalpy [J g^−1^] of each phase are marked. During the DSC heating and cooling loops, conducted at different heating and cooling rates, the phase transition temperatures exhibited only slight variations within the error range, remaining stable. This demonstrates the excellent reproducibility of the polar LC mixture materials.


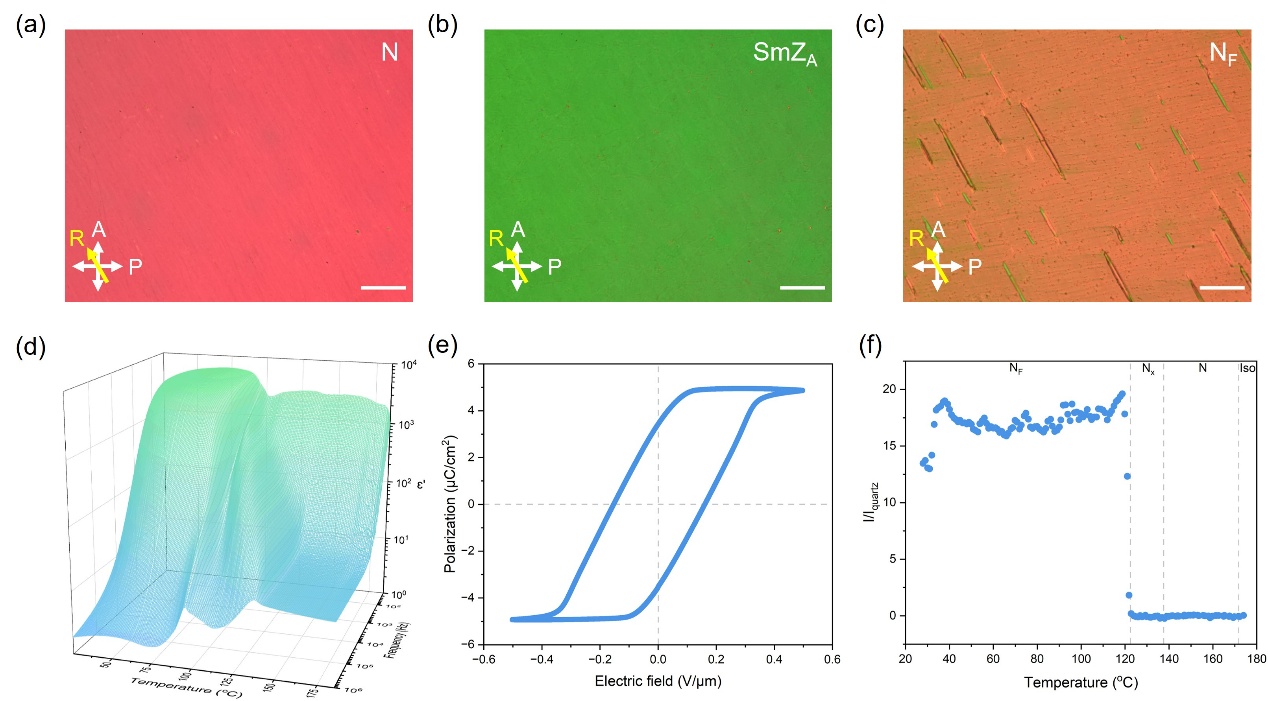


**Figure S26.** The phase behavior and electro-optic properties of SCUT-Mix27. (a-c) POM textures during the cooling process. (a) 150 °C, N phase; (b) 138 °C, N_x_ phase; (c) 30 °C, N_F_ phase. Syn-parallel alignment; cell thickness: 4.3 μm. (d) Temperature and frequency dependence of the dielectric constant. ITO natural cell; cell thickness: 20 μm. (e) The P-E hysteresis loop measured in the N_F_ phase (40 °C). ITO natural cell; cell thickness: 20 μm; Vp = 10 V, f = 200 Hz. (f) Temperature dependence of SHG intensity. Syn-parallel alignment EHC LC cell; cell thickness: 2.5 μm.


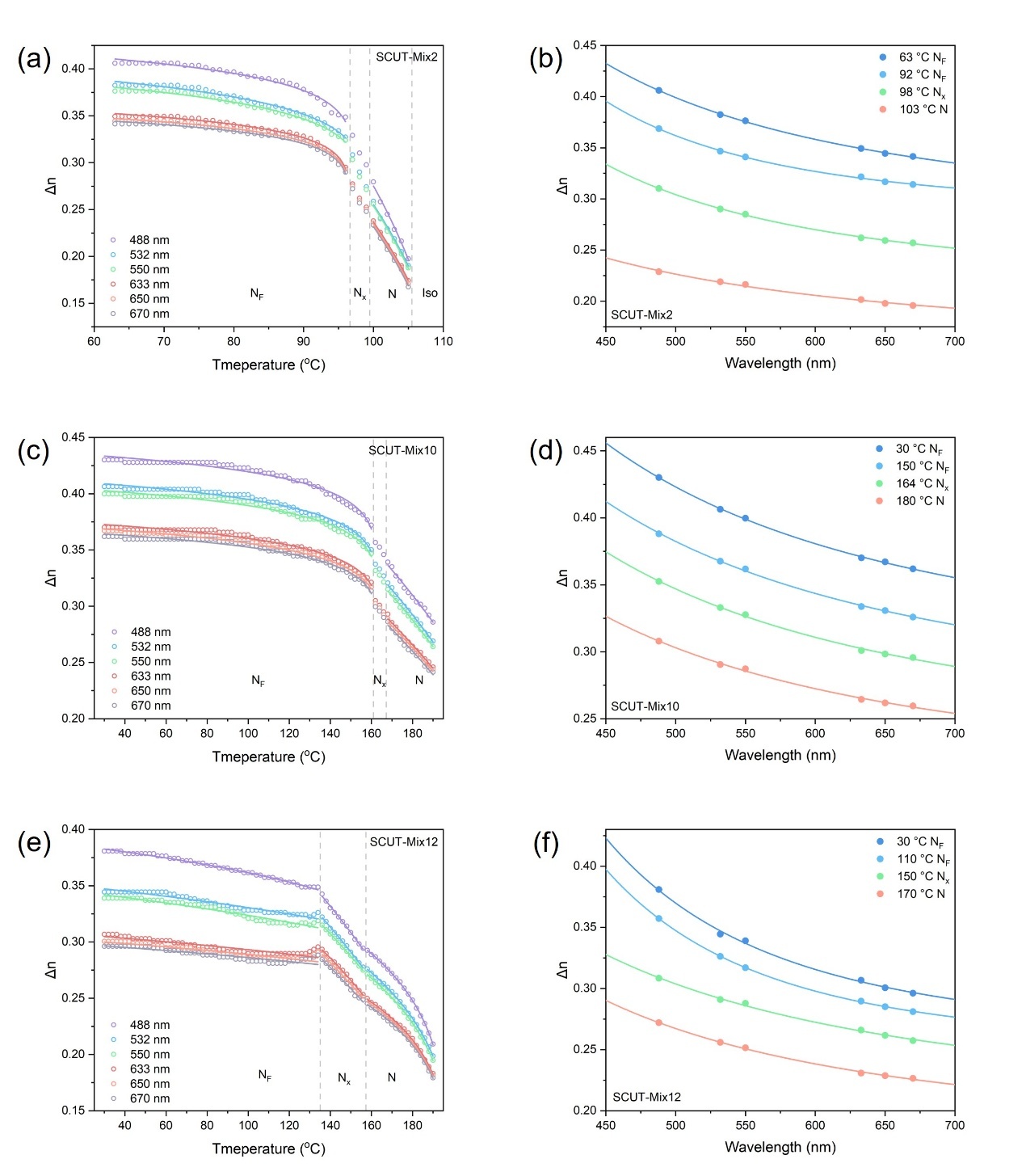


**Figure S27.** Birefringence and dispersion of polar LC mixture materials: (a-b) SCUT-Mix2, (c-d) SCUT-Mix10, and (e-f) SCUT-Mix12. (a, c, e) During cooling, temperature dependencies of birefringence at different wavelengths (488 nm, 532 nm, 550 nm, 633 nm, 650 nm, 670 nm). The curve in the figure is fitted by equation (1) in the main text. (b, d, f) Variation of birefringence with wavelength in different LC phases. The curve in the figure is fitted by formula (3) in the main text. Birefringence measurement using syn-parallel alignment EHC LC cell, with cell thicknesses of 2.0 μm for SCUT-Mix2, 2.0 μm for SCUT-Mix10, and 2.2 μm for SCUT-Mix12.


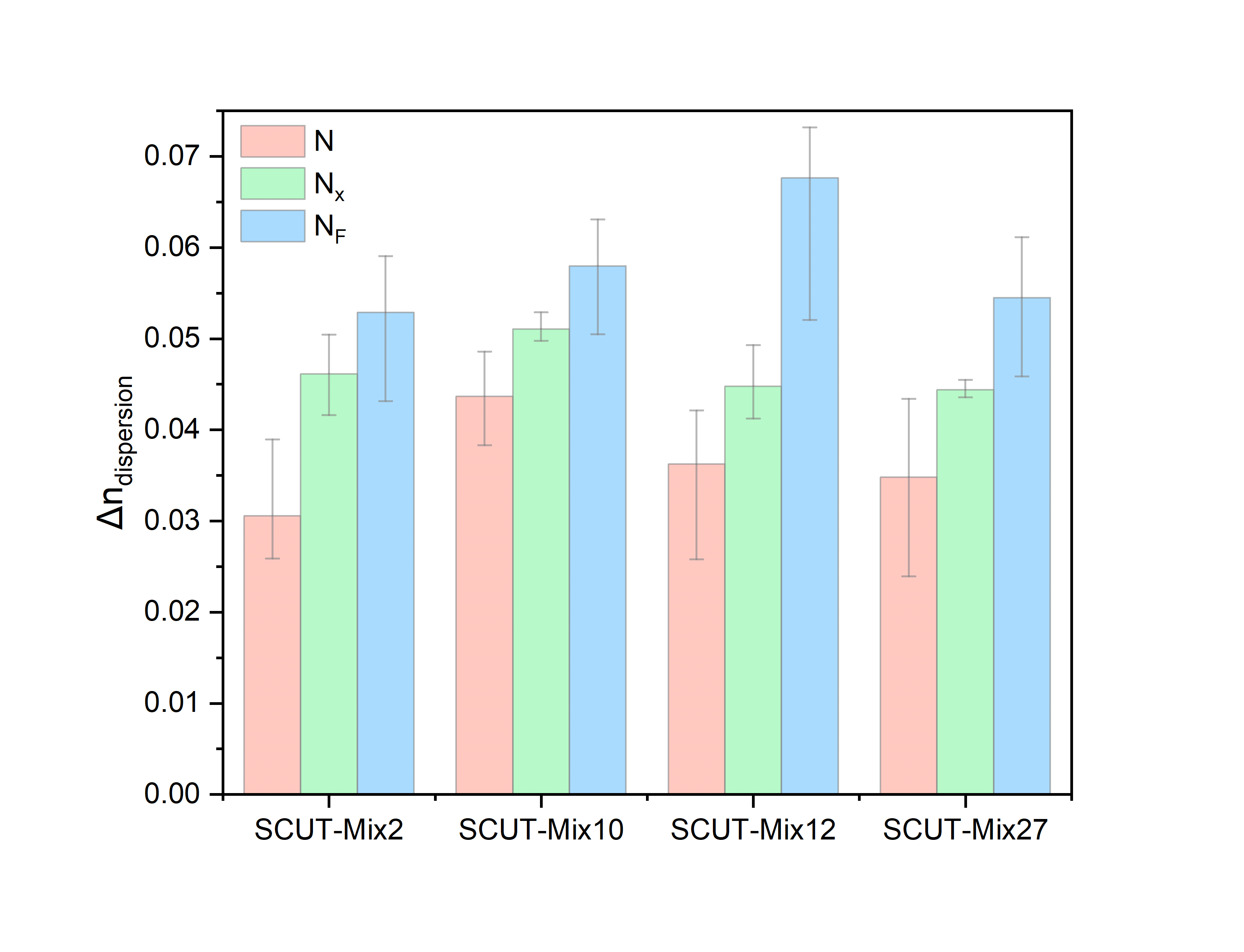


**Figure S28.** Calculated birefringence dispersion Δn_dispersion_ of each LC phase for various polar LC mixture materials based on equation (4). Δn_dispersion_ = Δn_short_ - Δn_long_, where Δn_short_ and Δn_long_ represent the calculated birefringence values at 488 nm and 632.8 nm from the fitted Cauchy dispersion formula, respectively.


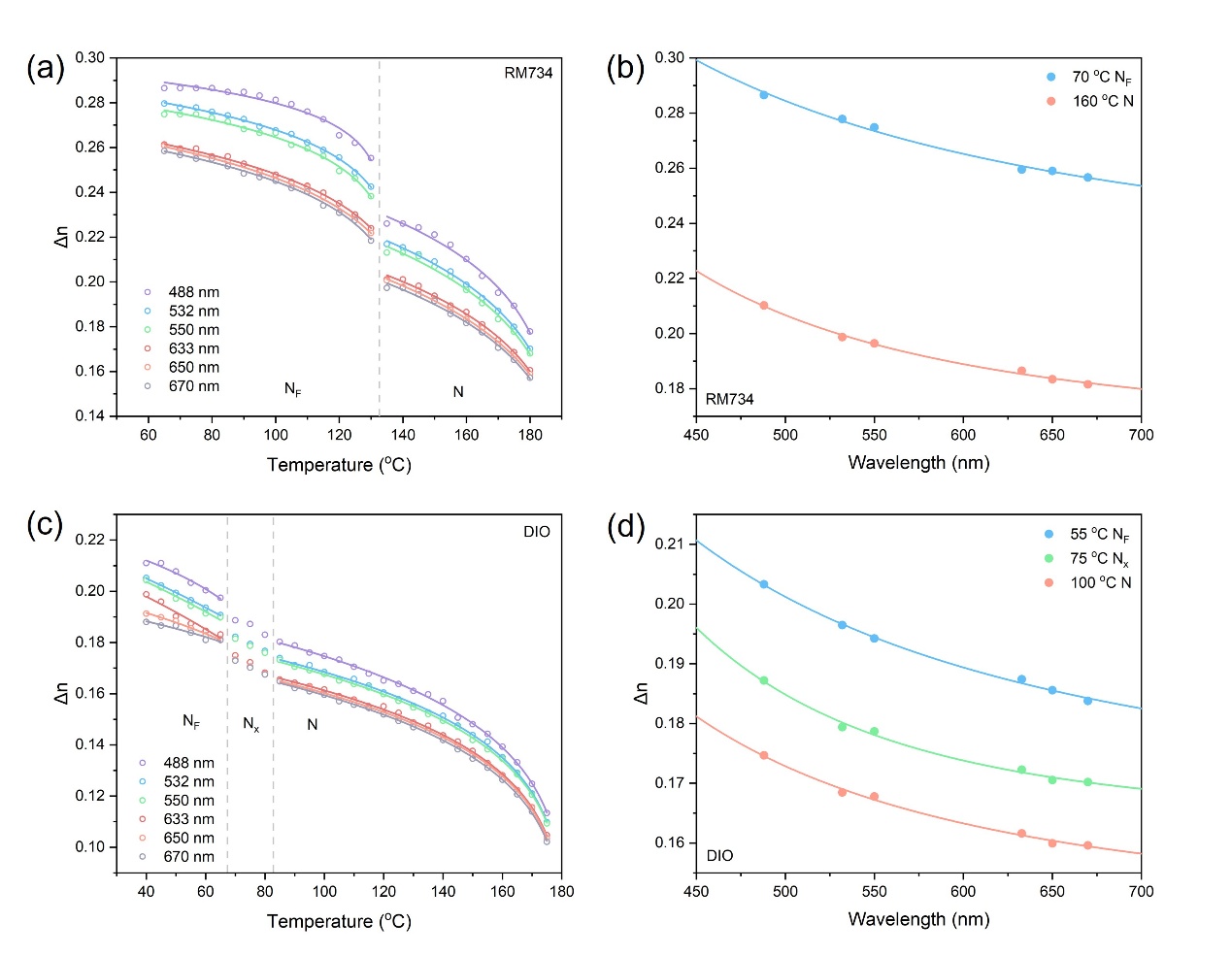


**Figure S29.** Birefringence and dispersion properties of RM734 and DIO. Temperature dependencies of (a) RM734 and (c) DIO molecules birefringence at different wavelengths (488 nm, 532 nm, 550 nm, 633 nm, 650 nm, 670 nm) during cooling. The curve in the figure is fitted by equation (1) in the main text. Variation of birefringence with wavelength in different LC phases of (b) RM734 and (d) DIO molecules. The curve in the figure is fitted by formula (3) in the main text. Birefringence measurement using syn-parallel alignment EHC LC cell, cell thickness: 2.5 μm.


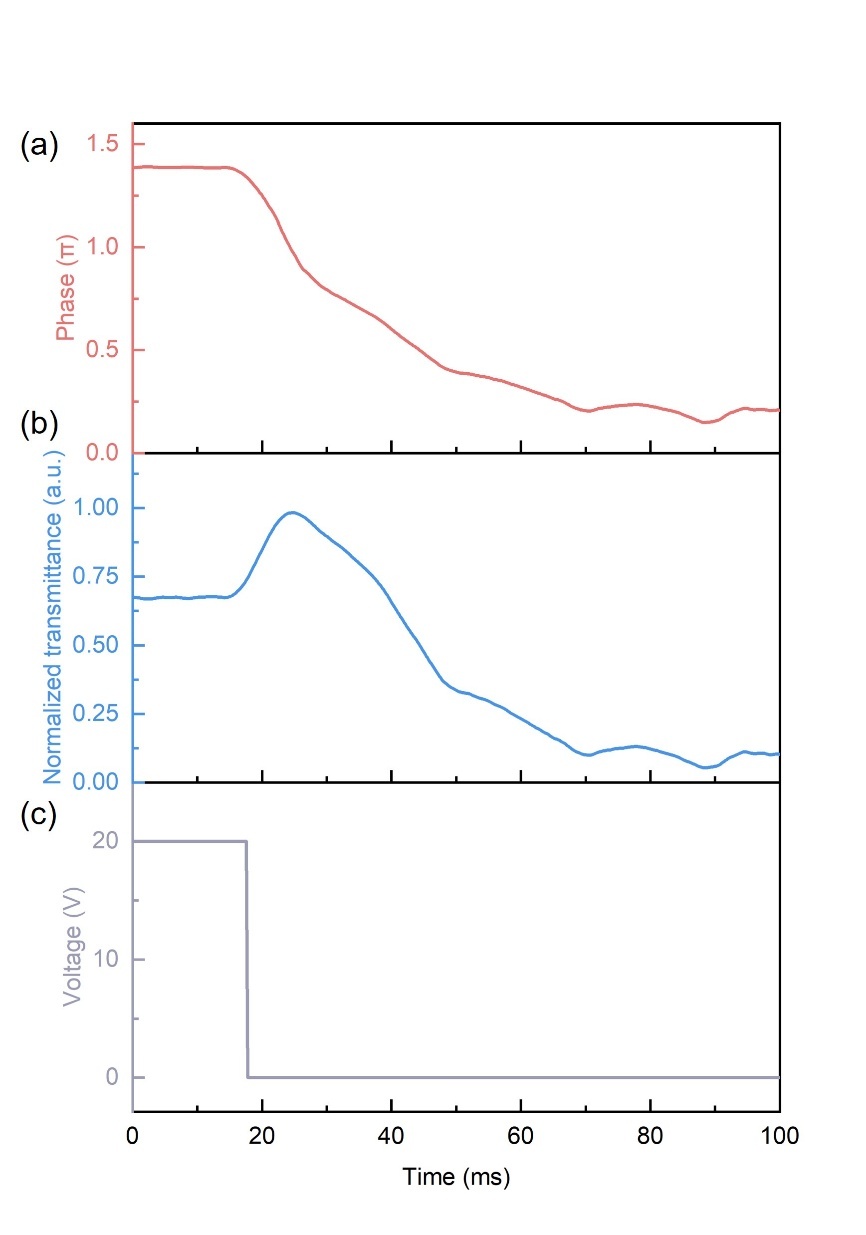


**Figure S30**. The in-plane switching (IPS) mode of the SCUT-Mix10 under the application of an electric field. After removing the applied voltage, the change in normalized transmittance was recorded and converted into the corresponding modulation phase retardation change. The phase retardation of the N_F_ phase of SCUT-Mix10 was measured in a syn-parallel aligned LC cell with comb-shaped ITO electrodes, where the alignment direction was parallel to the electrodes, i.e., perpendicular to the applied electric field. Temperature: 30 °C, N_F_ phase. Electrode gaps: 1 mm. Applied direct-current (DC) voltage: 20 V, corresponding electric field: 2 × 10^4^ V m^-1^. Cell thickness: 6.1 μm.

**Table S1.** Phase transition pathways and temperatures of each molecule in the rigid polar LC molecule library

| **Molecule** | **LC phases and corresponding phase transition temperatures (℃)^a)^** | | | | | |
| --- | --- | --- | --- | --- | --- | --- |
|  | **Melt^b)^** | **Iso - N** | **N_x_** | **N_F_** | **SmA_F_** | **Cry** |
| SCUT1a^c)^ | 183.3 | 219.5 | 195.9 | 187.3 | - | 158.0 |
| SCUT1b^c)^ | 113.9 | 72.4 | 67.0 | 37.3 | - | - |
| SCUT1c^c)^ | 163.5 | 207.3 | 150.6 | - | - | 137.4 |
| SCUT2a | 197 | - | - | - | - | 189 |
| SCUT2b | 128 | - | - | - | - | 89 |
| SCUT3a | 210 | 245 | - | - | - | 182 |
| SCUT3b | 159 | - | - | - | - | 74 |
| SCUT4a^c)^ | 106.6 | 181.1 | 146.4 | 135.4 | 105.7 | 68.4 |
| SCUT4b | 107 | 159 | - | - | - | 103 |
| SCUT5a | 118.5 | 280 | 202.4 | 184.7 | 120.3 | 62.3 |
| SCUT5b | 108.4 | 268 | 153.5 | 96.9 | 86.6 | 74.1 |
| SCUT5c | 142.1 | 158.6 | 89.2 | - | - | 81.8 |
| SCUT5d | 164 | 156 | - | - | - | 110 |
| SCUT6a | 130.7 | 204.5 | - | - | 151.4 | 117.3 |
| SCUT6b | 124 | 211 | **SmA**(168) | - | - | 102 |
| SCUT6c | 145 | 101 | - | - | - | 75 |
| SCUT6d | 165 | + | - | - | - | 97 |
| SCUT7a | 148.9 | 285 | 191.1 | 163.3 | - | 117.9 |
| SCUT7b | 145 | 282 | 150 | - | - | 112 |
| SCUT7c | 132 | 200 | - | - | - | 97 |
| SCUT7d | 140 | 178 | - | - | - | 48 |
| SCUT8a | 215 | >300 | - | - | - | 210 |
| SCUT8b | 182 | 304 | - | - | - | 147 |
| SCUT8c | 207 | 258 | - | - | - | 162 |
| **Sample** | **LC phases and corresponding phase transition temperatures (℃)^a)^** | | | | | |
|  | **Melt^b)^** | **Iso - N** | **N_x_** | **N_F_** | **SmA_F_** | **Cry** |
| SCUT8d | 169 | 230 | - | - | - | 140 |
| SCUT9a | 133 | 143 | - | - | - | 104 |
| SCUT9b | 115 | 145 | - | - | - | 84 |
| SCUT10a | 136 | 133 | - | - | - | 97 |
| SCUT10b | 114 | 80 | - | - | - | 30 |
| SCUT10c | 132 | - | - | - | - | 53 |
| SCUT10d | 100 | - | - | - | - | 71 |
| SCUT11a^c)^ | 82.8 | 89.5 | - | + | - | 73.5 |
| SCUT11b^c)^ | 72.5 | 93.3 | - | + | - | 59.5 |
| SCUT11c | 62 | - | - | - | - | 45 |
| SCUT12a | 168 | - | - | - | - | 158 |
| SCUT12b | 120 | - | - | - | - | 150 |
| SCUT13a | 100 | - | - | - | - | 81 |
| SCUT13b | 75 | - | - | - | - | 75 |
| SCUT14a | 205 | - | - | - | - | 186 |
| SCUT14b | 190 | - | - | - | - | 158 |
| SCUT15a | 95 | 63 | - | - | - | 58 |
| SCUT16a | 133 | - | - | - | - | 115 |
| SCUT16b | 157 | - | - | - | - | 125 |
| SCUT17a | 160 | 175 | - | - | - | 140 |
| SCUT17b | 155 | 160 | - | - | - | 124 |
| SCUT17c | 130 | 157 | - | - | - | 108 |
| SCUT17d | 127 | 145 | - | - | - | 109 |
| SCUT17e | 111 | 120 | - | - | - | 103 |
| SCUT17f | 121 | - | - | - | - | 113 |
| SCUT18a | 140 | 167 | - | - | - | 105 |
| **Sample** | **LC phases and corresponding phase transition temperatures (℃)^a)^** | | | | | |
|  | **Melt^b)^** | **Iso - N** | **N_x_** | **N_F_** | **SmA_F_** | **Cry** |
| SCUT18b | 135 | 160 | - | - | - | 91 |
| SCUT19a | 247 | 250 | - | - | - | 235 |
| SCUT19b | 239 | 245 | - | - | - | 228 |
| SCUT20a^c)^ | 85.5 | 104.8 | - | + | - | 74.4 |
| SCUT20b | 78 | 98 | - | - | - | 67 |
| SCUT21a | 145 | - | - | - | - | 110 |
| SCUT21b | 96 | - | - | - | - | 83 |
| SCUT22a^c)^ | 119.7 | 133.7 | - | - | - | 80.6 |
| SCUT22b^c)^ | 122.0 | 114.4 | 109.1 | 106.9 | - | 59.0 |
| SCUT22c^c)^ | 98.2 | 90.3 | 83.8 | 80.5 | - | 63.7 |
| SCUT22d | 78 | 78 | 67 | 60 | - | 58 |
| SCUT22e | 94 | - | - | - | - | - |
| SCUT22f | 67 | - | - | - | - | - |
| SCUT23a | 133 | - | - | 113 | - | 108 |
| SCUT23b | 128 | - | - | - | - | 103 |
| SCUT24a | 198 | 199 | - | - | - | 195 |
| SCUT24b | 192 | - | - | - | - | 193 |
| SCUT25a | 77 | - | - | - | - | 66 |

^a)^ The phase transition temperature in the table indicates the temperature at which the molecule changes from the previous LC phase to this phase during the cooling process. All samples were heated to the Iso phase and then cooled.

^b)^ The melting temperature of the molecule during the first heating process from the crystal.

^c)^ Phase transition temperature measured by DSC. Otherwise, it means measured under POM.

The “-” represents that the molecule does not present this stable LC phase above 30 ℃ during cooling.

The "+" represents that this molecule presents this metastable LC phase under supercooling conditions.

^d)^ During the cooling process, the SCUT15 molecule phase transitions from the non-polar N to the non-polar SmA phase at 168 ℃. After the polarization switching measurement, it was confirmed that it does not have ferroelectricity, so it is not specially marked in Figure 1 of the main text.

Note: Determining phase transition temperatures of LC materials may vary depending on the testing conditions. In LC cells, the orientational effect of the alignment layer enhances intermolecular dipole-dipole interactions, promoting the ordered arrangement of LC molecules. This effect may make phase transition easier, which results in an increase in the phase transition temperature compared to those (the same materials) measured by DSC. In contrast, during DSC measurements, where the LC material is placed in a metal crucible without any alignment influence, some LC phases with narrow temperature windows may hard to be detect, resulting in the absence of corresponding exothermic or endothermic peaks. Therefore, the lack of an exothermic or endothermic peak for a specific phase transition in the DSC data does not imply the non-existence of that phase. A more comprehensive analysis should integrate DSC results with POM observations, as demonstrated by the N_F_-SmA_F_ transition in SCUT5b, the N_x_-N_F_ transition in SCUT22d, and the Iso-N_F_ transition in SCUT23a.

**Table S2.** The temperature-dependent birefringence fitting parameters for each molecule fitted by equation (1) in the main text

| **Molecule** | | | **SCUT1a** | **SCUT4a** | **SCUT5a** | **SCUT5b** | **SCUT6a** | **SCUT7a** | **SCUT22b** | **SCUT22c** | |
| --- | --- | --- | --- | --- | --- | --- | --- | --- | --- | --- | --- |
| 488 nm | N | Δn_0_ | 0.26 | 0.37 | 0.41 | 0.40 | 0.30 | 0.43 | (0.34) | 0.50 |  |
|  |  | β | 0.09 | 0.23 | 0.12 | 0.20 | 0.11 | 0.13 | (0.10) | 0.27 |  |
|  | N_x_ | Δn_0_ | 0.33 | (0.44) | (0.36) | 0.39 | - | 0.53 | (0.54) | (0.42) |  |
|  |  | β | 0.14 | (0.30) | (0.00) | 0.09 | - | 0.30 | (0.13) | (0.16) |  |
|  | N_F_ | Δn_0_ | 0.28 | 0.31 | 0.40 | (0.39) | - | 0.43 | 0.45 | 0.40 |  |
|  |  | β | 0.01 | 0.02 | 0.02 | (0.01) | - | 0.02 | 0.09 | 0.06 |  |
|  | SmA_F_ | Δn_0_ | - | 0.32 | 0.40 | 0.39 | 0.30 | - | - | - |  |
|  |  | β | - | 0.00 | 0.00 | 0.00 | 0.02 | - | - | - |  |
| 532 nm | N | Δn_0_ | 0.25 | 0.34 | 0.40 | 0.37 | 0.28 | 0.43 | (0.34) | 0.45 |  |
|  |  | β | 0.09 | 0.20 | 0.15 | 0.17 | 0.11 | 0.17 | (0.11) | 0.25 |  |
|  | N_x_ | Δn_0_ | 0.32 | (0.43) | (0.34) | 0.38 | - | 0.48 | (0.55) | (0.32) |  |
|  |  | β | 0.12 | (0.30) | (0.00) | 0.13 | - | 0.26 | (0.15) | (0.06) |  |
|  | N_F_ | Δn_0_ | 0.28 | 0.30 | 0.38 | (0.36) | - | 0.40 | 0.42 | 0.40 |  |
|  |  | β | 0.01 | 0.02 | 0.03 | (0.00) | - | 0.01 | 0.08 | 0.11 |  |
|  | SmA_F_ | Δn_0_ | - | 0.31 | 0.37 | 0.36 | 0.28 | - | - | - |  |
|  |  | β | - | (0.00) | (0.00) | 0.00 | 0.02 | - | - | - |  |
| 550 nm | N | Δn_0_ | 0.24 | 0.34 | 0.40 | 0.37 | 0.28 | 0.41 | (0.33) | 0.45 |  |
|  |  | β | 0.08 | 0.25 | 0.16 | 0.19 | 0.11 | 0.14 | (0.11) | 0.26 |  |
|  | N_x_ | Δn_0_ | 0.33 | (0.41) | (0.34) | 0.38 | - | 0.47 | (0.52) | (0.48) |  |
|  |  | β | 0.14 | (0.30) | (0.00) | 0.17 | - | 0.25 | (0.14) | (0.30) |  |
|  | N_F_ | Δn_0_ | 0.27 | 0.30 | 0.37 | (0.36) | - | 0.40 | 0.42 | 0.40 |  |
|  |  | β | 0.01 | 0.02 | 0.02 | (0.00) | - | 0.02 | 0.09 | 0.12 |  |
|  | SmA_F_ | Δn_0_ | - | 0.30 | 0.36 | 0.36 | 0.28 | - | - | - |  |
|  |  | β | - | (0.00) | 0.00 | 0.00 | 0.02 | - | - | - |  |

| **Molecule** | | | **SCUT1a** | **SCUT4a** | **SCUT5a** | **SCUT5b** | **SCUT6a** | **SCUT7a** | **SCUT22b** | **SCUT22c** | |
| --- | --- | --- | --- | --- | --- | --- | --- | --- | --- | --- | --- |
| 632.8 nm | N | Δn_0_ | 0.23 | 0.31 | 0.36 | 0.35 | 0.26 | 0.37 | (0.32) | 0.39 |  |
|  |  | β | 0.08 | 0.20 | 0.15 | 0.19 | 0.10 | 0.12 | (0.12) | 0.23 |  |
|  | N_x_ | Δn_0_ | 0.28 | (0.38) | (0.31) | 0.34 | - | 0.41 | (0.47) | (0.38) |  |
|  |  | β | 0.11 | (0.30) | (0.00) | 0.09 | - | 0.12 | (0.14) | (0.17) |  |
|  | N_F_ | Δn_0_ | 0.27 | 0.32 | 0.35 | (0.34) | - | 0.37 | 0.38 | 0.40 |  |
|  |  | β | 0.02 | 0.08 | 0.02 | (0.00) | - | 0.01 | 0.08 | 0.24 |  |
|  | SmA_F_ | Δn_0_ | - | 0.28 | 0.34 | 0.33 | 0.26 | - | - | - |  |
|  |  | β | - | 0.00 | 0.00 | (0.00) | 0.01 | - | - | - |  |
| 650 nm | N | Δn_0_ | 0.23 | 0.33 | 0.35 | 0.35 | 0.26 | 0.37 | (0.32) | 0.38 |  |
|  |  | β | 0.08 | 0.27 | 0.13 | 0.20 | 0.11 | 0.14 | (0.12) | 0.23 |  |
|  | N_x_ | Δn_0_ | 0.28 | (0.40) | (0.31) | 0.33 | - | 0.43 | (0.46) | (0.43) |  |
|  |  | β | 0.11 | (0.30) | (0.00) | 0.07 | - | 0.23 | (0.13) | (0.30) |  |
|  | N_F_ | Δn_0_ | 0.26 | 0.30 | 0.34 | (0.33) | - | 0.37 | 0.38 | 0.35 |  |
|  |  | β | 0.01 | 0.05 | 0.02 | (0.00) | - | 0.02 | 0.08 | 0.08 |  |
|  | SmA_F_ | Δn_0_ | - | 0.28 | 0.33 | 0.33 | 0.25 | - | - | - |  |
|  |  | β | - | (0.00) | 0.00 | 0.00 | 0.01 | - | - | - |  |
| 670 nm | N | Δn_0_ | 0.22 | 0.32 | 0.35 | 0.34 | 0.26 | 0.37 | (0.32) | 0.38 |  |
|  |  | β | 0.08 | 0.23 | 0.14 | 0.21 | 0.11 | 0.15 | (0.13) | 0.23 |  |
|  | N_x_ | Δn_0_ | 0.28 | (0.36) | (0.30) | 0.32 | - | 0.44 | (0.45) | (0.30) |  |
|  |  | β | 0.10 | (0.30) | (0.01) | 0.05 | - | 0.24 | (0.14) | (0.07) |  |
|  | N_F_ | Δn_0_ | 0.26 | 0.29 | 0.34 | (0.33) | - | 0.36 | 0.37 | 0.34 |  |
|  |  | β | 0.02 | 0.03 | 0.02 | (0.01) | - | 0.02 | 0.08 | 0.09 |  |
|  | SmA_F_ | Δn_0_ | - | 0.28 | 0.33 | 0.33 | 0.25 | - | - | - |  |
|  |  | β | - | (0.00) | (0.00) | (0.00) | 0.01 | - | - | - |  |

The “-” represents that the molecule does not present this stable LC phase during cooling.

The data in brackets “()” indicate that the temperature window of the LC phase of this molecule is narrow, and there may be certain errors in the fitting data.

**Table S3.** Average birefringence dispersion Δn_dispersion_ and its corresponding error of each phase of polar LC molecules in the molecular library

| **Molecule** | | **SCUT1a** | **SCUT4a** | **SCUT5a** | **SCUT5b** | **SCUT6a** | **SCUT7a** | **SCUT22b** | **SCUT22c** |
| --- | --- | --- | --- | --- | --- | --- | --- | --- | --- |
| N | Δn_dispersion_ | 0.021 | 0.025 | 0.049 | 0.043 | 0.029 | 0.048 | 0.032 | 0.030 |
|  | Error+ | 0.002 | 0.004 | 0.001 | 0.001 | 0.005 | 0.002 | 0.001 | 0.007 |
|  | Error- | -0.002 | -0.005 | -0.001 | -0.001 | -0.010 | -0.001 | -0.001 | 0.006 |
| N_x_ | Δn_dispersion_ | 0.026 | 0.030 | 0.047 | 0.048 | - | 0.053 | 0.042 | 0.040 |
|  | Error+ | 0.003 | 0.001 | ~0 | 0.006 | - | 0.004 | 0.007 | 0.005 |
|  | Error- | -0.002 | -0.001 | ~0 | -0.003 | - | -0.003 | -0.005 | -0.002 |
| N_F_ | Δn_dispersion_ | 0.023 | 0.024 | 0.053 | 0.052 | - | 0.059 | 0.057 | 0.049 |
|  | Error+ | 0.002 | 0.006 | 0.002 | 0.001 | - | 0.004 | 0.005 | 0.003 |
|  | Error- | -0.001 | -0.007 | -0.002 | ~0 | - | -0.005 | -0.008 | 0.004 |
| SmA_F_ | Δn_dispersion_ | - | 0.034 | 0.052 | 0.051 | 0.036 | - | - | - |
|  | Error+ | - | ~0 | ~0 | ~0 | 0.002 | - | - | - |
|  | Error- | - | ~0 | ~0 | -0.002 | -0.005 | - | - | - |

The “-” represents that the molecule does not present this stable LC phase during cooling.

**Table S4.** Phase transition pathways and temperatures of polar LC mixture materials obtained by mixing molecules from the rigid polar LC molecule library

| **Material** | **Component** | **Ratio (wt%)** | **LC phases and corresponding phase transition temperatures (℃)^a)^** | | | | | |
| --- | --- | --- | --- | --- | --- | --- | --- | --- |
|  |  |  | **Melt^b)^** | **Iso - N** | **N_x_** | **N_F_** | **SmA_F_** | **Cry** |
| SCUT-Mix1 | SCUT22b, SCUT22c | 7:3 | 116 | 109 | 105 | 101 | - | 53 |
| SCUT-Mix2 | SCUT22b, SCUT22c | 1:1 | 110 | 105 | 99 | 96 | - | 57 |
| SCUT-Mix3 | SCUT22b, SCUT22c | 3:7 | 100 | 98 | 89 | 86 | - | 55 |
| SCUT-Mix4 | SCUT22b, SCUT22d | 1:1 | 100 | 98 | 86 | 83 | - | 67 |
| SCUT-Mix5 | SCUT22c, SCUT22d | 1:1 | 94 | 86 | 72 | 70 | - | 68 |
| SCUT-Mix6 | SCUT22b, SCUT22f | 1:1 | 62 | - | - | 57 | - | 26 |
| SCUT-Mix7 | SCUT22c, SCUT22f | 1:1 | 56 | - | - | 40 | - | 27 |
| SCUT-Mix8 | SCUT23a, SCUT24a | 1:1 | 114 | 108 | - | - | - | 80 |
| SCUT-Mix9 | SCUT5a, SCUT22b | 7:3 | 77 | 237 | 177 | 163 | 58 | 31 |
| SCUT-Mix10 | SCUT5a, SCUT22b | 1:1 | 78 | 205 | 161 | 148 | - | 26 |
| SCUT-Mix11 | SCUT5a, SCUT22b | 3:7 | 92 | 170 | 143 | 136 | - | 30 |
| SCUT-Mix12 | SCUT5a, SCUT22c | 1:1 | 66 | 193 | 131 | 126 | - | 26 |
| SCUT-Mix13 | SCUT5a, SCUT22c | 1:1 | 115 | 203 | 163 | 147 | - | 48 |
| SCUT-Mix14 | SCUT5a, SCUT5c | 1:1 | N_F_ | 229 | 148 | 130 | - | - |
| SCUT-Mix15 | SCUT6a, SCUT6b | 7:3 | 125 | 213 | - | - | 148 | 100 |
| SCUT-Mix16 | SCUT6a, SCUT6b | 1:1 | 124 | 216 | - | - | 160 | 85 |
| SCUT-Mix17 | SCUT6a, SCUT6b | 3:7 | 127 | 212 | - | - | 161 | 89 |
| SCUT-Mix18 | SCUT9a, SCUT9b | 1:1 | 115 | 146 | - | - | - | 84 |
| SCUT-Mix19 | SCUT22a, SCUT22b, SCUT22c | 1:1:1 | 110 | - | 114 | 112 | 110 | 59 |
| SCUT-Mix20 | SCUT22b, SCUT22c, SCUT22d | 1:1:1 | 100 | 96 | - | 87 | - | 71 |
| SCUT-Mix21 | SCUT22b, SCUT22c, SCUT22f | 9:9:2 | 100 | - | - | 90 | 81 | 45 |
| SCUT-Mix22 | SCUT22b, SCUT22c, SCUT22f | 2:2:1 | 95 | - | - | 81 | - | 42 |
| SCUT-Mix23 | SCUT22b, SCUT22c, SCUT22f | 7:7:6 | 85 | - | - | 70 | - | 30 |
| SCUT-Mix24 | SCUT22b, SCUT22c, SCUT22f | 3:3:4 | 74 | - | - | 52 | 31 | 26 |
| SCUT-Mix25 | SCUT22b, SCUT22c, SCUT22f | 1:1:2 | 73 | - | - | 45.6 | - | 28 |
| SCUT-Mix26 | SCUT22b, SCUT22c, SCUT22f | 1:1:3 | 58 | - | - | 33 | 30 | - |
| SCUT-Mix27 | SCUT5a, SCUT22b, SCUT22c | 1:1:1 | 80 | 172 | 138 | 133 | - | 28 |
| SCUT-Mix28 | SCUT5a, SCUT22b, SCUT22d | 1:1:1 | 77 | 167 | 132 | 122 | - | 43 |
| SCUT-Mix29 | SCUT5a, SCUT22c, SCUT22d | 1:1:1 | 74 | 159 | 131 | 115 | - | 44 |
| SCUT-Mix30 | SCUT22a, SCUT22b, SCUT22c, SCUT22d | 1:1:1:1 | 107 | 108 | 103 | 101 | - | 68 |
| SCUT-Mix31 | SCUT5a, SCUT22a, SCUT22b, SCUT22f | 3:3:3:1 | 75 | 150 | 125 | 118 | - | 28 |
| SCUT-Mix32 | SCUT5a, SCUT22b, SCUT22c, SCUT22f | 1:1:1:1 | 65 | 122 | - | 101 | - | 25 |
| SCUT-Mix33 | SCUT5a, SCUT5c, SCUT22c, SCUT22f | 1:1:1:1 | N_F_ | 136 | 103 | 100 | - | - |
| SCUT-Mix34 | SCUT5a, SCUT5c, SCUT22b, SCUT22c | 1:1:1:1 | SmA_F_ | 165 | 131 | 125 | 38 | - |

Phase transition temperature measured under POM.

The “-” represents that the molecule does not present this stable LC phase above 25 ℃ during cooling.

^a)^ The phase transition temperature in the table indicates the temperature at which the molecule changes from the previous LC phase to this phase during the cooling process. All samples were heated to the Iso phase and then cooled.

^b)^ The melting temperature of the molecule during the first heating process from the crystal. If the mixture material does not crystallize at room temperature, the abbreviation of the corresponding LC phase at room temperature is used here to mark.

**Table S5.** The temperature-dependent birefringence fitting parameters for polar LC mixture materials fitted by equation (1) in the main text

| **Material** | | | **SCUT-Mix2** | **SCUT-Mix10** | **SCUT-Mix12** | **SCUT-Mix27** |
| --- | --- | --- | --- | --- | --- | --- |
| 488 nm | N | Δn_0_ | 0.90 | 0.60 | 0.41 | 0.46 |
|  |  | β | 0.50 | 0.50 | 0.20 | 0.26 |
|  | N_x_ | Δn_0_ | (0.45) | (0.38) | 0.57 | 0.39 |
|  |  | β | (0.09) | (0.02) | 0.50 | 0.08 |
|  | N_F_ | Δn_0_ | 0.44 | 0.44 | 0.39 | 0.43 |
|  |  | β | 0.06 | 0.04 | 0.11 | 0.09 |
| 532 nm | N | Δn_0_ | 0.81 | 0.57 | 0.40 | 0.43 |
|  |  | β | 0.50 | 0.50 | 0.24 | 0.25 |
|  | N_x_ | Δn_0_ | (0.44) | (0.36) | 0.54 | 0.51 |
|  |  | β | (0.10) | (0.02) | 0.50 | 0.50 |
|  | N_F_ | Δn_0_ | 0.41 | 0.41 | 0.35 | 0.40 |
|  |  | β | 0.07 | 0.05 | 0.16 | 0.08 |
| 550 nm | N | Δn_0_ | 0.81 | 0.56 | 0.39 | 0.42 |
|  |  | β | 0.50 | 0.50 | 0.24 | 0.25 |
|  | N_x_ | Δn_0_ | (0.43) | (0.35) | 0.51 | 0.52 |
|  |  | β | (0.10) | (0.02) | 0.48 | 0.50 |
|  | N_F_ | Δn_0_ | 0.41 | 0.41 | 0.35 | 0.40 |
|  |  | β | 0.07 | 0.05 | 0.18 | 0.08 |
| 632.8 nm | N | Δn_0_ | 0.75 | 0.51 | 0.34 | 0.39 |
|  |  | β | 0.50 | 0.50 | 0.20 | 0.25 |
|  | N_x_ | Δn_0_ | (0.37) | (0.33) | 0.45 | 0.32 |
|  |  | β | (0.08) | (0.02) | 0.35 | 0.06 |
|  | N_F_ | Δn_0_ | 0.37 | 0.38 | 0.31 | 0.37 |
|  |  | β | 0.05 | 0.05 | 0.13 | 0.08 |

| **Material** | | | **SCUT-Mix2** | **SCUT-Mix10** | **SCUT-Mix12** | **SCUT-Mix27** |
| --- | --- | --- | --- | --- | --- | --- |
| 650 nm | N | Δn_0_ | 0.75 | 0.51 | 0.34 | 0.39 |
|  |  | β | 0.50 | 0.50 | 0.20 | 0.25 |
|  | N_x_ | Δn_0_ | (0.36) | (0.32) | 0.48 | 0.31 |
|  |  | β | (0.08) | (0.02) | 0.50 | 0.04 |
|  | N_F_ | Δn_0_ | 0.36 | 0.37 | 0.30 | 0.36 |
|  |  | β | 0.05 | 0.05 | 0.12 | 0.08 |
| 670 nm | N | Δn_0_ | 0.75 | 0.51 | 0.34 | 0.38 |
|  |  | β | 0.50 | 0.50 | 0.21 | 0.25 |
|  | N_x_ | Δn_0_ | (0.36) | (0.32) | 0.47 | 0.30 |
|  |  | β | (0.08) | (0.02) | 0.50 | 0.03 |
|  | N_F_ | Δn_0_ | 0.36 | 0.37 | 0.30 | 0.36 |
|  |  | β | 0.04 | 0.04 | 0.12 | 0.08 |

The data in brackets “()” indicate that the temperature window of the LC phase of this molecule is narrow, and there may be certain errors in the fitting data.

**Table S6.** Average birefringence dispersion Δn_dispersion_ and its corresponding error of each phase of polar LC mixture materials

| **Material** | | **SCUT-Mix2** | **SCUT-Mix10** | **SCUT-Mix12** | **SCUT-Mix27** |
| --- | --- | --- | --- | --- | --- |
| N | Δn_dispersion_ | 0.031 | 0.044 | 0.036 | 0.035 |
|  | Error+ | 0.008 | 0.005 | 0.006 | 0.009 |
|  | Error- | -0.005 | -0.005 | -0.010 | -0.011 |
| N_x_ | Δn_dispersion_ | 0.046 | 0.051 | 0.045 | 0.044 |
|  | Error+ | 0.004 | 0.002 | 0.004 | 0.001 |
|  | Error- | -0.005 | -0.001 | -0.004 | -0.001 |
| N_F_ | Δn_dispersion_ | 0.053 | 0.058 | 0.068 | 0.055 |
|  | Error+ | 0.006 | 0.005 | 0.006 | 0.007 |
|  | Error- | -0.010 | -0.008 | -0.016 | -0.009 |

**Synthesis**

**General Suzuki cross-coupling method:** The bromobenzene (1 eq), phenylboronic acid or 4,4,5,5-tetramethyl-2-phenyl-1,3,2-dioxaborolane (1.1 eq), potassium phosphate (K_3_PO_4_, 3 eq), dicyclohexyl(2',6'-dimethoxy-[1,1'-biphenyl]-2-yl)phosphine (SPhos, 0.08 eq), tris(dibenzylideneacetone)dipalladium(0) (Pd_2_(dba)_3_, 0.04 eq) were added to a stirred solution of a mixture of Vol (toluene)/ Vol (H_2_O) =7/3 in a Schlenk tube. Use liquid nitrogen to freeze the solvent, degassed it 3 times, and refill it with nitrogen atmosphere. Then the mixture solution was heated to 80 - 100 ^o^C overnight (12 - 16h). The mixture was cooled to room temperature and extracted with EA. The organic phase was washed with water and then dried with anhydrous MgSO_4_. After removing the solvent by evaporation under reduced pressure, the residual was dry-loaded onto a silica gel column for purification using EA/PE as eluent and dried in a vacuum oven. General yield 60 - 85%.

**General Sonogashira coupling method:** The bromobenzene (1 eq), phenyl acetylene or trimethylsilylacetylene (TMSA) (1.1 eq), triethylamine (Et_3_N, 3 eq), bis(triphenylphosphine)palladium(II) dichloride (Pd(PPh_3_)_2_Cl_2_, 0.02 eq), triphenylphosphine (PPh_3_, 0.02 eq), Copper(I) iodide (CuI, 0.01 eq) were added to a stirred solution of toluene in a Schlenk tube. Use liquid nitrogen to freeze the solvent, degassed it 3 times, and refill it with nitrogen atmosphere. Then the mixture solution was heated to reflux (90 - 110 ^o^C) overnight (12 - 16h). The mixture was cooled to room temperature and extracted with EA. The organic phase was washed with water and then dried with anhydrous MgSO_4_. After removing the solvent by evaporation under reduced pressure, the residual was dry-loaded onto a silica gel column for purification using EA/PE as eluent and dried in a vacuum oven. General yield 60 - 80%.

**Synthesis scheme of molecules**

**Scheme S1**. Synthesis scheme of precursors A.

**Scheme S2**. Synthesis scheme of precursors B.

**Scheme S3**. Synthesis scheme of molecules in the molecular library of rigid polar LC materials.

Reagents and conditions: (ⅰ) p-TsOH, BHT, MePh (130 °C). (ⅱ) a) n-BuLi, I_2_, THF (-78 °C); b) Na_2_S_2_O_3_. (ⅲ) a) TMSA, Et_3_N, Pd(PPh_3_)_2_Cl_2_, PPh_3_, CuI, MePh (90 °C); b) TBAF, THF (0 ^o^C). (iv) a) n-BuLi, CF_2_Br_2_, THF (-78 °C). (v) K_2_CO_3_, THF (60 ^o^C). (vi) NaH, THF (0 ^o^C). (vii) a) TosCl, THF (4 - 6 ^o^C), H_2_O, HCl; b) K_2_CO_3_, THF (80 ^o^C). (viii) (Bpin)_2_, K_3_PO_4_, SPhos, Pd_2_(dba)_3_, MePh/H_2_O (100 ^o^C). (ix) CH_3_COOK, PdCl_2_(dppf), MePh (100 ^o^C). (x) Et_3_N, Pd(PPh_3_)_2_Cl_2_, PPh_3_, CuI, MePh (100 °C). (xi) a) 1,3-dimercaptopropane, TfOH, MePh/isooctane (50 ^o^C); b) MTBE (0 ^o^C); c) Et_3_N, Et_3_N·3HF, Br_2_, DCM (-80 ^o^C, 0 ^o^C), NaOH. (xii) K_3_PO_4_, SPhos, Pd_2_(dba)_3_, MePh (110 ^o^C). (xiii) K_2_CO_3_, TBAB (75 °C). (xiv) DCC, DMAP, DCM (25 °C).

**^1^H, ^19^F and ^13^C NMR spectra**

**
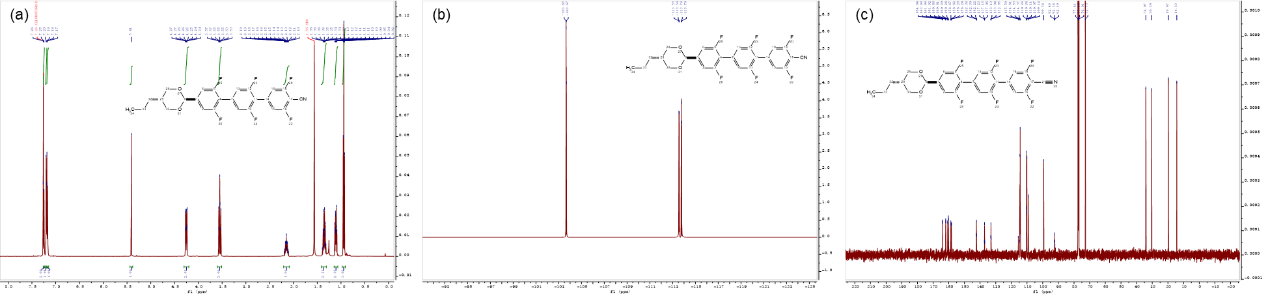
**

**Scheme S4**. (a) ^1^H, (b) ^19^F and (c) ^13^C NMR spectra of SCUT1a trans.

**SCUT1a** trans: ^1^H NMR (500 MHz, Chloroform-*d*) δ 7.25 (s, 2H), 7.20 (d, *J* = 5.9 Hz, 2H), 7.18 (d, *J* = 6.3 Hz, 2H), 5.41 (s, 1H), 4.30 – 4.21 (m, 2H), 3.59 – 3.51 (m, 2H), 2.21 – 2.09 (m, 1H), 1.38 – 1.32 (m, 2H), 1.14 – 1.08 (m, 2H), 0.94 (t, *J* = 7.3 Hz, 3H). ^19^F NMR (471 MHz, Chloroform-*d*) δ -103.66 (d, *J* = 9.3 Hz), -113.57 (d, *J* = 10.8 Hz), -113.77 (d, *J* = 11.0 Hz). ^13^C NMR (126 MHz, Chloroform-*d*) δ 164.02 (d, *J* = 5.4 Hz), 161.94 (d, *J* = 4.8 Hz), 160.63 (d, *J* = 6.6 Hz), 160.27 (d, *J* = 6.9 Hz), 158.64 (d, *J* = 6.4 Hz), 158.27 (d, *J* = 6.9 Hz), 142.30 (t, *J* = 9.7 Hz), 137.15 (t, *J* = 10.3 Hz), 133.04 (t, *J* = 11.2 Hz), 115.59, 114.81, 114.53, 114.37, 110.38 – 109.97 (m), 109.14, 99.34, 92.49, 72.73, 34.07, 30.39, 19.67, 14.33.

**
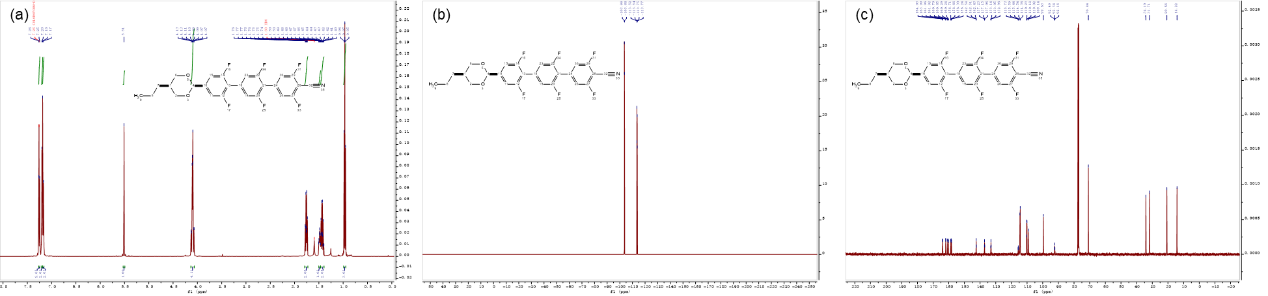
**

**Scheme S5**. (a) ^1^H, (b) ^19^F and (c) ^13^C NMR spectra of SCUT1a cis.

**SCUT1a** cis: ^1^H NMR (500 MHz, Chloroform-*d*) δ 7.25 (d, *J* = 8.9 Hz, 2H), 7.19 (d, *J* = 8.1 Hz, 2H), 7.17 (s, 2H), 5.51 (s, 1H), 4.13 – 4.06 (m, 4H), 1.79 – 1.73 (m, 2H), 1.50 – 1.46 (m, 1H), 1.46 – 1.39 (m, 2H), 0.97 (t, *J* = 7.3 Hz, 3H). ^19^F NMR (471 MHz, Chloroform-d) δ -103.67 (d, J = 8.7 Hz), -113.53 (d, J = 8.8 Hz), -113.76 (d, J = 9.4 Hz). ^13^C NMR (126 MHz, Chloroform-*d*) δ 164.01 (d, *J* = 4.9 Hz), 161.94 (d, *J* = 5.2 Hz), 160.68 (d, *J* = 6.6 Hz), 160.27 (d, *J* = 6.6 Hz), 158.68 (d, *J* = 6.6 Hz), 158.27 (d, *J* = 6.9 Hz), 142.51 (t, *J* = 9.6 Hz), 137.15 (t, *J* = 10.6 Hz), 133.05 (t, *J* = 11.1 Hz), 115.59 (t, *J* = 17.9 Hz), 114.45 (d, *J* = 22.4 Hz), 110.34 – 109.91 (m), 109.14, 99.63, 92.34 (t, *J* = 19.0 Hz), 70.84, 34.10, 31.71, 20.68, 14.22.


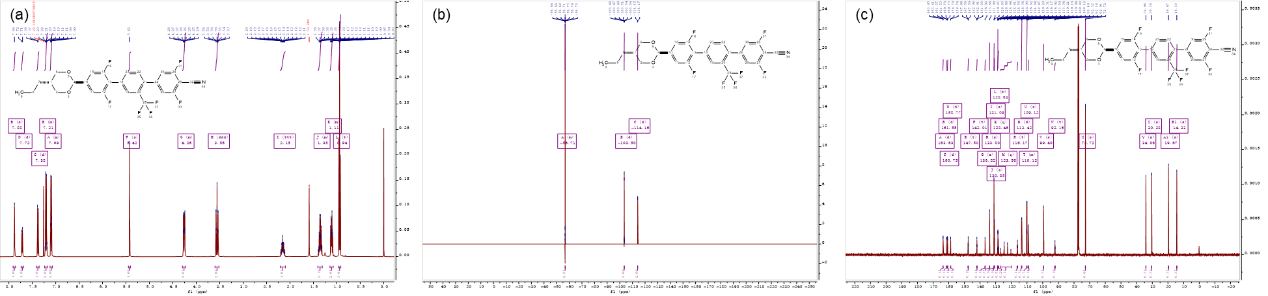


**Scheme S6**. (a) ^1^H, (b) ^19^F and (c) ^13^C NMR spectra of SCUT1b.

**SCUT1b**: ^1^H NMR (500 MHz, Chloroform-*d*) δ 7.88 (s, 1H), 7.72 (d, *J* = 7.9 Hz, 1H), 7.38 (d, *J* = 8.0 Hz, 1H), 7.24 – 7.18 (m, 2H), 7.12 – 7.07 (m, 2H), 5.42 (s, 1H), 4.30 – 4.23 (m, 2H), 3.56 (ddd, *J* = 11.6, 10.3, 1.6 Hz, 2H), 2.15 (ttt, *J* = 11.5, 7.0, 4.7 Hz, 1H), 1.41 – 1.31 (m, 2H), 1.15 – 1.08 (m, 2H), 0.94 (t, *J* = 7.3 Hz, 3H). ^19^F NMR (471 MHz, Chloroform-*d*) δ -56.65 – -56.78 (m), -103.50 (d, *J* = 8.4 Hz), -114.16 (d, *J* = 8.1 Hz). ^13^C NMR (126 MHz, Chloroform-*d*) δ 163.63 (d, *J* = 4.9 Hz), 161.55 (d, *J* = 5.1 Hz), 160.75 (d, *J* = 6.8 Hz), 158.77 (d, *J* = 6.8 Hz), 147.58 (t, *J* = 10.1 Hz), 142.01 (t, *J* = 9.6 Hz), 136.82, 133.88, 131.09, 130.85, 128.68 – 128.60 (m), 128.46 (q, *J* = 30.7 Hz), 123.56 (q, *J* = 274.0 Hz), 116.17 (t, *J* = 18.2 Hz), 113.42 (d, *J* = 20.4 Hz), 110.29 – 109.90 (m), 109.12, 99.40, 92.16 (t, *J* = 19.0 Hz), 72.72, 34.06, 30.38, 19.67, 14.33.


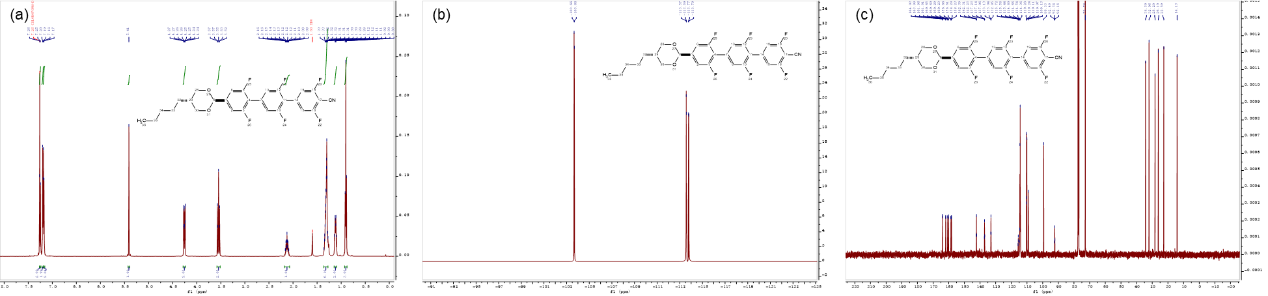


**Scheme S7**. (a) ^1^H, (b) ^19^F and (c) ^13^C NMR spectra of SCUT1c trans.

**SCUT1c** trans: ^1^H NMR (500 MHz, Chloroform-*d*) δ 7.25 (d, *J* = 8.3 Hz, 2H), 7.19 (d, *J* = 5.4 Hz, 2H), 7.18 (d, *J* = 5.7 Hz, 2H), 5.41 (s, 1H), 4.28 – 4.23 (m, 2H), 3.58 – 3.51 (m, 2H), 2.13 (tddd, *J* = 11.4, 9.2, 7.0, 4.6 Hz, 1H), 1.36 – 1.26 (m, 6H), 1.12 (dt, *J* = 8.8, 6.7 Hz, 2H), 0.93 – 0.88 (m, 3H). ^19^F NMR (471 MHz, Chloroform-*d*) δ -103.67 (d, *J* = 11.0 Hz), -113.58 (d, *J* = 8.9 Hz), -113.78 (d, *J* = 11.0 Hz). ^13^C NMR (126 MHz, Chloroform-*d*) δ 164.01 (d, *J* = 5.3 Hz), 161.93 (d, *J* = 5.2 Hz), 160.63 (d, *J* = 6.5 Hz), 160.26 (d, *J* = 6.9 Hz), 158.64 (d, *J* = 6.6 Hz), 158.26 (d, *J* = 7.0 Hz), 142.31 (t, *J* = 9.6 Hz), 137.14 (t, *J* = 10.5 Hz), 133.04 (t, *J* = 11.2 Hz), 115.58 (t, *J* = 17.7 Hz), 114.73 (d, *J* = 17.5 Hz), 114.54, 114.36, 110.38 – 109.97 (m), 109.13, 99.33, 92.34 (t, *J* = 19.2 Hz), 72.76, 34.30, 32.06, 28.20, 26.10, 22.59, 14.13.


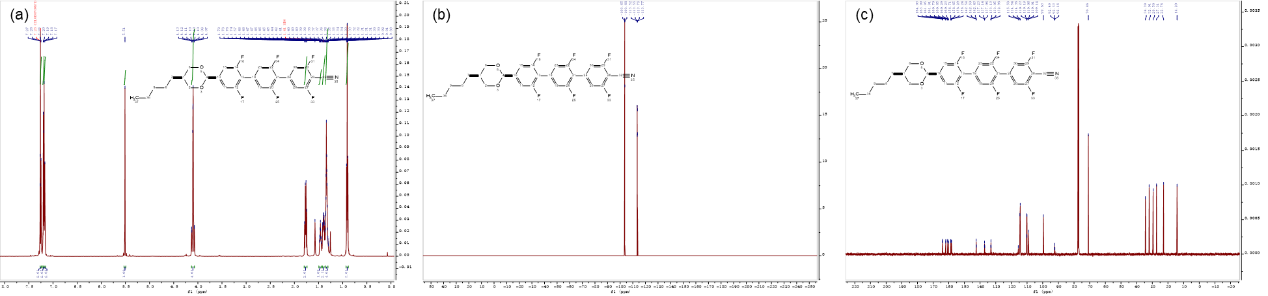


**Scheme S8**. (a) ^1^H, (b) ^19^F and (c) ^13^C NMR spectra of SCUT1c cis.

**SCUT1c** cis: ^1^H NMR (500 MHz, Chloroform-*d*) δ 7.26 (d, *J* = 8.5 Hz, 2H), 7.19 (d, *J* = 6.8 Hz, 2H), 7.18 (d, *J* = 7.4 Hz, 2H), 5.51 (s, 1H), 4.13 – 4.07 (m, 4H), 1.77 (q, *J* = 7.4 Hz, 2H), 1.46 (tt, *J* = 7.3, 2.1 Hz, 1H), 1.39 (ddd, *J* = 15.1, 7.4, 2.2 Hz, 2H), 1.33 (dtd, *J* = 8.7, 6.0, 3.8 Hz, 4H), 0.91 (t, *J* = 6.9 Hz, 3H). ^19^F NMR (471 MHz, Chloroform-*d*) δ -103.67 (d, *J* = 9.5 Hz), -113.52, -113.76 (d, *J* = 10.6 Hz). ^13^C NMR (126 MHz, Chloroform-*d*) δ 164.01 (d, *J* = 4.9 Hz), 161.93 (d, *J* = 5.2 Hz), 160.67 (d, *J* = 6.6 Hz), 160.26 (d, *J* = 6.9 Hz), 158.68 (d, *J* = 6.6 Hz), 158.26 (d, *J* = 6.8 Hz), 142.50 (t, *J* = 9.7 Hz), 137.14 (t, *J* = 10.5 Hz), 133.05 (t, *J* = 11.2 Hz), 114.45 (d, *J* = 22.4 Hz), 110.32 – 109.93 (m), 109.14, 99.63, 92.33 (t, *J* = 19.2 Hz), 70.86, 34.39, 32.01, 29.50, 27.31, 22.78, 14.20.


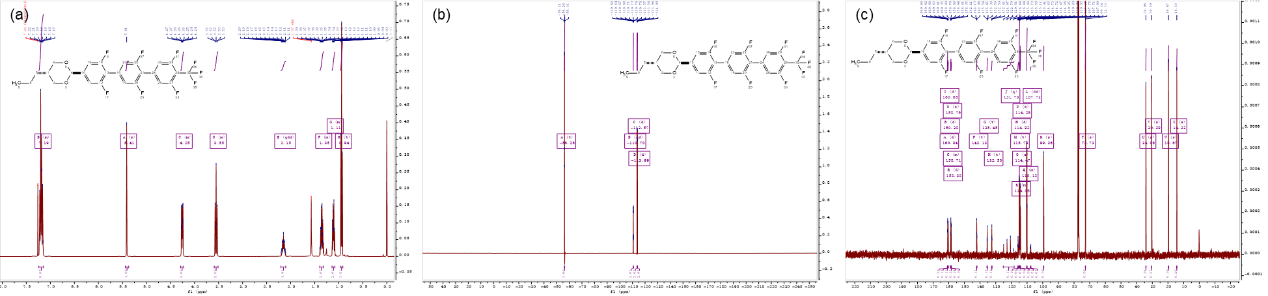


**Scheme S9**. (a) ^1^H, (b) ^19^F and (c) ^13^C NMR spectra of SCUT2a trans.

**SCUT2a** trans: ^1^H NMR (500 MHz, Chloroform-*d*) δ 7.24 – 7.14 (m, 6H), 5.41 (s, 1H), 4.29 – 4.22 (m, 2H), 3.59 – 3.51 (m, 2H), 2.15 (qdd, *J* = 11.4, 7.0, 4.7 Hz, 1H), 1.40 – 1.31 (m, 2H), 1.14 – 1.08 (m, 2H), 0.94 (t, *J* = 7.3 Hz, 3H). ^19^F NMR (471 MHz, Chloroform-*d*) δ -56.26 (t, *J* = 22.2 Hz), -110.70 (qd, *J* = 22.5, 21.7, 11.3 Hz), -113.57 (d, *J* = 8.5 Hz), -113.99 (d, *J* = 8.6 Hz). ^13^C NMR (126 MHz, Chloroform-*d*) δ 160.84 (d, *J* = 4.7 Hz), 160.65 (d, *J* = 6.6 Hz), 160.38 (d, *J* = 7.1 Hz), 158.76, 158.90 – 158.54 (m), 158.38 (d, *J* = 7.2 Hz), 142.13 (t, *J* = 9.7 Hz), 135.45 (t, *J* = 11.2 Hz), 132.50 (t, *J* = 11.0 Hz), 121.70 (q, *J* = 273.8 Hz), 115.73 (t, *J* = 18.0 Hz), 114.92 (d, *J* = 22.8 Hz), 115.14 – 114.70 (m), 114.47, 114.26, 110.35 – 109.93 (m), 107.73 (dd, *J* = 19.5, 14.5 Hz), 99.36, 72.73, 34.06, 30.39, 19.67, 14.33.


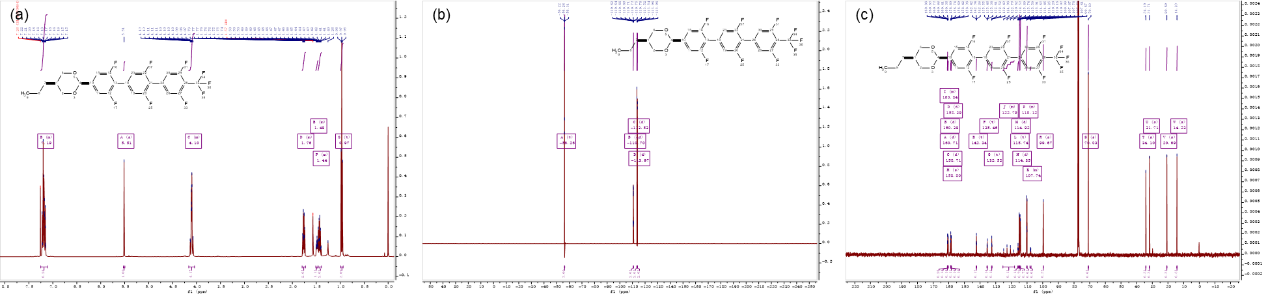


**Scheme S10**. (a) ^1^H, (b) ^19^F and (c) ^13^C NMR spectra of SCUT2a cis.

**SCUT2a** cis: ^1^H NMR (500 MHz, Chloroform-*d*) δ 7.25 – 7.12 (m, 6H), 5.51 (s, 1H), 4.16 – 4.04 (m, 4H), 1.80 – 1.73 (m, 2H), 1.51 – 1.46 (m, 1H), 1.46 – 1.39 (m, 2H), 0.97 (t, *J* = 7.3 Hz, 3H). ^19^F NMR (471 MHz, Chloroform-*d*) δ -56.26 (t, *J* = 21.7 Hz), -110.70 (qd, *J* = 21.9, 10.9 Hz), -113.53 (d, *J* = 8.6 Hz), -113.97 (d, *J* = 8.9 Hz). ^13^C NMR (126 MHz, Chloroform-*d*) δ 160.92 – 160.77 (m), 160.71 (d, *J* = 6.5 Hz), 160.39 (d, *J* = 7.1 Hz), 158.80, 158.71 (d, *J* = 6.7 Hz), 158.39 (d, *J* = 7.0 Hz), 142.34 (t, *J* = 9.6 Hz), 135.46 (t, *J* = 11.4 Hz), 132.52 (t, *J* = 11.2 Hz), 125.39 – 117.97 (m), 115.74 (t, *J* = 18.4 Hz), 114.92 (d, *J* = 23.1 Hz), 114.35 (d, *J* = 25.9 Hz), 110.30 – 109.89 (m), 108.16 – 107.41 (m), 99.67, 70.83, 34.10, 31.71, 20.69, 14.23.


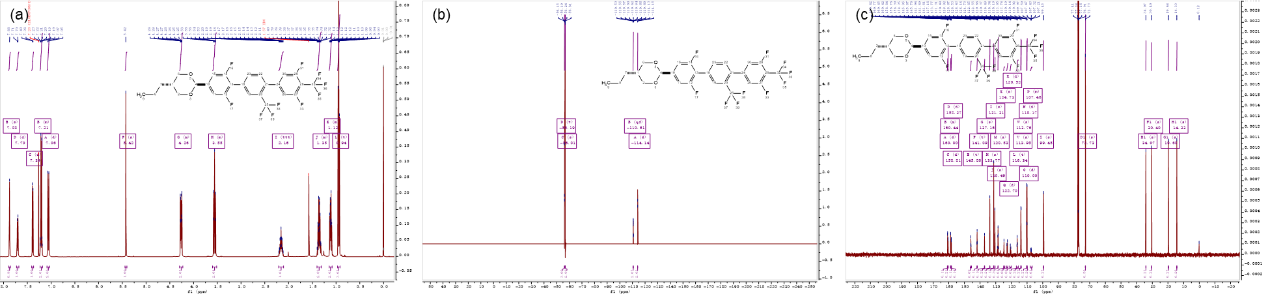


**Scheme S11**. (a) ^1^H, (b) ^19^F and (c) ^13^C NMR spectra of SCUT2b trans.

**SCUT2b** trans: ^1^H NMR (500 MHz, Chloroform-*d*) δ 7.88 (s, 1H), 7.70 (d, *J* = 7.9 Hz, 1H), 7.39 (d, *J* = 8.0 Hz, 1H), 7.23 – 7.18 (m, 2H), 7.06 (d, *J* = 10.0 Hz, 2H), 5.42 (s, 1H), 4.30 – 4.23 (m, 2H), 3.60 – 3.51 (m, 2H), 2.16 (ttt, *J* = 11.5, 6.9, 4.6 Hz, 1H), 1.40 – 1.31 (m, 2H), 1.15 – 1.08 (m, 2H), 0.94 (t, *J* = 7.3 Hz, 3H). ^19^F NMR (471 MHz, Chloroform-*d*) δ -56.19 (t, *J* = 22.1 Hz), -56.81, -110.61 (qd, *J* = 22.3, 10.5 Hz), -114.14 (d, *J* = 9.5 Hz). ^13^C NMR (126 MHz, Chloroform-*d*) δ 160.80 (d, *J* = 6.8 Hz), 160.54 – 160.33 (m), 158.81 (d, *J* = 7.0 Hz), 158.37 (d, *J* = 4.1 Hz), 145.86 (t, *J* = 10.5 Hz), 141.89 (t, *J* = 9.7 Hz), 137.16, 133.77, 131.31, 130.49, 128.53, 124.73, 122.70 (d, *J* = 38.1 Hz), 120.52 (d, *J* = 38.5 Hz), 116.34 (t, *J* = 18.4 Hz), 113.95, 113.76, 110.17 (d, *J* = 6.1 Hz), 110.00 (d, *J* = 6.0 Hz), 107.99 – 106.70 (m), 99.45, 72.73, 34.07, 30.40, 19.68, 14.33.


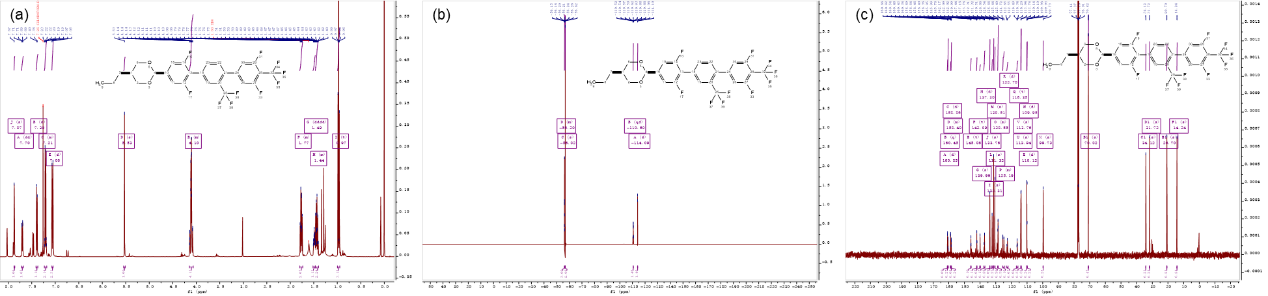


**Scheme S12**. (a) ^1^H, (b) ^19^F and (c) ^13^C NMR spectra of SCUT2b cis.

**SCUT2b** cis: ^1^H NMR (500 MHz, Chloroform-*d*) δ 7.87 (s, 1H), 7.70 (dd, *J* = 8.1, 1.5 Hz, 1H), 7.39 (d, *J* = 8.0 Hz, 1H), 7.23 – 7.18 (m, 2H), 7.06 (d, *J* = 10.1 Hz, 2H), 5.53 (s, 1H), 4.15 – 4.06 (m, 4H), 1.80 – 1.74 (m, 2H), 1.49 (dddd, *J* = 9.1, 6.4, 2.8, 1.4 Hz, 1H), 1.47 – 1.40 (m, 2H), 0.97 (t, *J* = 7.3 Hz, 3H). ^19^F NMR (471 MHz, Chloroform-*d*) δ -56.12 – -56.30 (m), -56.82, -110.60 (qd, *J* = 22.5, 11.0 Hz), -114.09 (d, *J* = 8.2 Hz). ^13^C NMR (126 MHz, Chloroform-*d*) δ 160.85 (d, *J* = 6.9 Hz), 160.45 (q, *J* = 8.8, 8.0 Hz), 158.86 (d, *J* = 6.7 Hz), 158.55 – 158.27 (m), 145.86 (t, *J* = 10.7 Hz), 142.09 (t, *J* = 9.5 Hz), 139.99, 137.30 (d, *J* = 34.5 Hz), 133.76, 132.31, 131.32, 130.51, 128.77 – 128.35 (m), 125.53 – 124.49 (m), 122.70 (d, *J* = 38.1 Hz), 116.35 (t, *J* = 18.3 Hz), 113.94, 113.76, 110.12 (d, *J* = 6.2 Hz), 109.95 (d, *J* = 6.1 Hz), 99.73, 70.82, 34.12, 31.72, 20.70, 14.24.


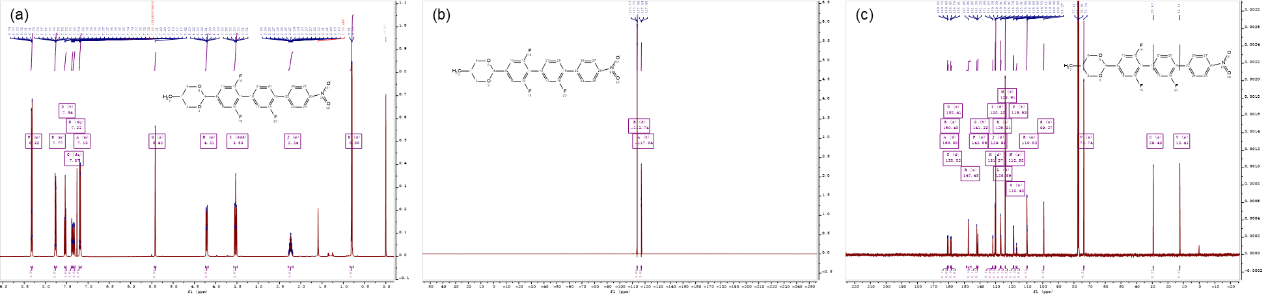


**Scheme S13**. (a) ^1^H, (b) ^19^F and (c) ^13^C NMR spectra of SCUT3a.

**SCUT3a**: ^1^H NMR (500 MHz, Chloroform-*d*) δ 8.34 – 8.30 (m, 2H), 7.79 – 7.74 (m, 2H), 7.54 (t, *J* = 8.0 Hz, 1H), 7.37 (dq, *J* = 8.0, 1.5 Hz, 1H), 7.33 (dq, *J* = 11.5, 1.4 Hz, 1H), 7.21 – 7.16 (m, 2H), 5.42 (s, 1H), 4.24 – 4.17 (m, 2H), 3.53 (ddd, *J* = 11.6, 10.3, 1.6 Hz, 2H), 2.30 – 2.18 (m, 1H), 0.80 (d, *J* = 6.8 Hz, 3H). ^19^F NMR (471 MHz, Chloroform-*d*) δ -113.74 (d, *J* = 8.5 Hz), -117.04 (t, *J* = 10.7 Hz). ^13^C NMR (126 MHz, Chloroform-*d*) δ 160.80 (d, *J* = 7.0 Hz), 160.40, 158.82 (d, *J* = 6.9 Hz), 158.41, 147.45, 142.06, 141.39 (t, *J* = 9.9 Hz), 131.87 (d, *J* = 8.8 Hz), 130.38 (d, *J* = 3.3 Hz), 129.99 (d, *J* = 3.4 Hz), 126.91, 126.69 (d, *J* = 13.1 Hz), 123.91, 118.68, 118.48, 116.69 (t, *J* = 17.8 Hz), 110.18 – 109.82 (m), 99.27, 73.74, 29.43, 12.41.


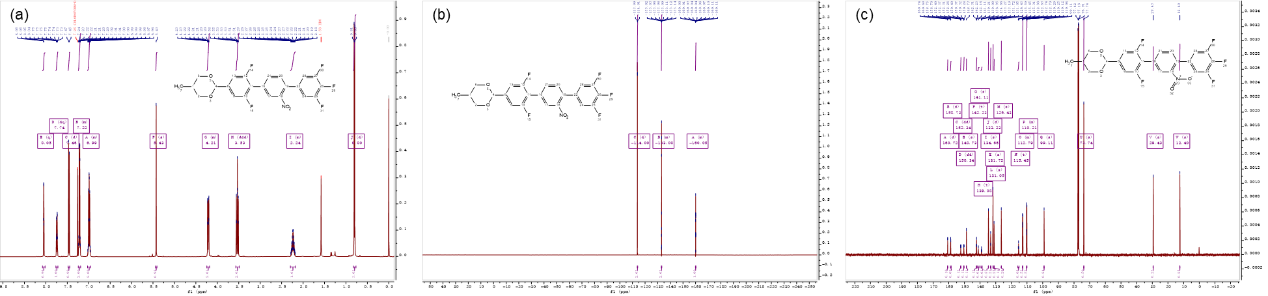


**Scheme S14**. (a) ^1^H, (b) ^19^F and (c) ^13^C NMR spectra of SCUT3b.

**SCUT3b**: ^1^H NMR (500 MHz, Chloroform-*d*) δ 8.05 (q, *J* = 1.3 Hz, 1H), 7.74 (dq, *J* = 8.0, 1.5 Hz, 1H), 7.46 (d, *J* = 8.0 Hz, 1H), 7.24 – 7.19 (m, 2H), 7.03 – 6.95 (m, 2H), 5.43 (s, 1H), 4.24 – 4.18 (m, 2H), 3.53 (ddd, *J* = 11.6, 10.3, 1.6 Hz, 2H), 2.30 – 2.18 (m, 1H), 0.80 (d, *J* = 6.8 Hz, 3H). ^19^F NMR (471 MHz, Chloroform-*d*) δ -114.00 (d, *J* = 9.0 Hz), -132.84 – -133.28 (m), -159.89 – -160.27 (m). ^13^C NMR (126 MHz, Chloroform-*d*) δ 160.72 (d, *J* = 6.7 Hz), 158.73 (d, *J* = 6.5 Hz), 152.34 (dd, *J* = 10.5, 3.5 Hz), 150.34 (dd, *J* = 10.2, 3.9 Hz), 148.73, 142.23 (t, *J* = 9.6 Hz), 141.11, 139.08 (t, *J* = 16.1 Hz), 134.66, 133.23 (d, *J* = 9.8 Hz), 131.72, 131.05, 126.43, 115.45 (t, *J* = 17.8 Hz), 112.95 – 112.62 (m), 110.37 – 110.00 (m), 99.11, 73.74, 29.43, 12.40.


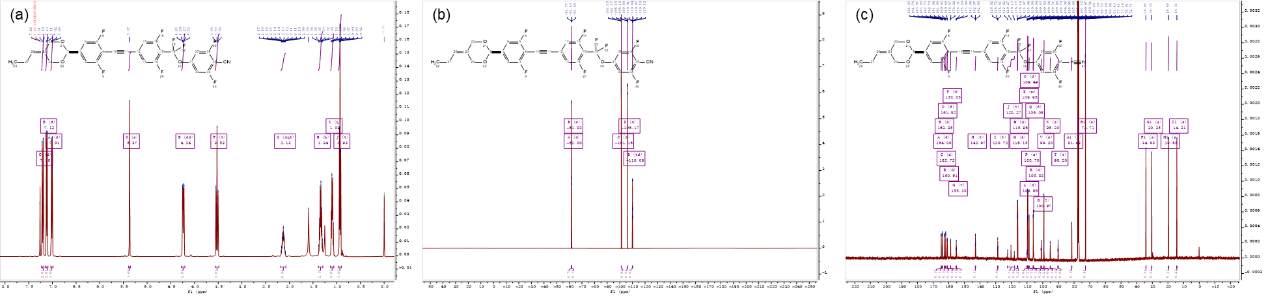


**Scheme S15**. (a) ^1^H, (b) ^19^F and (c) ^13^C NMR spectra of SCUT4a.

**SCUT4a**: ^1^H NMR (500 MHz, Chloroform-d) δ 7.20 (d, J = 10.1 Hz, 2H), 7.12 (d, J = 8.0 Hz, 2H), 7.01 (d, J = 8.6 Hz, 2H), 5.37 (s, 1H), 4.24 (dd, J = 11.3, 4.5 Hz, 2H), 3.53 (t, J = 11.2 Hz, 2H), 2.13 (dqt, J = 11.1, 6.9, 3.4 Hz, 1H), 1.34 (h, J = 7.4 Hz, 2H), 1.09 (q, J = 7.4 Hz, 2H), 0.93 (t, J = 7.3 Hz, 3H). ^19^F NMR (471 MHz, Chloroform-d) δ -61.80 (d, J = 25.6 Hz), -61.88, -101.16 (d, J = 8.7 Hz), -106.17 (d, J = 8.3 Hz), -110.06 (td, J = 25.6, 9.6 Hz). ^13^C NMR (126 MHz, Chloroform-d) δ 164.80 (d, J = 6.5 Hz), 163.95 (d, J = 5.3 Hz), 162.72 (d, J = 6.5 Hz), 161.92 (d, J = 5.2 Hz), 160.91 (d, J = 5.0 Hz), 158.85 (d, J = 5.6 Hz), 155.20 (t, J = 13.6 Hz), 142.97 (t, J = 9.2 Hz), 128.72 (t, J = 12.9 Hz), 120.27 (t, J = 269.8 Hz), 116.15 (d, J = 4.0 Hz), 115.96 (d, J = 3.7 Hz), 110.13 – 109.78 (m), 109.66 (d, J = 4.5 Hz), 109.49 (d, J = 4.6 Hz), 108.70, 106.06 (d, J = 3.9 Hz), 105.88 (d, J = 3.6 Hz), 100.97 (t, J = 19.6 Hz), 99.20, 95.20, 90.20 (t, J = 19.4 Hz), 81.43, 72.71, 34.03, 30.35, 19.65, 14.31.

_
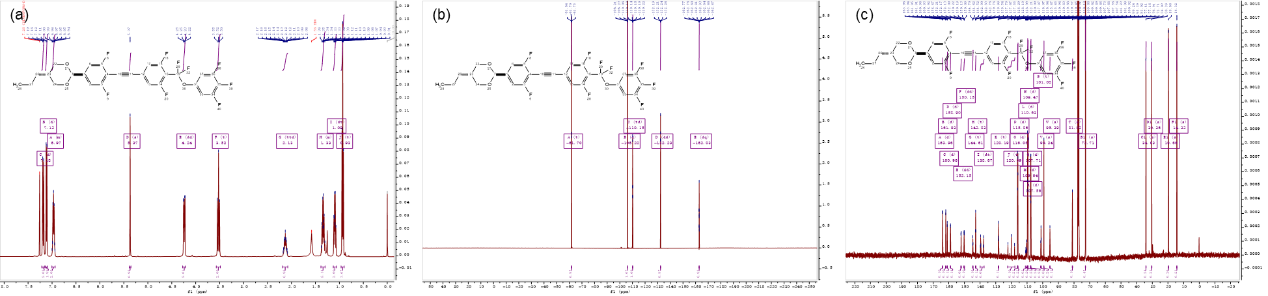
_

**Scheme S16**. (a) ^1^H, (b) ^19^F and (c) ^13^C NMR spectra of SCUT4b.

**SCUT4b**: ^1^H NMR (500 MHz, Chloroform-d) δ 7.18 (d, J = 9.5 Hz, 2H), 7.12 (d, J = 7.8 Hz, 2H), 7.00 – 6.93 (m, 2H), 5.37 (s, 1H), 4.24 (dd, J = 11.9, 4.6 Hz, 2H), 3.52 (t, J = 11.5 Hz, 2H), 2.13 (ttd, J = 11.2, 6.8, 3.6 Hz, 1H), 1.39 – 1.30 (m, 2H), 1.09 (dt, J = 10.3, 7.2 Hz, 2H), 0.93 (t, J = 7.3 Hz, 3H). ^19^F NMR (471 MHz, Chloroform-d) δ -61.70 (t, J = 26.8 Hz), -106.22 (d, J = 8.4 Hz), -110.15 (td, J = 27.9, 11.0 Hz), -132.23 (dd, J = 22.1, 8.1 Hz), -162.83 (dq, J = 20.1, 7.2, 5.2 Hz). ^13^C NMR (126 MHz, Chloroform-d) δ 163.96 (d, J = 5.0 Hz), 161.93 (d, J = 5.3 Hz), 160.96 (d, J = 5.6 Hz), 158.90 (d, J = 5.8 Hz), 152.15 (dd, J = 10.7, 5.2 Hz), 150.15 (dd, J = 10.8, 5.1 Hz), 144.61 (t, J = 10.8 Hz), 142.83 (t, J = 9.3 Hz), 138.67 (dt, J = 250.6, 15.3 Hz), 128.19 (t, J = 12.8 Hz), 120.09 (t, J = 266.4 Hz), 116.05 (d, J = 4.2 Hz), 115.86 (d, J = 3.2 Hz), 110.63 (d, J = 14.0 Hz), 109.64 (d, J = 4.7 Hz), 109.47 (d, J = 4.6 Hz), 107.71 (d, J = 6.0 Hz), 107.56 (d, J = 5.9 Hz), 101.08 (t, J = 19.6 Hz), 99.24, 95.39, 81.02, 72.71, 34.03, 30.36, 19.66, 14.32.


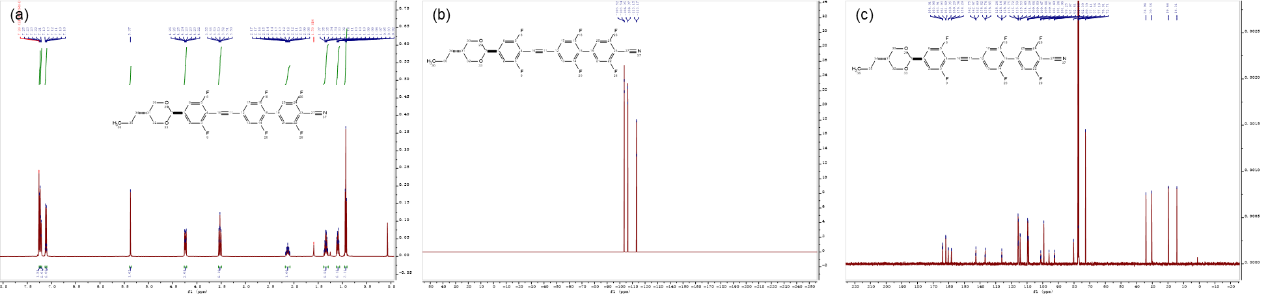


**Scheme S17**. (a) ^1^H, (b) ^19^F and (c) ^13^C NMR spectra of SCUT5a.

**SCUT5a**: ^1^H NMR (500 MHz, Chloroform-*d*) δ 7.24 (d, *J* = 8.3 Hz, 2H), 7.22 (d, *J* = 8.0 Hz, 2H), 7.13 – 7.10 (m, 2H), 5.37 (s, 1H), 4.26 – 4.21 (m, 2H), 3.56 – 3.50 (m, 2H), 2.18 – 2.08 (m, 1H), 1.39 – 1.29 (m, 2H), 1.13 – 1.06 (m, 2H), 0.93 (t, *J* = 7.3 Hz, 3H). ^19^F NMR (471 MHz, Chloroform-*d*) δ -103.53 (d, *J* = 9.8 Hz), -106.36 (d, *J* = 8.7 Hz), -113.16 (d, *J* = 9.4 Hz). ^13^C NMR (126 MHz, Chloroform-*d*) δ 164.09 – 163.83 (m), 161.91 (d, *J* = 5.2 Hz), 160.32 (d, *J* = 7.3 Hz), 142.67 (t, *J* = 9.3 Hz), 126.14 (t, *J* = 12.6 Hz), 115.83 – 115.46 (m), 114.46, 114.29, 109.72 – 109.36 (m), 99.25, 95.97, 77.41, 77.16, 76.91, 72.71, 34.04, 30.36, 19.66, 14.31.


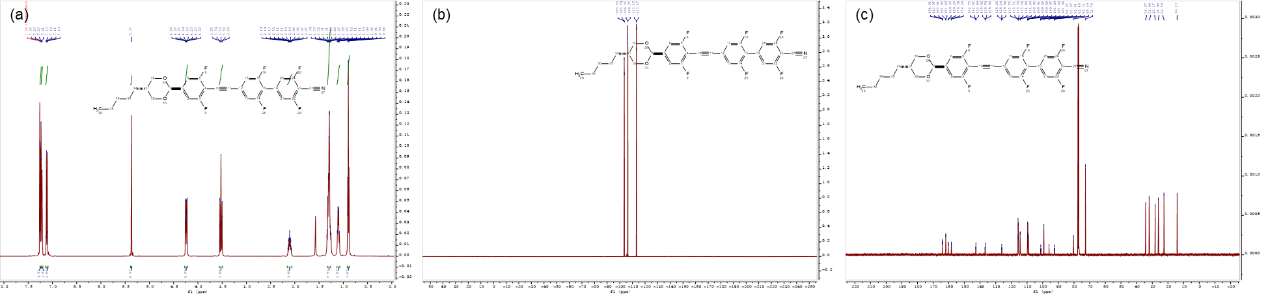


**Scheme S18**. (a) ^1^H, (b) ^19^F and (c) ^13^C NMR spectra of SCUT5b.

**SCUT5b**: ^1^H NMR (500 MHz, Chloroform-*d*) δ 7.24 (d, *J* = 8.3 Hz, 2H), 7.22 (d, *J* = 8.3 Hz, 2H), 7.11 (d, *J* = 7.8 Hz, 2H), 5.37 (s, 1H), 4.27 – 4.21 (m, 2H), 3.52 (t, *J* = 11.5 Hz, 2H), 2.11 (ddt, *J* = 13.5, 11.2, 6.8 Hz, 1H), 1.33 – 1.26 (m, 6H), 1.10 (dt, *J* = 8.8, 6.7 Hz, 2H), 0.90 (t, *J* = 6.8 Hz, 3H). ^19^F NMR (471 MHz, Chloroform-*d*) δ -103.53 (d, *J* = 10.5 Hz), -106.36 (d, *J* = 9.4 Hz), -113.16 (d, *J* = 8.6 Hz). ^13^C NMR (126 MHz, Chloroform-*d*) δ 163.96 (t, *J* = 6.3 Hz), 161.91 (d, *J* = 5.1 Hz), 160.32 (d, *J* = 7.2 Hz), 158.32 (d, *J* = 7.0 Hz), 142.68 (t, *J* = 9.3 Hz), 136.74 (t, *J* = 10.6 Hz), 126.14 (t, *J* = 12.5 Hz), 115.83 – 115.46 (m), 114.46, 114.29, 109.72 – 109.37 (m), 99.25, 95.97, 92.46 (t, *J* = 19.3 Hz), 80.30, 72.74, 34.27, 32.04, 28.17, 26.09, 22.59, 14.13.


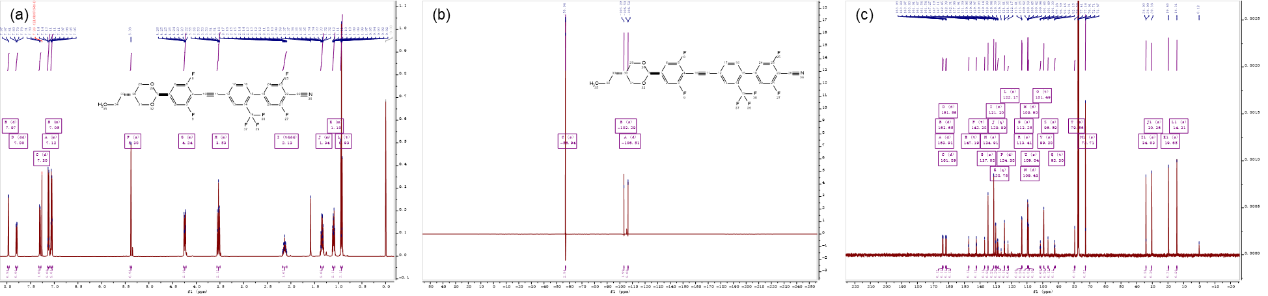


**Scheme S19**. (a) ^1^H, (b) ^19^F and (c) ^13^C NMR spectra of SCUT5c.

**SCUT5c**: ^1^H NMR (500 MHz, Chloroform-*d*) δ 7.97 (d, *J* = 1.6 Hz, 1H), 7.80 (dd, *J* = 8.0, 1.6 Hz, 1H), 7.30 (d, *J* = 7.9 Hz, 1H), 7.15 – 7.10 (m, 2H), 7.07 – 7.04 (m, 2H), 5.38 (s, 1H), 4.27 – 4.21 (m, 2H), 3.57 – 3.50 (m, 2H), 2.13 (tddd, *J* = 11.5, 9.1, 7.0, 4.7 Hz, 1H), 1.37 – 1.31 (m, 2H), 1.12 – 1.07 (m, 2H), 0.93 (t, *J* = 7.3 Hz, 3H). ^19^F NMR (471 MHz, Chloroform-*d*) δ -56.94, -103.29, -106.51 (d, *J* = 8.3 Hz). ^13^C NMR (126 MHz, Chloroform-*d*) δ 163.91 (d, *J* = 5.4 Hz), 163.65 (d, *J* = 4.9 Hz), 161.89 (d, *J* = 5.4 Hz), 161.56 (d, *J* = 5.0 Hz), 147.19 (t, *J* = 10.1 Hz), 142.38 (t, *J* = 9.2 Hz), 137.08, 134.91, 131.30, 129.99 (q, *J* = 5.3 Hz), 128.76 (q, *J* = 31.1 Hz), 124.38 (d, *J* = 7.2 Hz), 122.17, 113.41, 113.25, 109.60 (d, *J* = 4.9 Hz), 109.43 (d, *J* = 4.7 Hz), 109.04, 101.49 (t, *J* = 19.9 Hz), 99.28, 96.59, 92.30 (t, *J* = 19.3 Hz), 79.56, 72.71, 34.03, 30.36, 19.65, 14.31.


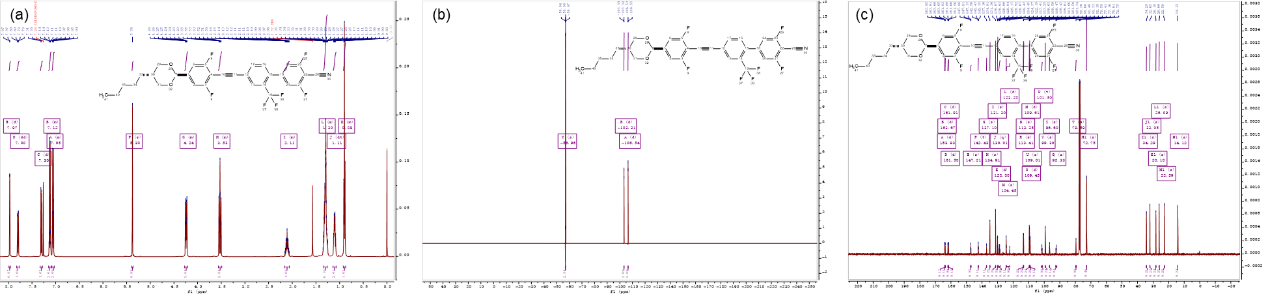


**Scheme S20**. (a) ^1^H, (b) ^19^F and (c) ^13^C NMR spectra of SCUT5d trans.

**SCUT5d** trans: ^1^H NMR (500 MHz, Chloroform-*d*) δ 7.97 (d, *J* = 1.6 Hz, 1H), 7.80 (dd, *J* = 7.9, 1.7 Hz, 1H), 7.30 (d, *J* = 8.0 Hz, 1H), 7.15 – 7.09 (m, 2H), 7.08 – 7.03 (m, 2H), 5.38 (s, 1H), 4.27 – 4.21 (m, 2H), 3.56 – 3.49 (m, 2H), 2.16 – 2.06 (m, 1H), 1.34 – 1.26 (m, 6H), 1.11 (dt, *J* = 8.5, 6.5 Hz, 2H), 0.92 – 0.87 (m, 3H). ^19^F NMR (471 MHz, Chloroform-*d*) δ -56.96, -103.31 (d, *J* = 9.0 Hz), -106.54 (d, *J* = 8.4 Hz). ^13^C NMR (126 MHz, Chloroform-*d*) δ 163.93 (d, *J* = 5.4 Hz), 163.67 (d, *J* = 4.8 Hz), 161.91 (d, *J* = 5.2 Hz), 161.58 (d, *J* = 4.8 Hz), 147.21, 142.43 (t, *J* = 9.4 Hz), 137.10, 134.91, 131.30, 130.01 (q, *J* = 5.0 Hz), 128.80 (d, *J* = 31.4 Hz), 124.45, 123.28 (d, *J* = 274.2 Hz), 113.41, 113.25, 109.61 (d, *J* = 4.7 Hz), 109.45 (d, *J* = 4.7 Hz), 109.01, 101.50 (t, *J* = 19.9 Hz), 99.30, 96.60, 92.33, 79.59, 72.75, 34.29, 32.05, 28.18, 26.09, 22.59, 14.12.


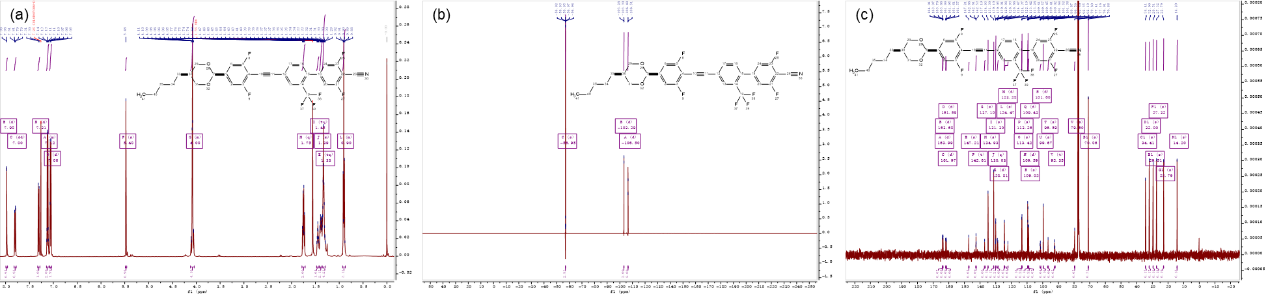


**Scheme S21**. (a) ^1^H, (b) ^19^F and (c) ^13^C NMR spectra of SCUT5d cis.

**SCUT5d** trans: ^1^H NMR (500 MHz, Chloroform-*d*) δ 7.98 (d, *J* = 1.6 Hz, 1H), 7.80 (dd, *J* = 8.1, 1.6 Hz, 1H), 7.31 (d, *J* = 8.0 Hz, 1H), 7.14 – 7.09 (m, 2H), 7.06 (d, *J* = 7.9 Hz, 2H), 5.48 (s, 1H), 4.12 – 4.04 (m, 4H), 1.75 (q, *J* = 7.5 Hz, 2H), 1.45 (tq, *J* = 7.3, 2.1 Hz, 1H), 1.42 – 1.36 (m, 2H), 1.33 (tq, *J* = 6.3, 3.6 Hz, 4H), 0.93 – 0.87 (m, 3H). ^19^F NMR (471 MHz, Chloroform-*d*) δ -56.95, -103.29 (d, *J* = 8.5 Hz), -106.50 (d, *J* = 10.2 Hz). ^13^C NMR (126 MHz, Chloroform-*d*) δ 163.99 (d, *J* = 5.4 Hz), 163.68 (d, *J* = 4.9 Hz), 161.97 (d, *J* = 5.0 Hz), 161.59 (d, *J* = 4.8 Hz), 147.21, 142.61 (t, *J* = 9.4 Hz), 137.10, 134.93, 131.30, 130.03 (q, *J* = 4.6, 3.8 Hz), 128.81 (d, *J* = 31.1 Hz), 124.47, 123.28 (d, *J* = 274.1 Hz), 113.42, 113.26, 109.59 (d, *J* = 4.5 Hz), 109.43 (d, *J* = 4.4 Hz), 109.02, 101.60 (d, *J* = 19.9 Hz), 99.67, 96.59, 92.35 (t, *J* = 19.4 Hz), 79.60, 70.86, 34.41, 32.00, 29.51, 27.32, 22.79, 14.20.


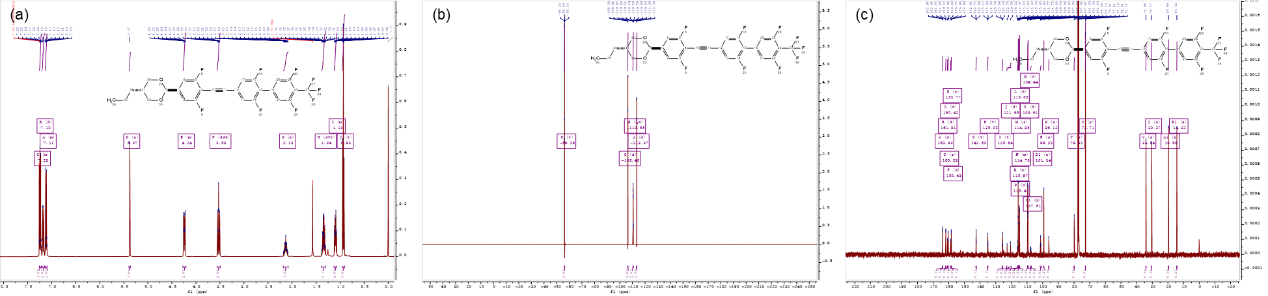


**Scheme S22**. (a) ^1^H, (b) ^19^F and (c) ^13^C NMR spectra of SCUT6a.

**SCUT6a**: ^1^H NMR (500 MHz, Chloroform-*d*) δ 7.26 – 7.21 (m, 2H), 7.18 (d, *J* = 10.5 Hz, 2H), 7.14 – 7.10 (m, 2H), 5.37 (s, 1H), 4.27 – 4.21 (m, 2H), 3.53 (ddd, *J* = 11.6, 10.3, 1.5 Hz, 2H), 2.18 – 2.08 (m, 1H), 1.34 (dtt, *J* = 10.2, 7.9, 4.3 Hz, 2H), 1.13 – 1.07 (m, 2H), 0.93 (t, *J* = 7.3 Hz, 3H). ^19^F NMR (471 MHz, Chloroform-*d*) δ -56.29 (t, *J* = 21.5 Hz), -106.40 (d, *J* = 7.8 Hz), -110.55 (qd, *J* = 22.3, 10.9 Hz), -113.37 (d, *J* = 8.5 Hz). ^13^C NMR (126 MHz, Chloroform-*d*) δ 163.93 (d, *J* = 5.3 Hz), 161.91 (d, *J* = 5.3 Hz), 160.83, 160.43 (d, *J* = 7.3 Hz), 158.77, 158.43 (d, *J* = 7.3 Hz), 142.53 (t, *J* = 9.3 Hz), 135.08 (t, *J* = 11.4 Hz), 125.64 (t, *J* = 12.5 Hz), 121.65 (d, *J* = 273.2 Hz), 115.97, 115.65 (d, *J* = 6.1 Hz), 115.48 (d, *J* = 6.0 Hz), 114.95, 114.85 – 114.67 (m), 109.61 (d, *J* = 4.7 Hz), 109.44 (d, *J* = 4.7 Hz), 108.06 – 107.29 (m), 101.34, 99.28, 96.12, 79.91, 72.71, 34.04, 30.37, 19.66, 14.32.


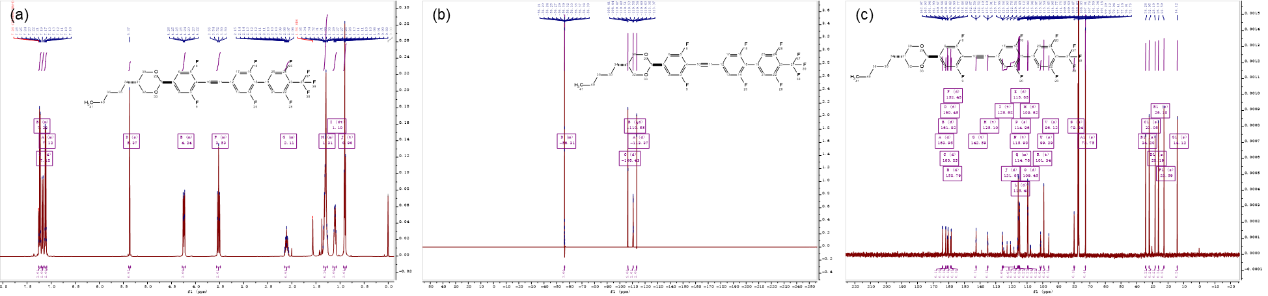


**Scheme S23**. (a) ^1^H, (b) ^19^F and (c) ^13^C NMR spectra of SCUT6b trans.

**SCUT6b** trans: ^1^H NMR (500 MHz, Chloroform-*d*) δ 7.26 – 7.21 (m, 2H), 7.18 (d, *J* = 10.4 Hz, 2H), 7.14 – 7.09 (m, 2H), 5.37 (s, 1H), 4.28 – 4.21 (m, 2H), 3.57 – 3.48 (m, 2H), 2.16 – 2.06 (m, 1H), 1.34 – 1.26 (m, 6H), 1.10 (dt, *J* = 8.8, 6.6 Hz, 2H), 0.90 (t, *J* = 6.9 Hz, 3H). ^19^F NMR (471 MHz, Chloroform-*d*) δ -56.18 – -56.42 (m), -106.43 (d, *J* = 11.1 Hz), -110.55 (qd, *J* = 21.8, 21.1, 10.9 Hz), -113.37 (d, *J* = 8.2 Hz). ^13^C NMR (126 MHz, Chloroform-*d*) δ 163.95 (d, *J* = 5.3 Hz), 161.92 (d, *J* = 5.4 Hz), 160.85 (d, *J* = 4.2 Hz), 160.45 (d, *J* = 7.3 Hz), 158.79 (d, *J* = 4.6 Hz), 158.45 (d, *J* = 7.3 Hz), 142.59 (t, *J* = 9.2 Hz), 135.10 (t, *J* = 11.3 Hz), 125.68 (t, *J* = 12.4 Hz), 121.67 (d, *J* = 273.8 Hz), 115.98 (t, *J* = 17.6 Hz), 115.65 (d, *J* = 6.3 Hz), 115.48 (d, *J* = 6.0 Hz), 114.96, 114.85 – 114.67 (m), 109.62 (d, *J* = 4.8 Hz), 109.45 (d, *J* = 4.5 Hz), 101.34 (t, *J* = 19.7 Hz), 99.29, 96.12, 79.94, 72.75, 34.28, 32.05, 28.19, 26.10, 22.59, 14.12.


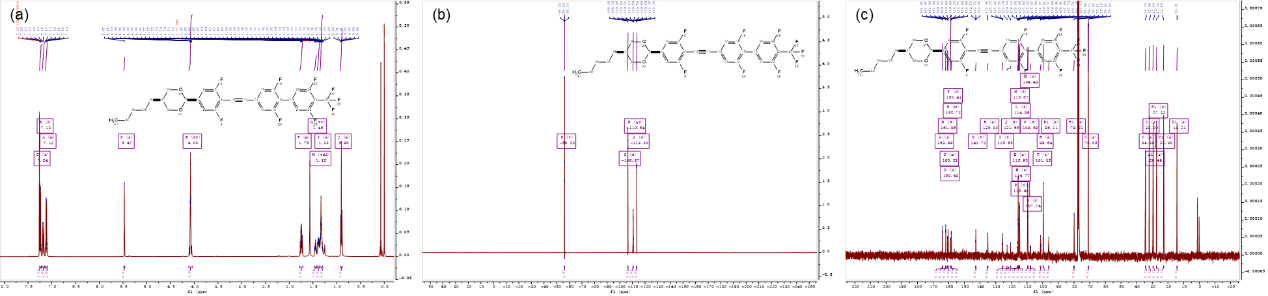


**Scheme S24**. (a) ^1^H, (b) ^19^F and (c) ^13^C NMR spectra of SCUT6b cis.

**SCUT6b** cis: ^1^H NMR (500 MHz, Chloroform-*d*) δ 7.24 (d, *J* = 8.1 Hz, 2H), 7.18 (d, *J* = 10.5 Hz, 2H), 7.14 – 7.09 (m, 2H), 5.47 (s, 1H), 4.08 (dt, *J* = 2.9, 1.4 Hz, 4H), 1.78 – 1.71 (m, 2H), 1.45 (tt, *J* = 7.3, 2.2 Hz, 1H), 1.38 (tdd, *J* = 6.9, 5.4, 2.6 Hz, 2H), 1.35 – 1.29 (m, 4H), 0.92 – 0.88 (m, 3H). ^19^F NMR (471 MHz, Chloroform-*d*) δ -56.28 (t, *J* = 21.9 Hz), -106.37 (d, *J* = 8.4 Hz), -110.54 (qd, *J* = 21.1, 19.9, 9.8 Hz), -113.38 (d, *J* = 8.5 Hz). ^13^C NMR (126 MHz, Chloroform-*d*) δ 163.99 (d, *J* = 5.1 Hz), 161.96 (d, *J* = 5.1 Hz), 160.96 – 160.71 (m), 160.43 (d, *J* = 7.5 Hz), 158.90 – 158.66 (m), 158.43 (d, *J* = 7.5 Hz), 142.72 (t, *J* = 9.3 Hz), 135.08, 125.65 (t, *J* = 12.5 Hz), 121.65 (d, *J* = 273.2 Hz), 115.98, 115.67 (d, *J* = 6.0 Hz), 115.49 (d, *J* = 5.9 Hz), 114.96, 114.77, 109.59 (d, *J* = 4.7 Hz), 109.43 (d, *J* = 4.6 Hz), 107.74, 101.35 (t, *J* = 19.8 Hz), 99.64, 96.11, 79.92, 70.85, 34.38, 32.00, 29.49, 27.32, 22.80, 14.21.


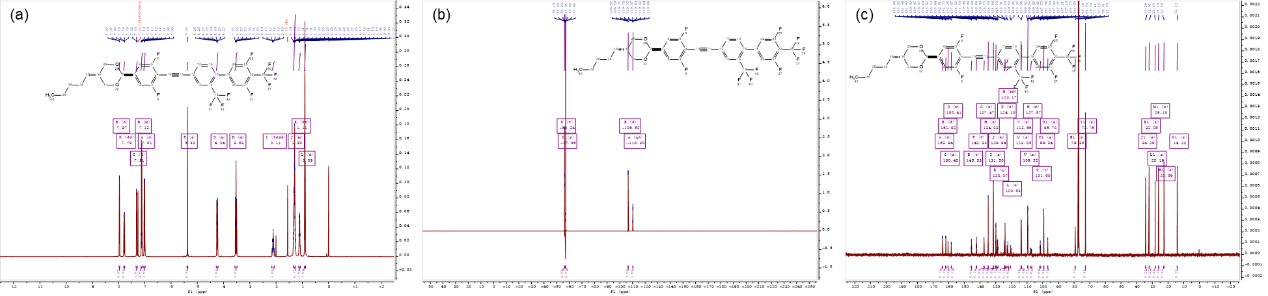


**Scheme S25**. (a) ^1^H, (b) ^19^F and (c) ^13^C NMR spectra of SCUT6c trans.

**SCUT6c** trans: ^1^H NMR (500 MHz, Chloroform-*d*) δ 7.97 (d, *J* = 1.6 Hz, 1H), 7.79 (dd, *J* = 7.9, 1.7 Hz, 1H), 7.31 (d, *J* = 8.0 Hz, 1H), 7.15 – 7.10 (m, 2H), 7.01 (d, *J* = 9.9 Hz, 2H), 5.38 (s, 1H), 4.28 – 4.21 (m, 2H), 3.57 – 3.48 (m, 2H), 2.11 (tddd, *J* = 11.4, 9.2, 7.0, 4.6 Hz, 1H), 1.35 – 1.26 (m, 6H), 1.11 (dt, *J* = 8.6, 6.5 Hz, 2H), 0.89 (d, *J* = 7.1 Hz, 3H). ^19^F NMR (471 MHz, Chloroform-*d*) δ -56.24 (t, *J* = 22.4 Hz), -57.06, -106.58 (d, *J* = 7.7 Hz), -110.38 (qd, *J* = 20.6, 9.1 Hz). ^13^C NMR (126 MHz, Chloroform-*d*) δ 163.94 (d, *J* = 5.4 Hz), 161.92 (d, *J* = 5.4 Hz), 160.56 – 160.39 (m), 158.50 – 158.33 (m), 145.53 (t, *J* = 10.7 Hz), 142.33 (t, *J* = 9.2 Hz), 137.47, 134.83, 131.50, 130.06 – 129.82 (m), 128.87 (q, *J* = 30.9 Hz), 124.10, 124.56 – 122.01 (m), 120.63, 113.85, 113.66, 109.70 – 109.34 (m), 108.01 – 106.92 (m), 101.60 (t, *J* = 19.8 Hz), 99.34, 96.78, 79.30, 72.76, 34.29, 32.05, 28.19, 26.10, 22.59, 14.12.


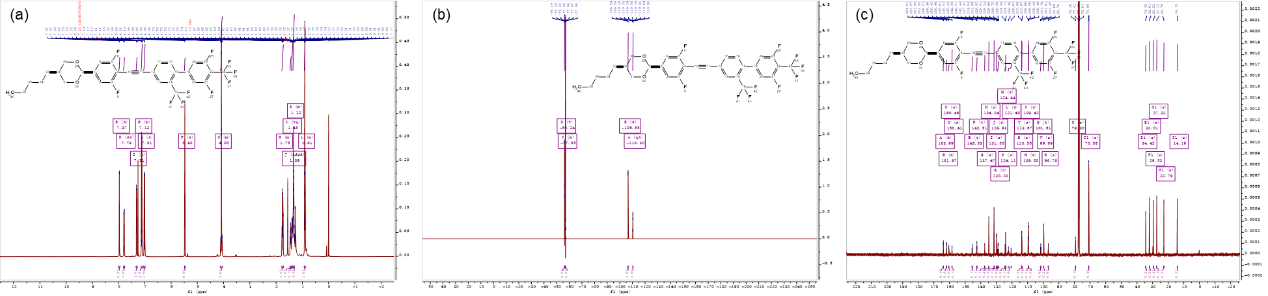


**Scheme S26**. (a) ^1^H, (b) ^19^F and (c) ^13^C NMR spectra of SCUT6c cis.

**SCUT6c** cis: ^1^H NMR (500 MHz, Chloroform-*d*) δ 7.97 (d, *J* = 1.6 Hz, 1H), 7.79 (dd, *J* = 7.9, 1.7 Hz, 1H), 7.31 (d, *J* = 8.0 Hz, 1H), 7.15 – 7.09 (m, 2H), 7.01 (d, *J* = 9.9 Hz, 2H), 5.48 (s, 1H), 4.13 – 4.04 (m, 4H), 1.78 – 1.72 (m, 2H), 1.45 (tq, *J* = 7.3, 2.1 Hz, 1H), 1.39 (dddd, *J* = 14.4, 7.1, 3.4, 1.7 Hz, 2H), 1.36 – 1.30 (m, 4H), 0.93 – 0.89 (m, 3H). ^19^F NMR (471 MHz, Chloroform-*d*) δ -56.24 (t, *J* = 22.3 Hz), -56.99 – -57.11 (m), -106.55 (d, *J* = 8.4 Hz), -110.38 (qd, *J* = 21.8, 21.4, 10.0 Hz). ^13^C NMR (126 MHz, Chloroform-*d*) δ 163.99 (d, *J* = 5.3 Hz), 161.97 (d, *J* = 5.1 Hz), 160.46, 158.51 – 158.32 (m), 145.53 (t, *J* = 10.7 Hz), 142.51 (t, *J* = 9.1 Hz), 137.47, 134.84, 131.50, 129.93 (d, *J* = 5.5 Hz), 128.88 (d, *J* = 31.0 Hz), 124.44, 124.11, 121.45 (d, *J* = 205.1 Hz), 113.86, 113.67, 109.58 (d, *J* = 4.8 Hz), 109.42 (d, *J* = 5.0 Hz), 101.61 (t, *J* = 19.8 Hz), 99.69, 96.76, 79.30, 70.86, 34.42, 32.01, 29.52, 27.32, 22.79, 14.19.


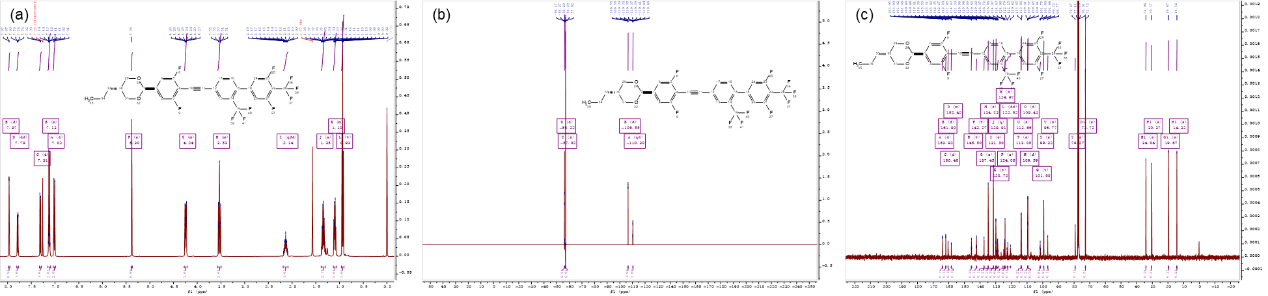


**Scheme S27**. (a) ^1^H, (b) ^19^F and (c) ^13^C NMR spectra of SCUT6d.

**SCUT6d**: ^1^H NMR (500 MHz, Chloroform-*d*) δ 7.97 (d, *J* = 1.5 Hz, 1H), 7.79 (dd, *J* = 8.0, 1.6 Hz, 1H), 7.31 (d, *J* = 7.9 Hz, 1H), 7.15 – 7.10 (m, 2H), 7.02 (d, *J* = 9.5 Hz, 2H), 5.38 (s, 1H), 4.27 – 4.21 (m, 2H), 3.57 – 3.50 (m, 2H), 2.14 (qdd, *J* = 11.5, 6.9, 4.6 Hz, 1H), 1.39 – 1.30 (m, 2H), 1.13 – 1.07 (m, 2H), 0.93 (t, *J* = 7.3 Hz, 3H). ^19^F NMR (471 MHz, Chloroform-*d*) δ -56.22 (d, *J* = 44.8 Hz), -57.02, -106.55 (d, *J* = 8.7 Hz), -110.38 (qd, *J* = 22.4, 10.3 Hz). ^13^C NMR (126 MHz, Chloroform-*d*) δ 163.92 (d, *J* = 5.3 Hz), 161.90 (d, *J* = 5.3 Hz), 160.46 (d, *J* = 4.2 Hz), 158.49 – 158.30 (m), 145.50 (t, *J* = 10.7 Hz), 142.27 (t, *J* = 9.3 Hz), 137.45, 134.83, 131.50, 129.91 (q, *J* = 5.3 Hz), 128.72 (t, *J* = 31.1 Hz), 124.97, 124.06, 122.52 (dd, *J* = 273.9, 205.0 Hz), 113.85, 113.66, 109.59 (d, *J* = 4.8 Hz), 109.43 (d, *J* = 4.8 Hz), 101.60 (t, *J* = 19.8 Hz), 99.32, 96.77, 79.27, 72.72, 34.04, 30.37, 19.67, 14.32.


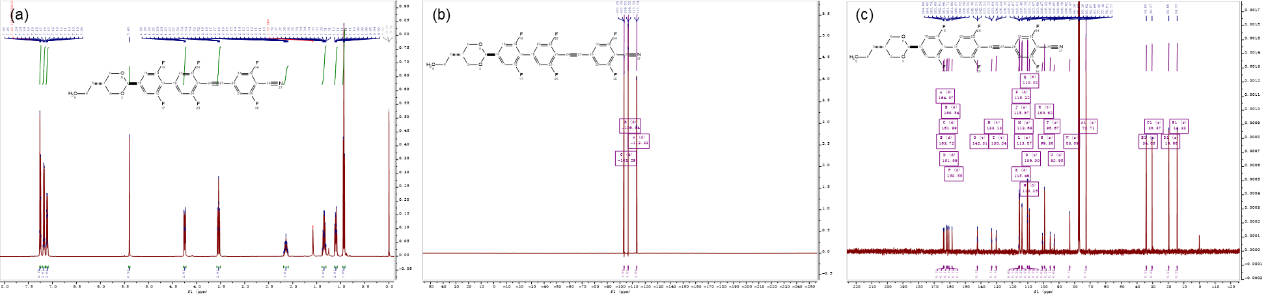


**Scheme S28**. (a) ^1^H, (b) ^19^F and (c) ^13^C NMR spectra of SCUT7a.

**SCUT7a**: ^1^H NMR (500 MHz, Chloroform-*d*) δ 7.26 – 7.24 (m, 2H), 7.20 – 7.15 (m, 2H), 7.11 (dt, *J* = 8.0, 1.3 Hz, 2H), 5.40 (s, 1H), 4.28 – 4.22 (m, 2H), 3.58 – 3.50 (m, 2H), 2.19 – 2.09 (m, 1H), 1.39 – 1.31 (m, 2H), 1.13 – 1.07 (m, 2H), 0.94 (t, *J* = 7.3 Hz, 3H). ^19^F NMR (471 MHz, Chloroform-*d*) δ -103.29 (d, *J* = 9.0 Hz), -106.54 (d, *J* = 8.5 Hz), -113.33 (d, *J* = 10.9 Hz). ^13^C NMR (126 MHz, Chloroform-*d*) δ 164.07 (d, *J* = 5.5 Hz), 163.72 (d, *J* = 5.8 Hz), 161.99 (d, *J* = 5.4 Hz), 161.69 (d, *J* = 5.8 Hz), 160.54 (d, *J* = 6.2 Hz), 158.55 (d, *J* = 6.6 Hz), 142.31 (t, *J* = 9.7 Hz), 133.13 (t, *J* = 10.6 Hz), 130.24 (t, *J* = 11.9 Hz), 115.67, 115.49 (d, *J* = 4.0 Hz), 115.32 (d, *J* = 3.9 Hz), 113.87, 113.69, 110.25 (d, *J* = 5.8 Hz), 110.08 (d, *J* = 5.5 Hz), 109.00, 100.62 (t, *J* = 19.5 Hz), 99.30, 95.67, 92.95 (d, *J* = 19.2 Hz), 83.09, 72.71, 34.05, 30.37, 19.66, 14.33.


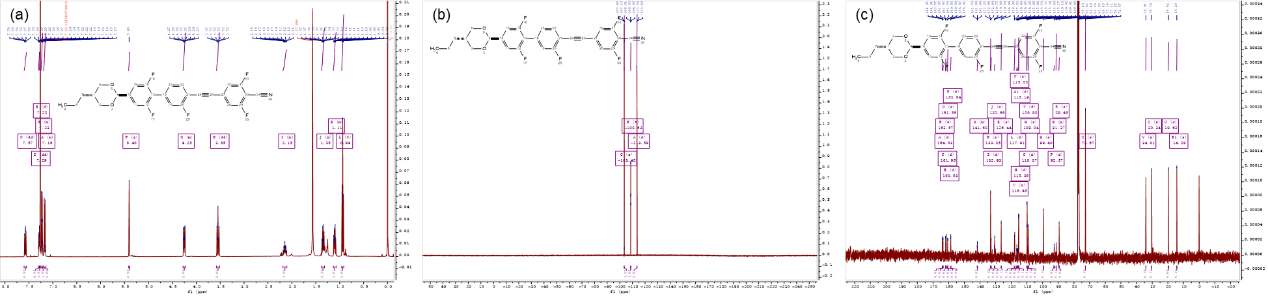


**Scheme S29**. (a) ^1^H, (b) ^19^F and (c) ^13^C NMR spectra of SCUT7b.

**SCUT7b**: ^1^H NMR (500 MHz, Chloroform-*d*) δ 7.57 (dd, *J* = 8.2, 7.2 Hz, 1H), 7.29 (dd, *J* = 5.5, 1.5 Hz, 1H), 7.28 (d, *J* = 1.4 Hz, 1H), 7.25 – 7.21 (m, 2H), 7.20 – 7.14 (m, 2H), 5.40 (s, 1H), 4.28 – 4.22 (m, 2H), 3.55 (dd, *J* = 12.1, 10.9 Hz, 2H), 2.19 – 2.10 (m, 1H), 1.38 – 1.32 (m, 2H), 1.13 – 1.08 (m, 2H), 0.94 (t, *J* = 7.3 Hz, 3H). ^19^F NMR (471 MHz, Chloroform-*d*) δ -103.48, -108.63 (t, *J* = 8.3 Hz), -113.59 (d, *J* = 8.4 Hz). ^13^C NMR (126 MHz, Chloroform-*d*) δ 164.03 (d, *J* = 5.2 Hz), 163.57, 161.95 (d, *J* = 5.3 Hz), 161.56, 160.63 (d, *J* = 7.2 Hz), 158.64 (d, *J* = 6.9 Hz), 141.85 – 141.51 (m), 133.25, 132.93 (d, *J* = 8.5 Hz), 130.66, 126.44, 117.91 (d, *J* = 22.0 Hz), 116.45, 115.88, 115.36 (d, *J* = 3.9 Hz), 115.19 (d, *J* = 3.7 Hz), 110.07 (d, *J* = 6.7 Hz), 109.90 (d, *J* = 3.6 Hz), 109.04, 99.40, 92.57 (d, *J* = 19.9 Hz), 91.47 – 91.27 (m), 89.45, 72.67, 34.01, 30.34, 19.62, 14.29.


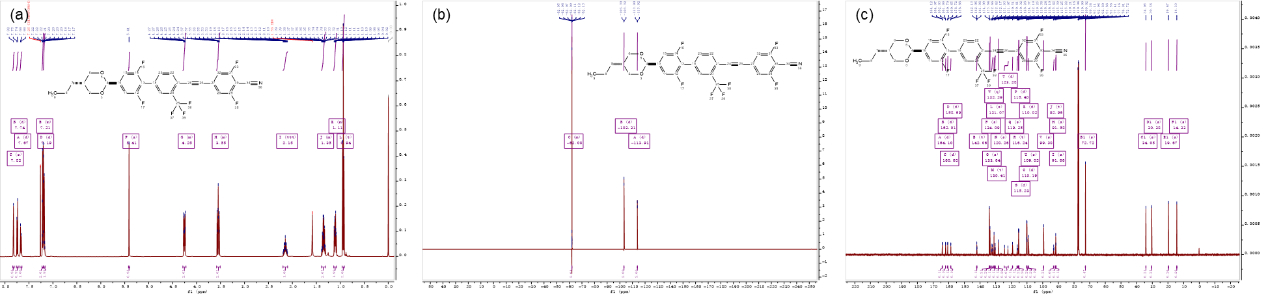


**Scheme S30**. (a) ^1^H, (b) ^19^F and (c) ^13^C NMR spectra of SCUT7c.

**SCUT7c**: ^1^H NMR (500 MHz, Chloroform-*d*) δ 7.82 (s, 1H), 7.74 (d, *J* = 8.0 Hz, 1H), 7.67 (d, *J* = 8.1 Hz, 1H), 7.23 – 7.20 (m, 2H), 7.19 (d, *J* = 8.2 Hz, 2H), 5.41 (s, 1H), 4.29 – 4.22 (m, 2H), 3.58 – 3.51 (m, 2H), 2.15 (ttt, *J* = 11.5, 7.0, 4.6 Hz, 1H), 1.39 – 1.31 (m, 2H), 1.14 – 1.08 (m, 2H), 0.94 (t, *J* = 7.3 Hz, 3H). ^19^F NMR (471 MHz, Chloroform-*d*) δ -62.03 – -62.16 (m), -103.31 (d, *J* = 8.4 Hz), -113.91 (d, *J* = 8.2 Hz). ^13^C NMR (126 MHz, Chloroform-*d*) δ 164.10 (d, *J* = 5.3 Hz), 162.01 (d, *J* = 5.4 Hz), 160.68 (d, *J* = 6.7 Hz), 158.69 (d, *J* = 6.7 Hz), 142.06 (t, *J* = 9.6 Hz), 134.09, 133.64, 132.29 (q, *J* = 30.9 Hz), 131.07, 130.41 (t, *J* = 11.9 Hz), 128.26, 123.28 (d, *J* = 273.6 Hz), 119.25, 116.24 (t, *J* = 17.8 Hz), 115.40 (d, *J* = 3.9 Hz), 115.23 (d, *J* = 3.9 Hz), 110.19 (d, *J* = 6.0 Hz), 110.02 (d, *J* = 6.0 Hz), 109.02, 99.38, 92.96 (t, *J* = 19.4 Hz), 91.66, 91.58, 72.72, 34.05, 30.38, 19.67, 14.33.


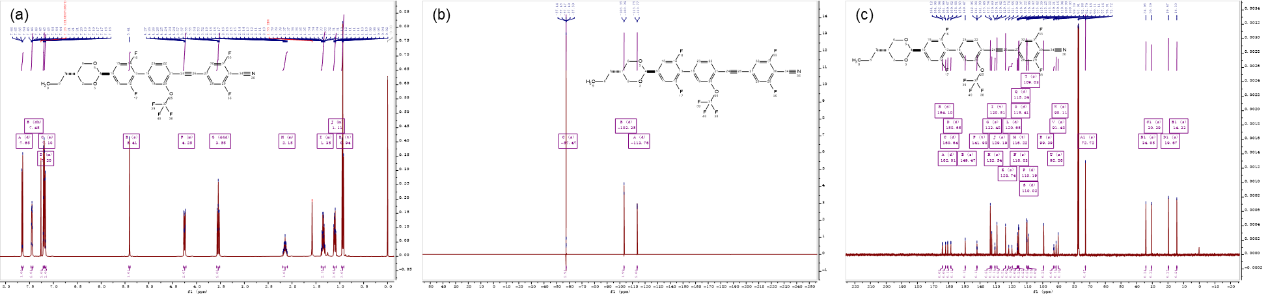


**Scheme S31**. (a) ^1^H, (b) ^19^F and (c) ^13^C NMR spectra of SCUT7d.

**SCUT7d**: ^1^H NMR (500 MHz, Chloroform-*d*) δ 7.65 (d, *J* = 8.4 Hz, 1H), 7.45 (dh, *J* = 4.7, 1.4 Hz, 2H), 7.22 – 7.19 (m, 2H), 7.19 – 7.15 (m, 2H), 5.41 (s, 1H), 4.28 – 4.22 (m, 2H), 3.55 (ddd, *J* = 11.7, 10.3, 1.6 Hz, 2H), 2.20 – 2.10 (m, 1H), 1.39 – 1.31 (m, 2H), 1.13 – 1.07 (m, 2H), 0.94 (t, *J* = 7.3 Hz, 3H). ^19^F NMR (471 MHz, Chloroform-*d*) δ -57.47, -103.35 (d, *J* = 8.2 Hz), -113.76 (d, *J* = 8.8 Hz). ^13^C NMR (126 MHz, Chloroform-*d*) δ 164.10 (d, *J* = 5.5 Hz), 162.01 (d, *J* = 5.4 Hz), 160.64 (d, *J* = 6.7 Hz), 158.65 (d, *J* = 6.6 Hz), 149.47, 141.98 (t, *J* = 9.6 Hz), 133.48, 132.54, 130.51 (t, *J* = 11.8 Hz), 129.19, 123.74, 120.65 (d, *J* = 259.5 Hz), 116.22 (t, *J* = 17.9 Hz), 116.03, 115.41 (d, *J* = 3.8 Hz), 115.24 (d, *J* = 3.7 Hz), 110.19 (d, *J* = 6.1 Hz), 110.02 (d, *J* = 5.9 Hz), 109.03, 99.39, 92.86, 91.48, 90.11, 72.72, 34.05, 30.39, 19.67, 14.33.


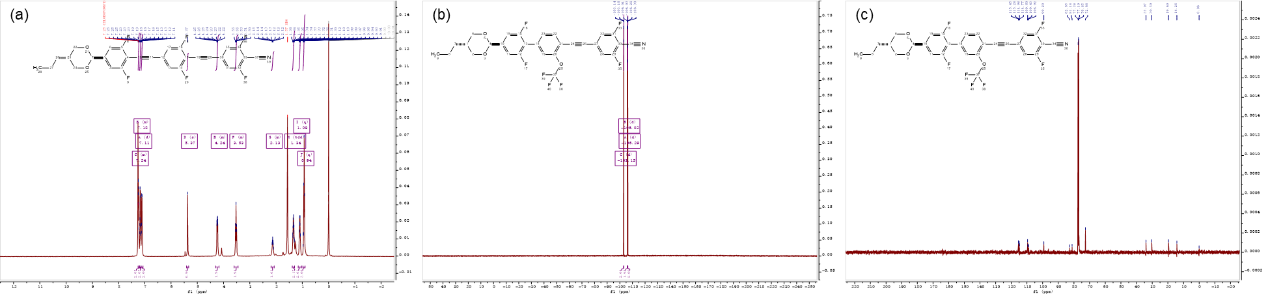


**Scheme S32**. (a) ^1^H, (b) ^19^F and (c) ^13^C NMR spectra of SCUT8a.

**SCUT8a**: ^1^H NMR (500 MHz, Chloroform-*d*) δ 7.26 – 7.21 (m, 2H), 7.21 – 7.14 (m, 2H), 7.11 (d, *J* = 7.8 Hz, 2H), 5.37 (s, 1H), 4.31 – 4.17 (m, 2H), 3.60 – 3.45 (m, 2H), 2.19 – 2.07 (m, 1H), 1.34 (tdd, *J* = 7.6, 5.6, 2.8 Hz, 2H), 1.09 (q, *J* = 7.0 Hz, 2H), 0.94 (q, *J* = 7.4, 7.0 Hz, 3H). ^19^F NMR (471 MHz, Chloroform-*d*) δ -103.15 (d, *J* = 8.5 Hz), -106.02 (d, *J* = 7.9 Hz), -106.29 (d, *J* = 8.6 Hz).


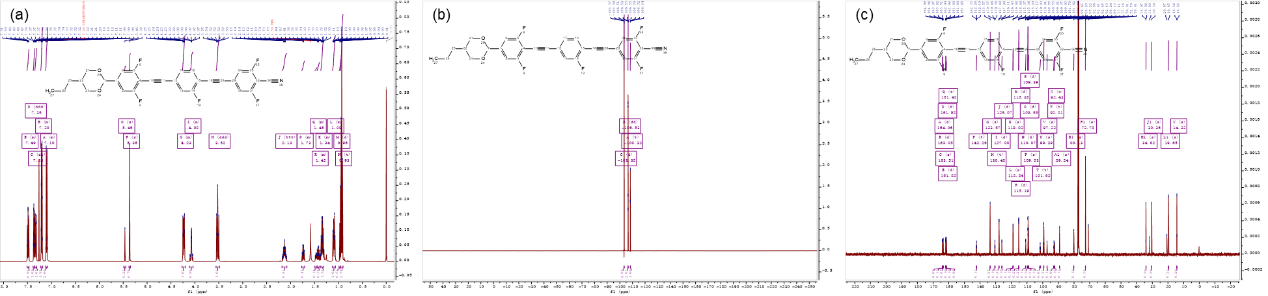


**Scheme S33**. (a) ^1^H, (b) ^19^F and (c) ^13^C NMR spectra of SCUT8b.

**SCUT8b** trans/cis=4/1: ^1^H NMR (500 MHz, Chloroform-*d*) δ 7.51 – 7.47 (m, 1H), 7.36 (ddd, *J* = 8.0, 2.4, 1.5 Hz, 1H), 7.33 (dt, *J* = 9.6, 2.0 Hz, 1H), 7.22 – 7.18 (m, 2H), 7.12 – 7.08 (m, 2H), 5.36 (s, 1H) [5.46 (s, 0H)], 4.27 – 4.20 (m, 2H), 3.52 (ddd, *J* = 11.7, 10.4, 1.5 Hz, 2H) [4.11 – 4.04 (m, 1H)]z, 2.13 (ttt, *J* = 11.5, 7.0, 4.6 Hz, 1H) [1.49 – 1.44 (m, 0H)], 1.37 – 1.31 (m, 2H) [1.76 – 1.71 (m, 0H)], 1.12 – 1.06 (m, 2H) [1.44 – 1.39 (m, 0H)], 0.93 (t, *J* = 7.3 Hz, 3H) [0.96 (d, *J* = 7.3 Hz, 1H)]. ^19^F NMR (471 MHz, Chloroform-*d*) δ -103.38 (d, *J* = 8.2 Hz), -106.53 (dd, *J* = 13.8, 8.3 Hz), -108.32 (t, *J* = 8.4 Hz). ^13^C NMR (126 MHz, Chloroform-*d*) δ 164.06 (d, *J* = 5.5 Hz), 163.85 (d, *J* = 5.6 Hz), 163.51, 161.98 (d, *J* = 5.4 Hz), 161.82 (d, *J* = 5.4 Hz), 161.48, 142.26 (t, *J* = 9.2 Hz), 133.57, 130.48 (t, *J* = 12.0 Hz), 127.88 (d, *J* = 3.4 Hz), 126.07 (d, *J* = 9.5 Hz), 119.02, 118.84, 115.36 (d, *J* = 3.8 Hz), 115.19 (d, *J* = 3.7 Hz), 110.87 (d, *J* = 15.9 Hz), 109.55 (d, *J* = 4.5 Hz), 109.39 (d, *J* = 5.0 Hz), 109.03, 101.62 (t, *J* = 19.9 Hz), 99.29 [99.65], 97.22, 92.81 (t, *J* = 19.4 Hz), 92.43, 89.24, 80.13, 72.70 [70.81], 34.02 [34.07], 30.36 [31.69], 19.65 [20.67], 14.22 [14.32].


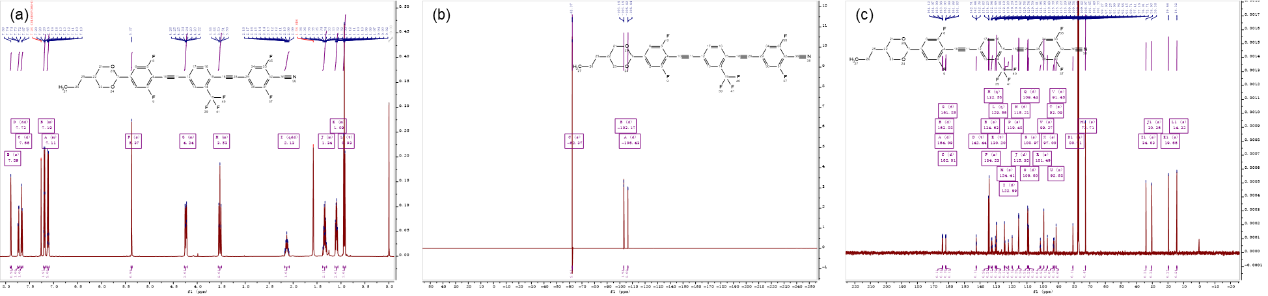


**Scheme S34**. (a) ^1^H, (b) ^19^F and (c) ^13^C NMR spectra of SCUT8c.

**SCUT8c**: ^1^H NMR (500 MHz, Chloroform-*d*) δ 7.89 (s, 1H), 7.73 (dd, *J* = 8.1, 1.6 Hz, 1H), 7.66 (d, *J* = 8.0 Hz, 1H), 7.21 – 7.17 (m, 2H), 7.14 – 7.09 (m, 2H), 5.37 (s, 1H), 4.27 – 4.21 (m, 2H), 3.56 – 3.49 (m, 2H), 2.13 (qdd, *J* = 11.4, 7.0, 4.6 Hz, 1H), 1.38 – 1.30 (m, 2H), 1.13 – 1.06 (m, 2H), 0.93 (t, *J* = 7.3 Hz, 3H). ^19^F NMR (471 MHz, Chloroform-*d*) δ -62.37, -103.17 (d, *J* = 8.4 Hz), -106.43 (d, *J* = 8.3 Hz). ^13^C NMR (126 MHz, Chloroform-*d*) δ 164.09 (d, *J* = 5.5 Hz), 163.88 (d, *J* = 5.3 Hz), 162.01 (d, *J* = 5.4 Hz), 161.85 (d, *J* = 5.5 Hz), 142.44 (t, *J* = 9.1 Hz), 134.63, 134.23, 132.56 (q, *J* = 31.1 Hz), 130.20 (t, *J* = 11.7 Hz), 129.56 (q, *J* = 5.1 Hz), 124.41, 122.99 (d, *J* = 273.9 Hz), 119.48, 115.38 (d, *J* = 3.9 Hz), 115.21 (d, *J* = 3.8 Hz), 109.60 (d, *J* = 4.7 Hz), 109.43 (d, *J* = 4.6 Hz), 108.97, 101.49, 99.27, 97.00, 93.09, 92.68, 91.45, 80.71, 72.71, 34.03, 30.36, 19.66, 14.32.


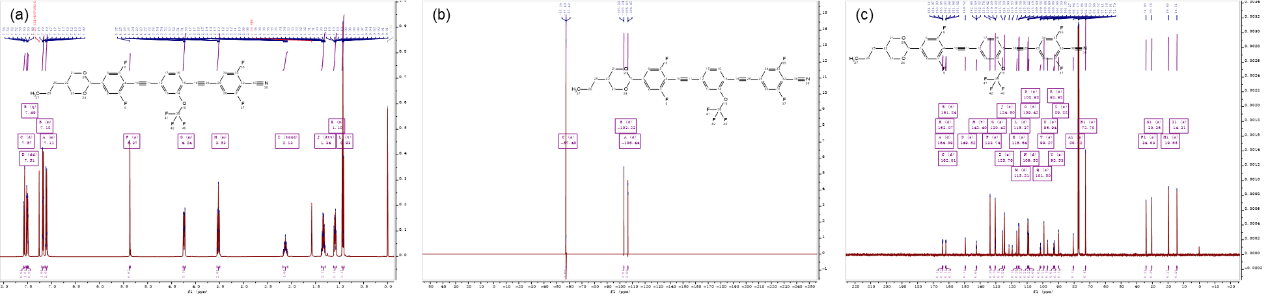


**Scheme S35**. (a) ^1^H, (b) ^19^F and (c) ^13^C NMR spectra of SCUT8d.

**SCUT8d**: ^1^H NMR (500 MHz, Chloroform-*d*) δ 7.57 (d, *J* = 7.9 Hz, 1H), 7.51 (dd, *J* = 8.0, 1.5 Hz, 1H), 7.49 (q, *J* = 1.4 Hz, 1H), 7.21 – 7.15 (m, 2H), 7.13 – 7.09 (m, 2H), 5.37 (s, 1H), 4.26 – 4.21 (m, 2H), 3.55 – 3.49 (m, 2H), 2.13 (tddd, *J* = 11.4, 9.1, 7.0, 4.6 Hz, 1H), 1.34 (dtt, *J* = 10.1, 7.7, 3.9 Hz, 2H), 1.12 – 1.06 (m, 2H), 0.93 (t, *J* = 7.3 Hz, 3H). ^19^F NMR (471 MHz, Chloroform-*d*) δ -57.40, -103.23 (d, *J* = 8.5 Hz), -106.44 (d, *J* = 8.8 Hz). ^13^C NMR (126 MHz, Chloroform-*d*) δ 164.09 (d, *J* = 5.4 Hz), 163.87 (d, *J* = 5.4 Hz), 162.01 (d, *J* = 5.4 Hz), 161.84 (d, *J* = 5.3 Hz), 149.52, 142.40 (t, *J* = 9.3 Hz), 133.74, 130.42, 125.76, 124.50, 116.64, 115.37 (d, *J* = 3.8 Hz), 115.21 (d, *J* = 3.8 Hz), 109.58 (d, *J* = 4.9 Hz), 109.42 (d, *J* = 4.8 Hz), 108.98, 101.50, 99.27, 96.94, 92.98, 92.53, 89.88, 80.52, 72.70, 34.03, 30.36, 19.65, 14.31.


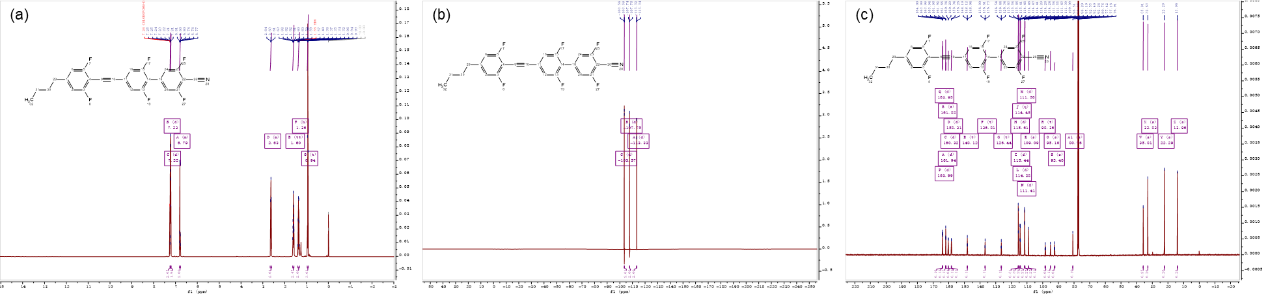


**Scheme S36**. (a) ^1^H, (b) ^19^F and (c) ^13^C NMR spectra of SCUT9a.

**SCUT9a**: ^1^H NMR (500 MHz, Chloroform-*d*) δ 7.23 (d, *J* = 3.7 Hz, 2H), 7.22 (d, *J* = 4.0 Hz, 2H), 6.82 – 6.77 (m, 2H), 2.66 – 2.59 (m, 2H), 1.60 (tt, *J* = 8.8, 6.9 Hz, 2H), 1.36 (h, *J* = 7.4 Hz, 2H), 0.94 (t, *J* = 7.4 Hz, 3H). ^19^F NMR (471 MHz, Chloroform-*d*) δ -103.57 (d, *J* = 8.8 Hz), -107.75 (d, *J* = 9.3 Hz), -113.33 (d, *J* = 9.2 Hz). ^13^C NMR (126 MHz, Chloroform-*d*) δ 163.99 (d, *J* = 1.8 Hz), 163.95 (d, *J* = 3.2 Hz), 161.94 (d, *J* = 5.4 Hz), 161.88, 160.32 (d, *J* = 7.2 Hz), 158.31 (d, *J* = 7.1 Hz), 148.12 (t, *J* = 9.0 Hz), 136.81 (t, *J* = 10.6 Hz), 126.44 (t, *J* = 12.6 Hz), 115.61 (d, *J* = 5.9 Hz), 115.44 (d, *J* = 6.0 Hz), 114.45 (q, *J* = 3.2 Hz), 114.28 (d, *J* = 3.7 Hz), 111.56 (d, *J* = 4.0 Hz), 111.41 (d, *J* = 4.0 Hz), 109.09, 98.36 (t, *J* = 19.8 Hz), 95.10, 92.40, 80.76, 35.81, 32.83, 22.29, 13.96.


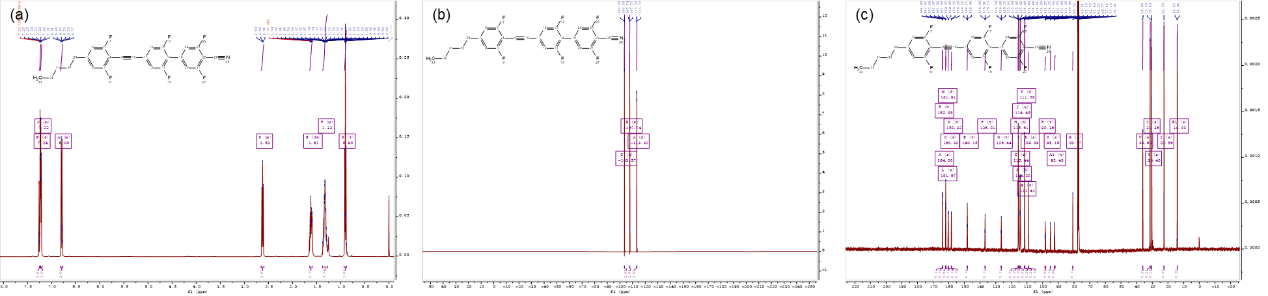


**Scheme S37**. (a) ^1^H, (b) ^19^F and (c) ^13^C NMR spectra of SCUT9b.

**SCUT9b**: ^1^H NMR (500 MHz, Chloroform-*d*) δ 7.24 (d, *J* = 4.3 Hz, 2H), 7.23 – 7.20 (m, 2H), 6.82 – 6.76 (m, 2H), 2.65 – 2.59 (m, 2H), 1.61 (dd, *J* = 14.2, 6.5 Hz, 2H), 1.38 – 1.27 (m, 4H), 0.90 (t, *J* = 6.9 Hz, 3H). ^19^F NMR (471 MHz, Chloroform-*d*) δ -103.57 (d, *J* = 8.8 Hz), -107.74, -113.32 (d, *J* = 8.5 Hz). ^13^C NMR (126 MHz, Chloroform-*d*) δ 164.00, 163.95 (d, *J* = 2.9 Hz), 161.97, 161.91 (d, *J* = 4.5 Hz), 160.32 (d, *J* = 7.2 Hz), 158.32 (d, *J* = 7.3 Hz), 148.15 (t, *J* = 9.1 Hz), 136.81 (t, *J* = 10.6 Hz), 126.44 (t, *J* = 12.5 Hz), 115.61 (d, *J* = 5.9 Hz), 115.44 (d, *J* = 5.8 Hz), 114.45 (q, *J* = 3.2 Hz), 114.28 (d, *J* = 3.3 Hz), 111.56 (d, *J* = 4.0 Hz), 111.41 (d, *J* = 4.0 Hz), 109.09, 98.36 (t, *J* = 19.7 Hz), 95.10, 92.40 (t, *J* = 19.2 Hz), 80.77, 36.08, 31.36, 30.40, 22.56, 14.08.


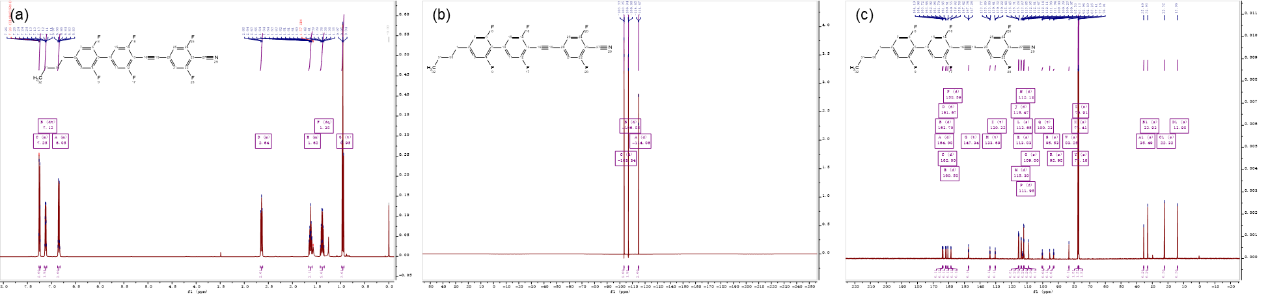


**Scheme S38**. (a) ^1^H, (b) ^19^F and (c) ^13^C NMR spectra of SCUT10a.

**SCUT10a**: ^1^H NMR (500 MHz, Chloroform-*d*) δ 7.26 – 7.23 (m, 2H), 7.12 (dt, *J* = 8.2, 1.3 Hz, 2H), 6.87 – 6.82 (m, 2H), 2.67 – 2.62 (m, 2H), 1.66 – 1.59 (m, 2H), 1.38 (dq, *J* = 14.7, 7.4 Hz, 2H), 0.95 (t, *J* = 7.4 Hz, 3H). ^19^F NMR (471 MHz, Chloroform-*d*) δ -103.34 (d, *J* = 8.5 Hz), -106.85 (d, *J* = 8.5 Hz), -114.86 (d, *J* = 9.3 Hz). ^13^C NMR (126 MHz, Chloroform-*d*) δ 164.08 (d, *J* = 5.4 Hz), 163.70 (d, *J* = 5.8 Hz), 162.00 (d, *J* = 5.4 Hz), 161.67 (d, *J* = 5.9 Hz), 160.58 (d, *J* = 7.1 Hz), 158.59 (d, *J* = 6.9 Hz), 147.34 (t, *J* = 9.6 Hz), 133.69 (t, *J* = 10.8 Hz), 130.32 (t, *J* = 11.9 Hz), 115.47 (d, *J* = 3.8 Hz), 115.30 (d, *J* = 3.7 Hz), 113.83, 113.65, 112.13 (d, *J* = 4.8 Hz), 111.96 (d, *J* = 4.8 Hz), 109.00, 100.23 (t, *J* = 19.6 Hz), 95.53, 92.98, 83.25, 77.41, 77.16, 76.91, 35.49, 32.93, 22.32, 13.98.


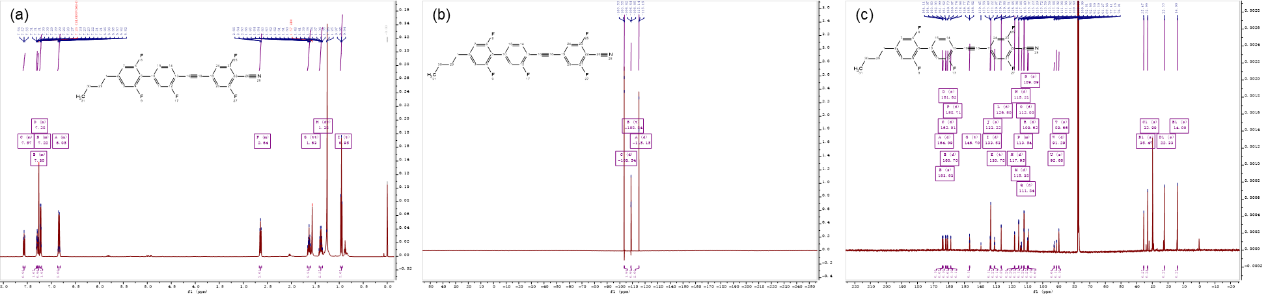


**Scheme S39**. (a) ^1^H, (b) ^19^F and (c) ^13^C NMR spectra of SCUT10b.

**SCUT10b**: ^1^H NMR (500 MHz, Chloroform-*d*) δ 7.59 – 7.55 (m, 1H), 7.31 – 7.29 (m, 1H), 7.29 – 7.27 (m, 1H), 7.25 – 7.20 (m, 2H), 6.87 – 6.81 (m, 2H), 2.67 – 2.62 (m, 2H), 1.63 (tt, *J* = 9.0, 6.8 Hz, 2H), 1.38 (dt, *J* = 14.7, 7.4 Hz, 2H), 0.95 (t, *J* = 7.3 Hz, 3H). ^19^F NMR (471 MHz, Chloroform-*d*) δ -103.54 (d, *J* = 8.4 Hz), -108.84 (t, *J* = 9.5 Hz), -115.15 (d, *J* = 10.9 Hz). ^13^C NMR (126 MHz, Chloroform-*d*) δ 164.09 (d, *J* = 5.5 Hz), 163.63, 162.01 (d, *J* = 5.4 Hz), 161.62, 160.70 (d, *J* = 7.3 Hz), 158.71 (d, *J* = 7.4 Hz), 146.70 (t, *J* = 9.4 Hz), 133.53 (d, *J* = 8.6 Hz), 133.22, 130.78 (t, *J* = 11.9 Hz), 126.50 (d, *J* = 3.3 Hz), 117.95 (d, *J* = 21.7 Hz), 115.38 (d, *J* = 3.8 Hz), 115.21 (d, *J* = 3.7 Hz), 113.67 – 113.37 (m), 112.00 (d, *J* = 5.0 Hz), 111.84 (d, *J* = 5.4 Hz), 109.62 (d, *J* = 15.6 Hz), 109.09, 92.66, 91.29 (d, *J* = 3.7 Hz), 89.66, 35.47, 32.99, 22.33, 14.00.


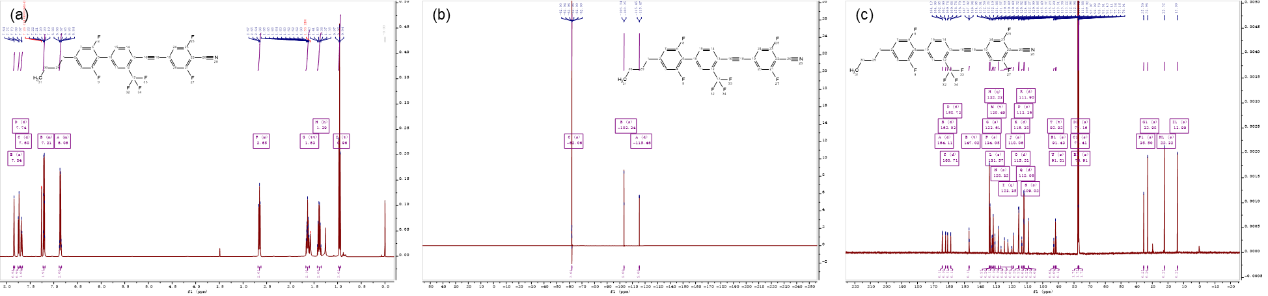


**Scheme S40**. (a) ^1^H, (b) ^19^F and (c) ^13^C NMR spectra of SCUT10c.

**SCUT10c**: ^1^H NMR (500 MHz, Chloroform-*d*) δ 7.84 (s, 1H), 7.74 (d, *J* = 8.0 Hz, 1H), 7.68 (d, *J* = 8.0 Hz, 1H), 7.23 – 7.18 (m, 2H), 6.89 – 6.84 (m, 2H), 2.68 – 2.62 (m, 2H), 1.63 (tt, *J* = 8.9, 6.9 Hz, 2H), 1.39 (h, *J* = 7.4 Hz, 2H), 0.96 (t, *J* = 7.3 Hz, 3H). ^19^F NMR (471 MHz, Chloroform-*d*) δ -62.06, -103.34, -115.46 (d, *J* = 10.4 Hz). ^13^C NMR (126 MHz, Chloroform-*d*) δ 164.11 (d, *J* = 5.3 Hz), 162.03 (d, *J* = 5.4 Hz), 160.71 (d, *J* = 7.3 Hz), 158.73 (d, *J* = 7.3 Hz), 147.02 (t, *J* = 9.5 Hz), 134.05, 133.61, 132.23 (q, *J* = 31.0 Hz), 131.57, 130.49 (t, *J* = 11.9 Hz), 128.32, 123.35 (q, *J* = 273.7 Hz), 118.86, 115.38 (d, *J* = 3.9 Hz), 115.21 (d, *J* = 3.8 Hz), 113.36, 112.06 (d, *J* = 5.3 Hz), 111.90 (d, *J* = 5.1 Hz), 109.02, 92.92 (t, *J* = 19.5 Hz), 91.81, 91.43, 77.41, 77.16, 76.91, 35.50, 32.98, 22.32, 13.99.


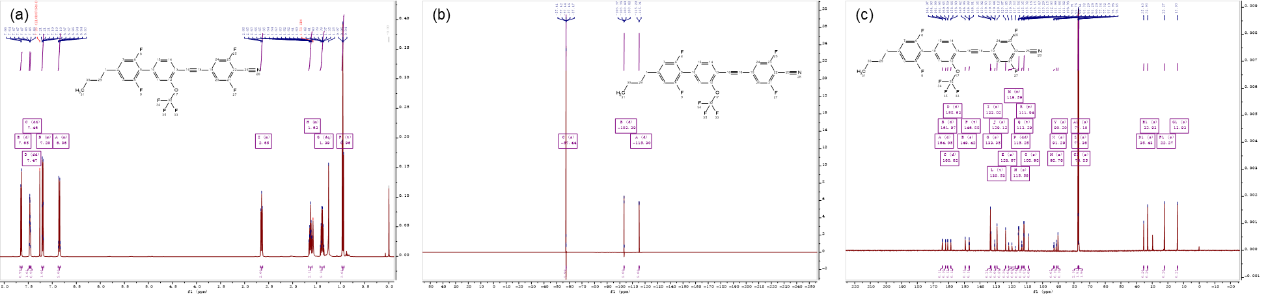


**Scheme S41**. (a) ^1^H, (b) ^19^F and (c) ^13^C NMR spectra of SCUT10d.

**SCUT10d**: ^1^H NMR (500 MHz, Chloroform-*d*) δ 7.65 (d, *J* = 8.4 Hz, 1H), 7.47 (dd, *J* = 2.5, 1.3 Hz, 1H), 7.46 (dd, *J* = 3.2, 1.5 Hz, 1H), 7.22 – 7.18 (m, 2H), 6.88 – 6.82 (m, 2H), 2.67 – 2.62 (m, 2H), 1.67 – 1.60 (m, 2H), 1.39 (dq, *J* = 14.7, 7.4 Hz, 2H), 0.96 (t, *J* = 7.3 Hz, 3H). ^19^F NMR (471 MHz, Chloroform-*d*) δ -57.44, -103.39 (d, *J* = 8.9 Hz), -115.30 (d, *J* = 9.0 Hz). ^19^F NMR (126 MHz, Chloroform-*d*) δ 164.05 (d, *J* = 5.5 Hz), 161.97 (d, *J* = 5.5 Hz), 160.62 (d, *J* = 7.3 Hz), 158.63 (d, *J* = 7.2 Hz), 149.42, 146.88 (t, *J* = 9.6 Hz), 133.35, 133.02, 130.53 (t, *J* = 11.9 Hz), 129.12, 123.67, 121.82 – 117.35 (m), 115.56, 115.25 (dd, *J* = 21.1, 3.7 Hz), 113.29 (t, *J* = 17.9 Hz), 112.10 – 111.75 (m), 108.98, 92.76, 91.29, 90.20, 77.36, 77.10, 76.85, 35.43, 32.93, 22.27, 13.93.


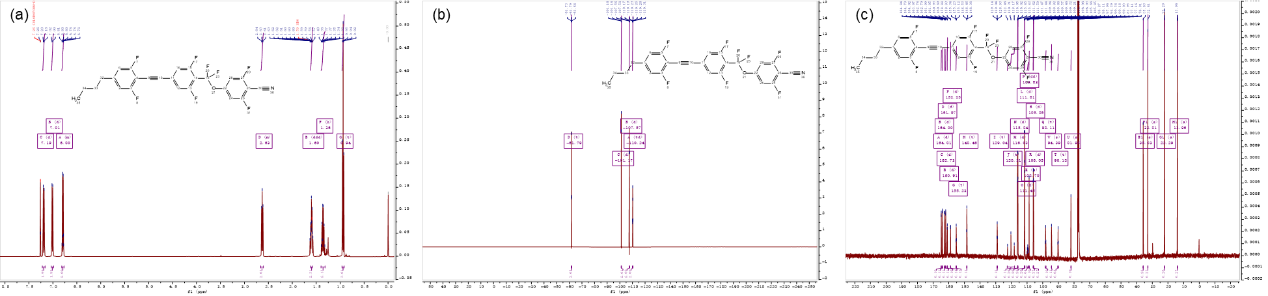


**Scheme S42**. (a) ^1^H, (b) ^19^F and (c) ^13^C NMR spectra of SCUT11a.

**SCUT11a**: ^1^H NMR (500 MHz, Chloroform-*d*) δ 7.19 (d, *J* = 9.3 Hz, 2H), 7.01 (d, *J* = 8.2 Hz, 2H), 6.81 – 6.77 (m, 2H), 2.66 – 2.60 (m, 2H), 1.60 (ddd, *J* = 9.5, 4.5, 1.9 Hz, 2H), 1.36 (h, *J* = 7.4 Hz, 2H), 0.94 (t, *J* = 7.3 Hz, 3H). ^19^F NMR (471 MHz, Chloroform-*d*) δ -61.79 (t, *J* = 26.5 Hz), -101.17 (d, *J* = 8.8 Hz), -107.57 (d, *J* = 9.4 Hz), -110.24 (td, *J* = 26.9, 25.8, 11.4 Hz). ^13^C NMR (126 MHz, Chloroform-*d*) δ 164.81 (d, *J* = 6.6 Hz), 164.00 (d, *J* = 5.6 Hz), 162.73 (d, *J* = 6.6 Hz), 161.97 (d, *J* = 5.6 Hz), 160.91 (d, *J* = 5.6 Hz), 158.85 (d, *J* = 5.7 Hz), 155.23 (t, *J* = 13.5 Hz), 148.46 (t, *J* = 9.1 Hz), 129.04 (t, *J* = 12.9 Hz), 120.31 (t, *J* = 269.5 Hz), 116.03 (d, *J* = 4.0 Hz), 115.84 (d, *J* = 3.6 Hz), 111.61 (d, *J* = 3.9 Hz), 111.45 (d, *J* = 3.8 Hz), 109.63 (dd, *J* = 30.2, 16.3 Hz), 108.70, 106.05 (d, *J* = 3.8 Hz), 105.86 (d, *J* = 4.0 Hz), 98.11 (t, *J* = 19.7 Hz), 94.39, 90.18 (t, *J* = 19.4 Hz), 81.95, 35.83, 32.81, 22.29, 13.96.


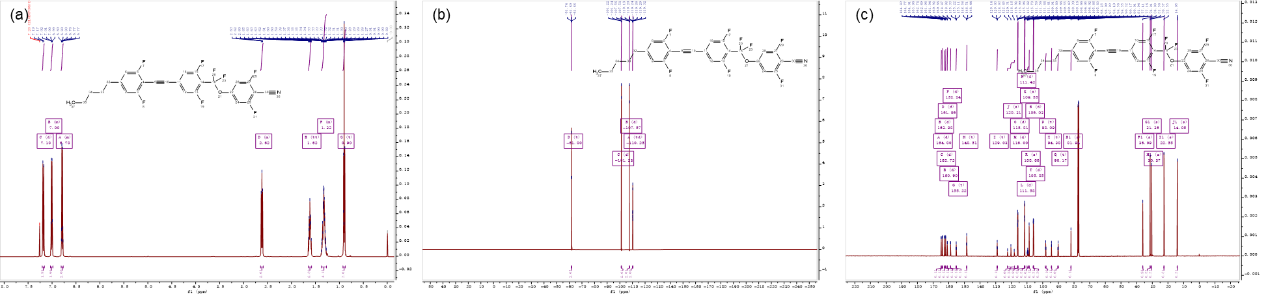


**Scheme S43**. (a) ^1^H, (b) ^19^F and (c) ^13^C NMR spectra of SCUT11b.

**SCUT11b**: ^1^H NMR (500 MHz, Chloroform-*d*) δ 7.18 (d, *J* = 9.3 Hz, 2H), 7.03 – 6.98 (m, 2H), 6.81 – 6.77 (m, 2H), 2.65 – 2.59 (m, 2H), 1.62 (tt, *J* = 9.2, 6.8 Hz, 2H), 1.37 – 1.27 (m, 4H), 0.90 (t, *J* = 7.0 Hz, 3H). ^19^F NMR (471 MHz, Chloroform-*d*) δ -61.80 (t, *J* = 26.6 Hz), -101.23 (d, *J* = 8.3 Hz), -107.57 (d, *J* = 8.4 Hz), -110.25 (td, *J* = 27.5, 11.0 Hz). ^13^C NMR (126 MHz, Chloroform-*d*) δ 164.80 (d, *J* = 6.7 Hz), 163.98 (d, *J* = 5.8 Hz), 162.72 (d, *J* = 6.6 Hz), 161.96 (d, *J* = 5.7 Hz), 160.90 (d, *J* = 5.9 Hz), 158.84 (d, *J* = 5.9 Hz), 155.22 (t, *J* = 13.5 Hz), 148.51 (t, *J* = 9.1 Hz), 129.03 (t, *J* = 12.9 Hz), 120.31, 116.00 (d, *J* = 3.8 Hz), 115.81 (d, *J* = 3.4 Hz), 111.58 (d, *J* = 4.1 Hz), 111.42 (d, *J* = 4.0 Hz), 109.55, 108.66, 106.03 (d, *J* = 3.8 Hz), 105.85 (d, *J* = 3.6 Hz), 98.09 (t, *J* = 19.7 Hz), 94.38 (t, *J* = 3.2 Hz), 90.17 (t, *J* = 19.4 Hz), 81.94, 36.09, 31.36, 30.37, 22.55, 14.05.


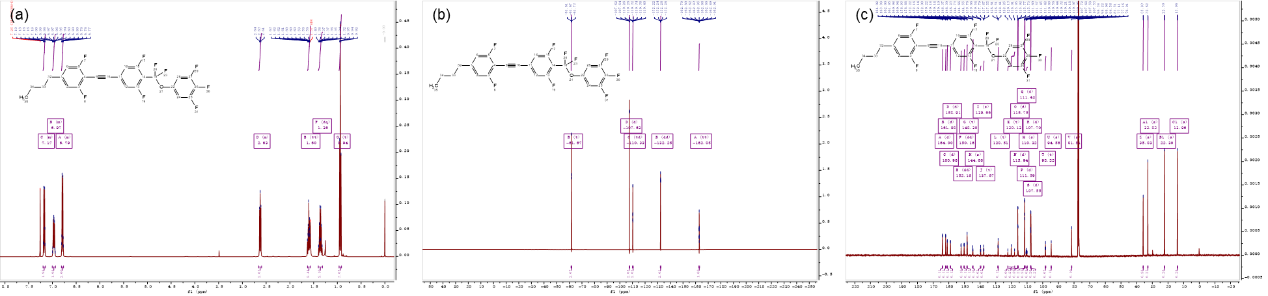


**Scheme S44**. (a) ^1^H, (b) ^19^F and (c) ^13^C NMR spectra of SCUT11c.

**SCUT11c**: ^1^H NMR (500 MHz, Chloroform-*d*) δ 7.20 – 7.15 (m, 2H), 7.00 – 6.94 (m, 2H), 6.81 – 6.77 (m, 2H), 2.66 – 2.60 (m, 2H), 1.60 (tt, *J* = 7.0, 1.7 Hz, 2H), 1.36 (dq, *J* = 14.7, 7.4 Hz, 2H), 0.94 (t, *J* = 7.3 Hz, 3H). ^19^F NMR (471 MHz, Chloroform-*d*) δ -61.67 (t, *J* = 26.7 Hz), -107.63 (d, *J* = 9.0 Hz), -110.33 (td, *J* = 26.0, 9.5 Hz), -132.25 (dd, *J* = 19.9, 8.5 Hz), -162.85 (tt, *J* = 22.6, 6.7 Hz). ^13^C NMR (126 MHz, Chloroform-*d*) δ 164.00 (d, *J* = 5.8 Hz), 161.98 (d, *J* = 5.8 Hz), 160.96 (d, *J* = 6.2 Hz), 158.91 (d, *J* = 6.1 Hz), 152.16 (dd, *J* = 10.6, 5.3 Hz), 150.16 (dd, *J* = 10.7, 5.1 Hz), 148.30 (t, *J* = 9.1 Hz), 144.66, 139.66, 137.67 (t, *J* = 15.2 Hz), 128.51 (t, *J* = 12.7 Hz), 120.13 (t, *J* = 266.6 Hz), 115.94 (d, *J* = 4.4 Hz), 115.75 (d, *J* = 4.4 Hz), 111.59 (d, *J* = 3.9 Hz), 111.43 (d, *J* = 3.8 Hz), 110.32, 107.70 (d, *J* = 5.9 Hz), 107.55 (d, *J* = 5.9 Hz), 98.22 (t, *J* = 19.7 Hz), 94.56, 81.51, 35.83, 32.82, 22.30, 13.96.


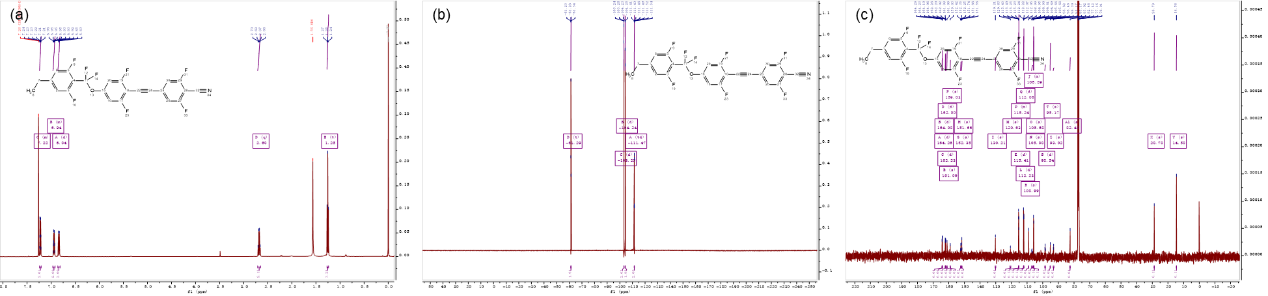


**Scheme S45**. (a) ^1^H, (b) ^19^F and (c) ^13^C NMR spectra of SCUT12a.

**SCUT12a**: ^1^H NMR (500 MHz, Chloroform-*d*) δ 7.24 – 7.21 (m, 2H), 6.96 – 6.92 (m, 2H), 6.84 (d, *J* = 10.8 Hz, 2H), 2.68 (q, *J* = 7.6 Hz, 2H), 1.25 (t, *J* = 7.6 Hz, 3H). ^19^F NMR (471 MHz, Chloroform-*d*) δ -61.29 (t, *J* = 26.2 Hz), -103.25 (d, *J* = 8.4 Hz), -104.24 (d, *J* = 8.4 Hz), -111.47 (td, *J* = 25.3, 10.9 Hz). ^13^C NMR (126 MHz, Chloroform-*d*) δ 164.26 (d, *J* = 7.3 Hz), 164.08 (d, *J* = 5.1 Hz), 162.23 (d, *J* = 7.1 Hz), 162.00 (d, *J* = 5.2 Hz), 161.09, 159.03, 152.35, 151.66, 130.21, 126.62, 120.63, 115.41 (d, *J* = 4.0 Hz), 115.32 – 115.20 (m), 112.23 (d, *J* = 3.4 Hz), 112.06 (d, *J* = 2.7 Hz), 108.99, 105.88, 105.68, 98.54 (d, *J* = 20.0 Hz), 95.17, 93.02, 82.48, 28.78, 14.58.


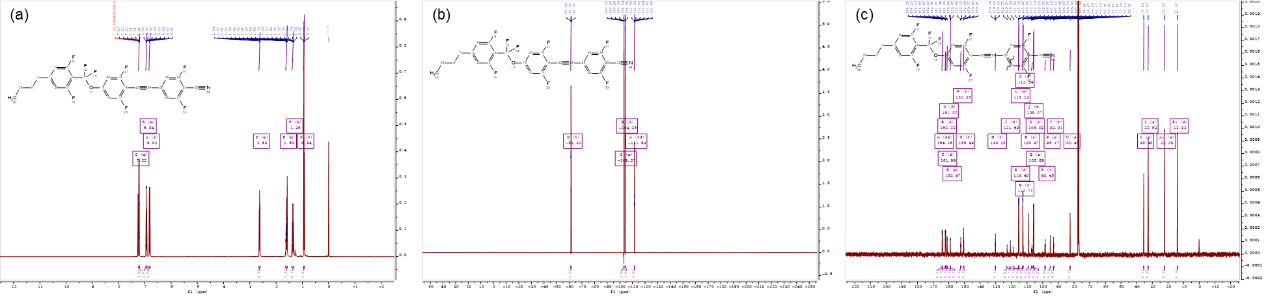


**Scheme S46**. (a) ^1^H, (b) ^19^F and (c) ^13^C NMR spectra of SCUT12b.

**SCUT12b**: ^1^H NMR (500 MHz, Chloroform-*d*) δ 7.24 – 7.20 (m, 2H), 6.96 – 6.91 (m, 2H), 6.82 (d, *J* = 10.6 Hz, 2H), 2.66 – 2.60 (m, 2H), 1.63 – 1.57 (m, 2H), 1.36 (h, *J* = 7.4 Hz, 2H), 0.94 (t, *J* = 7.4 Hz, 3H). ^19^F NMR (471 MHz, Chloroform-*d*) δ -61.32 (t, *J* = 26.4 Hz), -103.18 – -103.39 (m), -104.25 (d, *J* = 8.6 Hz), -111.63 (td, *J* = 25.5, 11.4 Hz). ^13^C NMR (126 MHz, Chloroform-*d*) δ 164.16 (dd, *J* = 22.8, 6.3 Hz), 162.22 (d, *J* = 7.1 Hz), 161.99 (d, *J* = 5.4 Hz), 161.02 (d, *J* = 5.3 Hz), 159.08 – 158.87 (m), 152.35 (t, *J* = 13.8 Hz), 150.49 (t, *J* = 10.0 Hz), 130.20 (t, *J* = 11.8 Hz), 121.69 (d, *J* = 266.8 Hz), 115.40 (d, *J* = 4.0 Hz), 115.23 (d, *J* = 4.0 Hz), 112.71 (d, *J* = 3.4 Hz), 112.54 (d, *J* = 3.3 Hz), 108.97, 107.26 – 106.53 (m), 105.89, 105.69, 98.45 (t, *J* = 19.9 Hz), 95.17, 93.01 (t, *J* = 19.4 Hz), 82.47, 35.48, 32.63, 22.28, 13.93.


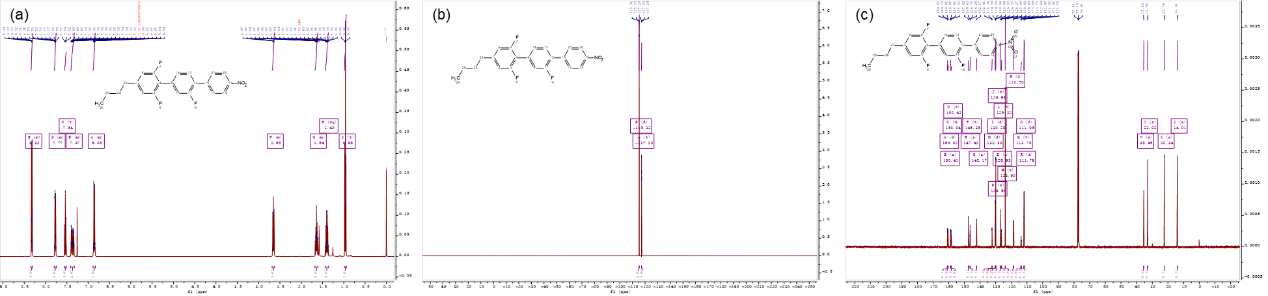


**Scheme S47**. (a) ^1^H, (b) ^19^F and (c) ^13^C NMR spectra of SCUT13a.

**SCUT13a**: ^1^H NMR (500 MHz, Chloroform-*d*) δ 8.35 – 8.30 (m, 2H), 7.80 – 7.75 (m, 2H), 7.54 (t, *J* = 8.0 Hz, 1H), 7.41 – 7.32 (m, 2H), 6.89 – 6.83 (m, 2H), 2.68 – 2.62 (m, 2H), 1.68 – 1.61 (m, 2H), 1.40 (dq, *J* = 14.7, 7.4 Hz, 2H), 0.96 (t, *J* = 7.3 Hz, 3H). ^19^F NMR (471 MHz, Chloroform-*d*) δ -115.32 (d, *J* = 11.0 Hz), -117.22 (t, *J* = 9.9 Hz). ^13^C NMR (126 MHz, Chloroform-*d*) δ 160.82 (d, *J* = 7.4 Hz), 160.41, 158.84 (d, *J* = 7.7 Hz), 158.43, 147.42, 146.35 (t, *J* = 9.5 Hz), 142.17, 132.38 (d, *J* = 9.0 Hz), 130.29 (d, *J* = 3.2 Hz), 129.99, 129.96, 126.93, 126.32 (d, *J* = 13.0 Hz), 123.90, 118.58 (d, *J* = 23.9 Hz), 113.75 (t, *J* = 18.1 Hz), 111.95 (d, *J* = 5.3 Hz), 111.79 (d, *J* = 5.4 Hz), 35.46, 33.02, 22.34, 14.01.


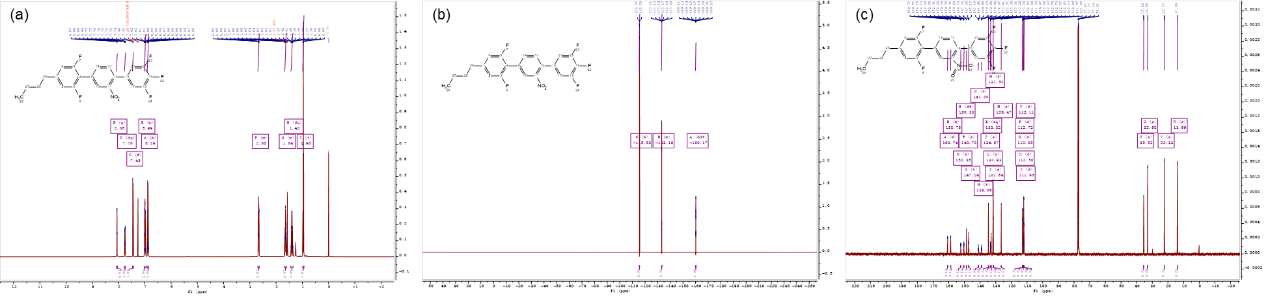


**Scheme S48**. (a) ^1^H, (b) ^19^F and (c) ^13^C NMR spectra of SCUT13b.

**SCUT13b**: ^1^H NMR (500 MHz, Chloroform-*d*) δ 8.06 (q, *J* = 1.3 Hz, 1H), 7.76 (dq, *J* = 7.9, 1.5 Hz, 1H), 7.45 (d, *J* = 8.0 Hz, 1H), 7.03 – 6.96 (m, 2H), 6.91 – 6.86 (m, 2H), 2.69 – 2.64 (m, 2H), 1.68 – 1.60 (m, 2H), 1.40 (dq, *J* = 14.7, 7.4 Hz, 2H), 0.96 (t, *J* = 7.4 Hz, 3H). ^19^F NMR (471 MHz, Chloroform-*d*) δ -115.58 (d, *J* = 8.7 Hz), -132.99 – -133.38 (m), -160.17 (ddt, *J* = 20.0, 13.3, 5.9 Hz). ^13^C NMR (126 MHz, Chloroform-*d*) δ 160.74 (d, *J* = 7.4 Hz), 158.76 (d, *J* = 7.2 Hz), 152.36, 150.33 (dd, *J* = 10.0, 4.0 Hz), 148.70, 147.24 (t, *J* = 9.5 Hz), 141.08 (t, *J* = 15.0 Hz), 139.06 (t, *J* = 15.2 Hz), 134.67, 133.32 (dq, *J* = 9.0, 5.2 Hz), 132.93, 131.64, 131.52, 126.47, 112.85 (d, *J* = 5.8 Hz), 112.72 (d, *J* = 5.4 Hz), 112.50 (d, *J* = 17.9 Hz), 112.11 (d, *J* = 5.1 Hz), 111.95 (d, *J* = 5.0 Hz), 35.52, 32.98, 22.33, 13.99.


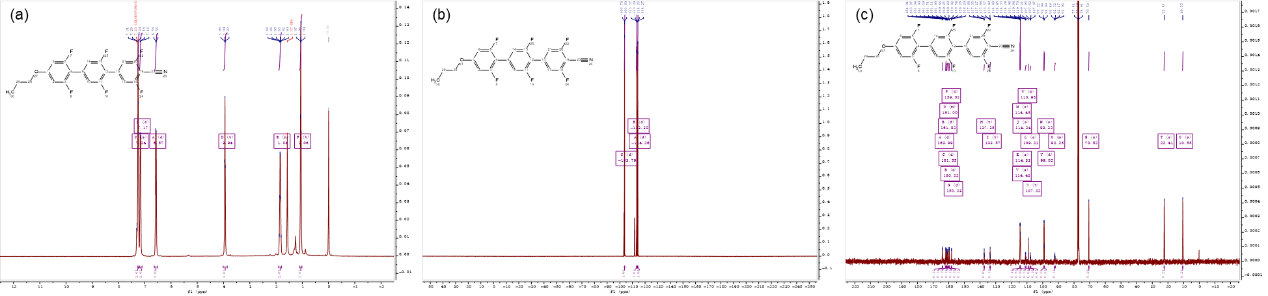


**Scheme S49**. (a) ^1^H, (b) ^19^F and (c) ^13^C NMR spectra of SCUT14a.

**SCUT14a**: ^1^H NMR (500 MHz, Chloroform-*d*) δ 7.24 (s, 2H), 7.17 (d, *J* = 9.7 Hz, 2H), 6.57 (d, *J* = 10.4 Hz, 2H), 3.94 (t, *J* = 6.4 Hz, 2H), 1.84 (h, *J* = 7.1 Hz, 2H), 1.06 (t, *J* = 7.4 Hz, 3H). ^19^F NMR (471 MHz, Chloroform-*d*) δ -103.79 (d, *J* = 9.9 Hz), -113.38 (d, *J* = 11.0 Hz), -114.26 (d, *J* = 10.2 Hz). ^13^C NMR (126 MHz, Chloroform-*d*) δ 163.99 (d, *J* = 4.9 Hz), 161.92 (d, *J* = 4.9 Hz), 161.55 (d, *J* = 9.6 Hz), 161.24 – 160.76 (m), 160.22 (d, *J* = 7.3 Hz), 159.58 (d, *J* = 9.3 Hz), 158.23 (d, *J* = 6.5 Hz), 137.35 (t, *J* = 10.8 Hz), 133.57 (t, *J* = 10.6 Hz), 114.48, 114.45, 114.33, 114.24, 110.95 (d, *J* = 26.9 Hz), 109.21, 107.82 (t, *J* = 15.7 Hz), 99.22, 99.02 (d, *J* = 6.2 Hz), 92.60 – 91.89 (m), 70.52, 22.41, 10.55.


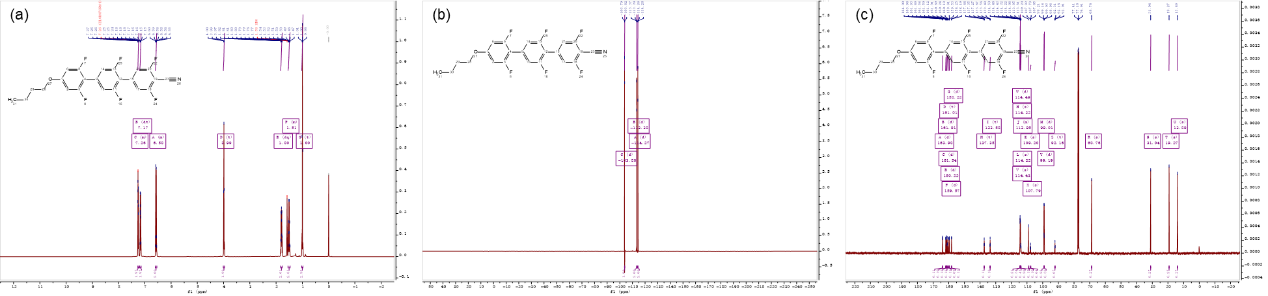


**Scheme S50**. (a) ^1^H, (b) ^19^F and (c) ^13^C NMR spectra of SCUT14b.

**SCUT14b**: ^1^H NMR (500 MHz, Chloroform-*d*) δ 7.27 – 7.23 (m, 2H), 7.17 (dt, *J* = 9.3, 1.3 Hz, 2H), 6.60 – 6.54 (m, 2H), 3.99 (t, *J* = 6.5 Hz, 2H), 1.80 (dq, *J* = 8.7, 6.6 Hz, 2H), 1.55 – 1.46 (m, 2H), 1.00 (t, *J* = 7.4 Hz, 3H). ^19^F NMR (471 MHz, Chloroform-*d*) δ -103.80 (d, *J* = 10.9 Hz), -113.38 (d, *J* = 9.1 Hz), -114.27 (d, *J* = 9.4 Hz). ^13^C NMR (126 MHz, Chloroform-*d*) δ 163.98 (d, *J* = 5.1 Hz), 161.91 (d, *J* = 5.1 Hz), 161.54 (d, *J* = 9.6 Hz), 161.01 (t, *J* = 14.3 Hz), 160.22 (d, *J* = 6.9 Hz), 159.57 (d, *J* = 9.7 Hz), 158.22 (d, *J* = 7.0 Hz), 137.35 (t, *J* = 10.8 Hz), 133.58 (t, *J* = 11.3 Hz), 114.49 (d, *J* = 3.1 Hz), 114.43, 114.32, 114.22, 114.12 – 113.78 (m), 109.20, 107.79, 99.19 (d, *J* = 6.1 Hz), 99.01 (d, *J* = 6.3 Hz), 92.16 (t, *J* = 19.3 Hz), 68.76, 31.04, 19.27, 13.89.


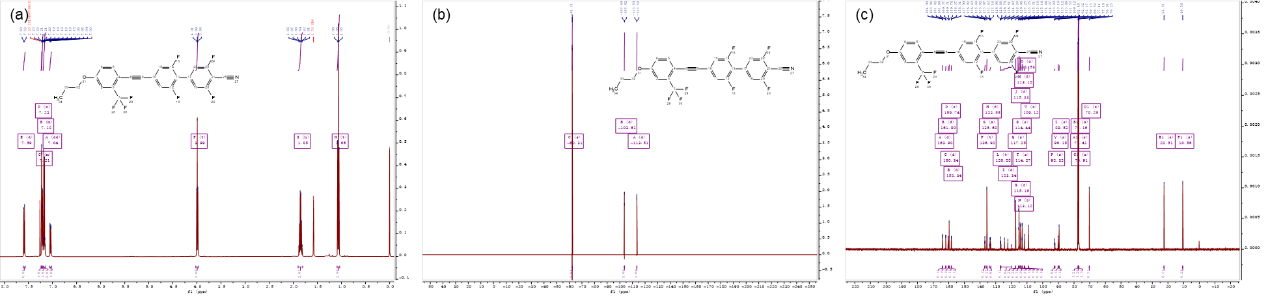


**Scheme S51**. (a) ^1^H, (b) ^19^F and (c) ^13^C NMR spectra of SCUT15a.

**SCUT15a**: ^1^H NMR (500 MHz, Chloroform-*d*) δ 7.59 (d, *J* = 8.5 Hz, 1H), 7.23 (s, 1H), 7.22 – 7.20 (m, 2H), 7.20 – 7.15 (m, 2H), 7.04 (dd, *J* = 8.6, 2.6 Hz, 1H), 3.99 (t, *J* = 6.5 Hz, 2H), 1.85 (h, *J* = 7.4 Hz, 2H), 1.06 (t, *J* = 7.4 Hz, 3H). ^19^F NMR (471 MHz, Chloroform-*d*) δ -62.31, -103.61 (d, *J* = 10.7 Hz), -113.51 (d, *J* = 8.5 Hz). ^13^C NMR (126 MHz, Chloroform-*d*) δ 163.98 (d, *J* = 5.3 Hz), 161.90 (d, *J* = 4.9 Hz), 160.34 (d, *J* = 7.3 Hz), 159.74, 158.34 (d, *J* = 7.3 Hz), 136.92 (t, *J* = 10.6 Hz), 135.68, 133.55 (d, *J* = 30.5 Hz), 126.88 (t, *J* = 12.6 Hz), 123.34 (d, *J* = 273.7 Hz), 117.25, 115.33 (d, *J* = 5.7 Hz), 115.16 (d, *J* = 5.5 Hz), 114.44, 114.27, 113.18 (d, *J* = 5.1 Hz), 113.12, 111.53, 109.12, 92.32, 90.15, 89.52, 77.41, 77.16, 76.91, 70.25, 22.51, 10.56.


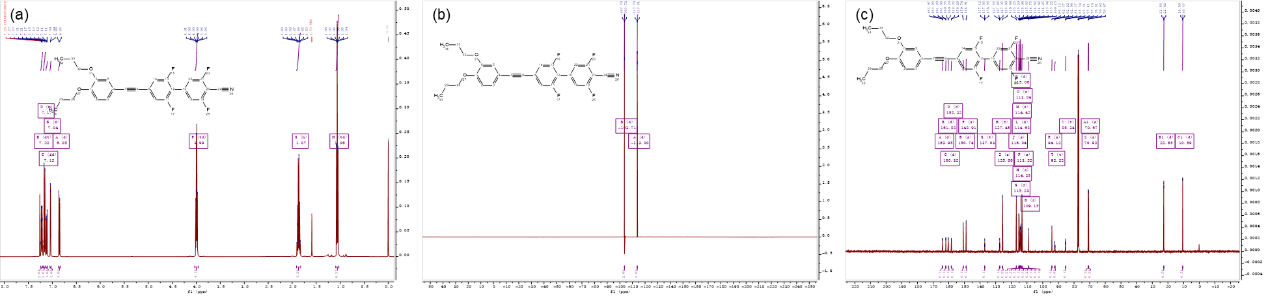


**Scheme S52**. (a) ^1^H, (b) ^19^F and (c) ^13^C NMR spectra of SCUT16a.

**SCUT16a**: ^1^H NMR (500 MHz, Chloroform-*d*) δ 7.22 (dt, *J* = 8.3, 1.5 Hz, 2H), 7.19 – 7.14 (m, 2H), 7.12 (dd, *J* = 8.3, 1.9 Hz, 1H), 7.04 (d, *J* = 1.9 Hz, 1H), 6.85 (d, *J* = 8.3 Hz, 1H), 3.99 (td, *J* = 6.6, 4.1 Hz, 4H), 1.87 (h, *J* = 7.2 Hz, 4H), 1.06 (td, *J* = 7.4, 2.3 Hz, 6H). ^19^F NMR (471 MHz, Chloroform-*d*) δ -103.71 (d, *J* = 10.9 Hz), -113.80 (d, *J* = 8.3 Hz). ^13^C NMR (126 MHz, Chloroform-*d*) δ 163.95 (d, *J* = 5.0 Hz), 161.88 (d, *J* = 5.2 Hz), 160.32 (d, *J* = 7.2 Hz), 158.32 (d, *J* = 7.4 Hz), 150.74, 148.91, 137.03 (t, *J* = 10.6 Hz), 127.45 (t, *J* = 12.5 Hz), 125.66, 116.84, 115.23 (d, *J* = 5.7 Hz), 115.06 (d, *J* = 5.7 Hz), 114.68 (d, *J* = 16.8 Hz), 114.42, 114.25, 113.84, 113.22, 109.15, 94.12, 92.22 (t, *J* = 19.3 Hz), 85.34 (t, *J* = 3.6 Hz), 70.92, 70.67, 22.65 (d, *J* = 6.8 Hz), 10.59 (d, *J* = 3.6 Hz).


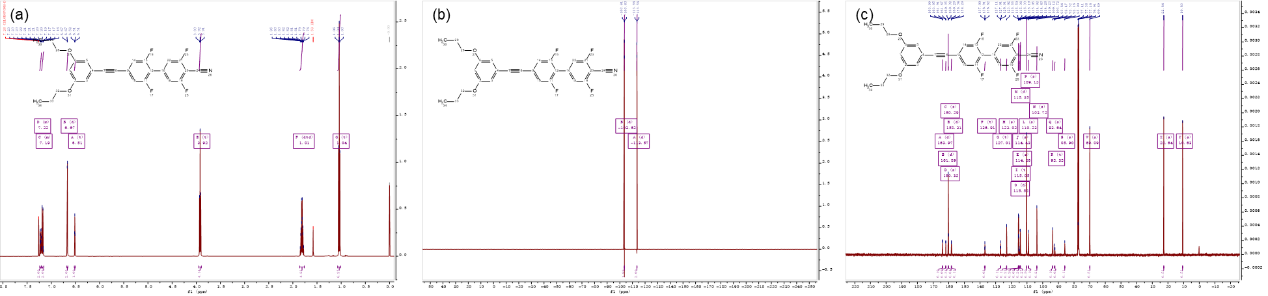


**Scheme S53**. (a) ^1^H, (b) ^19^F and (c) ^13^C NMR spectra of SCUT16b.

**SCUT16b**: ^1^H NMR (500 MHz, Chloroform-*d*) δ 7.24 – 7.20 (m, 2H), 7.20 – 7.15 (m, 2H), 6.67 (d, *J* = 2.3 Hz, 2H), 6.51 (t, *J* = 2.3 Hz, 1H), 3.92 (t, *J* = 6.6 Hz, 4H), 1.81 (dtd, *J* = 13.9, 7.4, 6.5 Hz, 4H), 1.04 (t, *J* = 7.4 Hz, 6H). ^19^F NMR (471 MHz, Chloroform-*d*) δ -103.62 (d, *J* = 10.9 Hz), -113.57 (d, *J* = 8.5 Hz). ^13^C NMR (126 MHz, Chloroform-*d*) δ 163.97 (d, *J* = 4.9 Hz), 161.89 (d, *J* = 5.2 Hz), 160.32, 160.29, 158.31 (d, *J* = 7.2 Hz), 136.91 (t, *J* = 10.8 Hz), 127.01 (t, *J* = 12.5 Hz), 123.02, 115.52 (d, *J* = 5.8 Hz), 115.35 (d, *J* = 5.7 Hz), 115.05 (t, *J* = 17.3 Hz), 114.43, 114.26, 110.23, 109.12, 103.72, 93.64, 92.32 (t, *J* = 19.2 Hz), 85.90, 69.89, 22.64, 10.63.


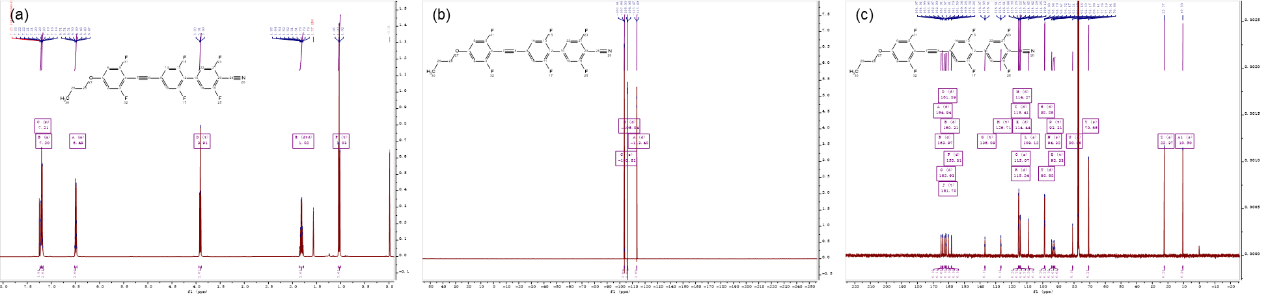


**Scheme S54**. (a) ^1^H, (b) ^19^F and (c) ^13^C NMR spectra of SCUT17a.

**SCUT17a**: ^1^H NMR (500 MHz, Chloroform-*d*) δ 7.23 – 7.20 (m, 2H), 7.20 – 7.17 (m, 2H), 6.52 – 6.47 (m, 2H), 3.91 (t, *J* = 6.5 Hz, 2H), 1.82 (dtd, *J* = 13.9, 7.4, 6.5 Hz, 2H), 1.03 (t, *J* = 7.4 Hz, 3H). ^19^F NMR (471 MHz, Chloroform-*d*) δ -103.61, -106.04 (d, *J* = 10.4 Hz), -113.48 (d, *J* = 8.6 Hz). ^13^C NMR (126 MHz, Chloroform-*d*) δ 164.94 (d, *J* = 8.3 Hz), 163.97 (d, *J* = 5.2 Hz), 162.93 (d, *J* = 8.2 Hz), 161.89 (d, *J* = 5.0 Hz), 161.70 (t, *J* = 13.7 Hz), 160.31 (d, *J* = 7.2 Hz), 158.31 (d, *J* = 7.3 Hz), 136.89 (t, *J* = 10.6 Hz), 126.71 (t, *J* = 12.6 Hz), 115.41 (d, *J* = 5.9 Hz), 115.24 (d, *J* = 5.8 Hz), 115.07, 114.44 (d, *J* = 3.6 Hz), 114.27 (d, *J* = 3.6 Hz), 109.12, 98.86 (d, *J* = 5.1 Hz), 98.68 (d, *J* = 4.5 Hz), 94.32, 93.31 (t, *J* = 20.4 Hz), 92.33 (t, *J* = 19.2 Hz), 80.90, 70.66, 22.37, 10.50.


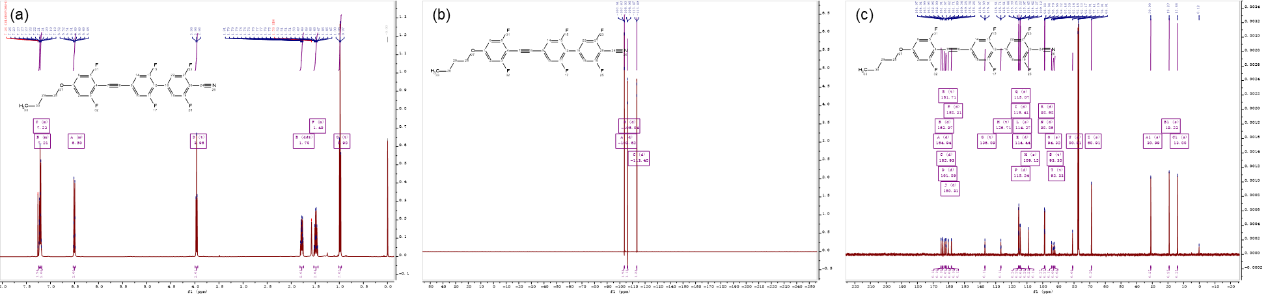


**Scheme S55**. (a) ^1^H, (b) ^19^F and (c) ^13^C NMR spectra of SCUT17b.

**SCUT17b**: ^1^H NMR (500 MHz, Chloroform-*d*) δ 7.24 – 7.21 (m, 2H), 7.21 – 7.18 (m, 2H), 6.53 – 6.48 (m, 2H), 3.96 (t, *J* = 6.5 Hz, 2H), 1.78 (ddt, *J* = 9.0, 7.8, 6.4 Hz, 2H), 1.53 – 1.44 (m, 2H), 0.98 (t, *J* = 7.4 Hz, 3H). ^19^F NMR (471 MHz, Chloroform-*d*) δ -103.62 (d, *J* = 8.8 Hz), -106.04 (d, *J* = 11.3 Hz), -113.48 (d, *J* = 8.5 Hz). ^13^C NMR (126 MHz, Chloroform-*d*) δ 164.94 (d, *J* = 8.3 Hz), 163.97 (d, *J* = 5.0 Hz), 162.93 (d, *J* = 8.3 Hz), 161.89 (d, *J* = 5.0 Hz), 161.71 (t, *J* = 13.8 Hz), 160.31 (d, *J* = 7.3 Hz), 158.31 (d, *J* = 7.1 Hz), 136.89 (t, *J* = 10.6 Hz), 126.71 (t, *J* = 12.5 Hz), 115.41 (d, *J* = 5.9 Hz), 115.24 (d, *J* = 6.0 Hz), 115.07, 114.44 (d, *J* = 3.6 Hz), 114.27, 109.12, 98.86 (d, *J* = 4.9 Hz), 98.68 (d, *J* = 4.9 Hz), 94.32, 93.30 (t, *J* = 20.3 Hz), 92.33 (t, *J* = 19.3 Hz), 80.91, 68.91, 30.99, 19.23, 13.88.


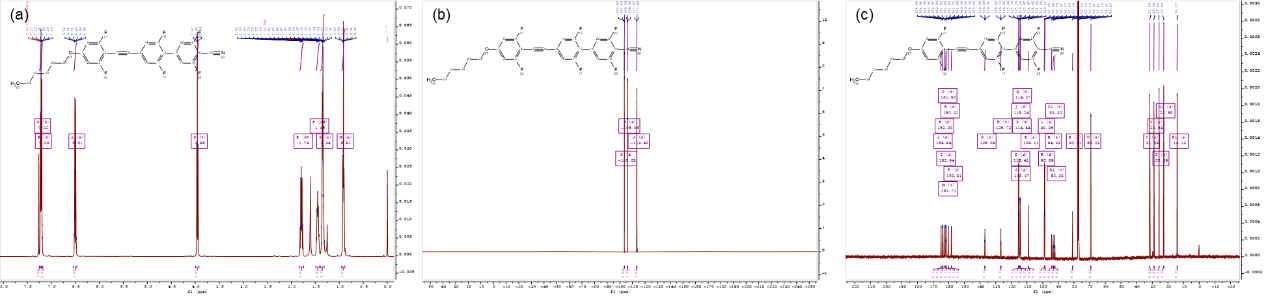


**Scheme S56**. (a) ^1^H, (b) ^19^F and (c) ^13^C NMR spectra of SCUT17c.

**SCUT17c**: ^1^H NMR (500 MHz, Chloroform-*d*) δ 7.22 (d, *J* = 6.4 Hz, 2H), 7.20 (d, *J* = 5.7 Hz, 2H), 6.54 – 6.47 (m, 2H), 3.96 (t, *J* = 6.5 Hz, 2H), 1.79 (dt, *J* = 14.7, 6.7 Hz, 2H), 1.45 (dd, *J* = 10.6, 4.9 Hz, 2H), 1.39 – 1.31 (m, 4H), 0.95 – 0.88 (m, 3H). ^19^F NMR (471 MHz, Chloroform-*d*) δ -103.63 (d, *J* = 8.4 Hz), -106.05 (d, *J* = 9.4 Hz), -113.48 (d, *J* = 8.5 Hz). ^13^C NMR (126 MHz, Chloroform-*d*) δ 164.94 (d, *J* = 8.3 Hz), 163.98 (d, *J* = 5.1 Hz), 162.94 (d, *J* = 8.3 Hz), 161.90 (d, *J* = 5.1 Hz), 161.71 (t, *J* = 13.8 Hz), 160.31 (d, *J* = 7.2 Hz), 158.31 (d, *J* = 7.3 Hz), 136.89 (t, *J* = 10.6 Hz), 126.72 (t, *J* = 12.7 Hz), 115.41 (d, *J* = 6.0 Hz), 115.24 (d, *J* = 5.9 Hz), 115.07, 114.44 (d, *J* = 3.5 Hz), 114.27 (d, *J* = 3.5 Hz), 109.11, 98.86 (d, *J* = 4.8 Hz), 98.69 (d, *J* = 4.7 Hz), 94.33, 93.30 (t, *J* = 20.3 Hz), 92.33 (t, *J* = 19.3 Hz), 80.91, 69.23, 31.59, 28.94, 25.69, 22.69, 14.13.


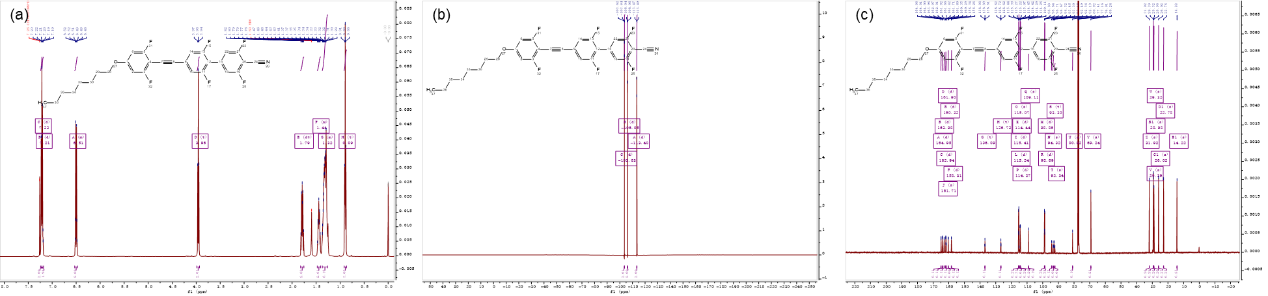


**Scheme S57**. (a) ^1^H, (b) ^19^F and (c) ^13^C NMR spectra of SCUT17d.

**SCUT17d**: ^1^H NMR (500 MHz, Chloroform-*d*) δ 7.22 (d, *J* = 6.4 Hz, 2H), 7.21 (d, *J* = 5.6 Hz, 2H), 6.53 – 6.47 (m, 2H), 3.96 (t, *J* = 6.5 Hz, 2H), 1.79 (dt, *J* = 14.7, 6.7 Hz, 2H), 1.48 – 1.41 (m, 2H), 1.37 – 1.26 (m, 8H), 0.89 (t, *J* = 6.8 Hz, 3H). ^19^F NMR (471 MHz, Chloroform-*d*) δ -103.63 (d, *J* = 8.5 Hz), -106.05 (d, *J* = 8.7 Hz), -113.48 (d, *J* = 8.5 Hz). ^13^C NMR (126 MHz, Chloroform-*d*) δ 164.95 (d, *J* = 8.3 Hz), 163.98 (d, *J* = 4.9 Hz), 162.94 (d, *J* = 8.5 Hz), 161.90 (d, *J* = 5.2 Hz), 161.71, 160.32 (d, *J* = 7.2 Hz), 158.31 (d, *J* = 7.2 Hz), 136.89 (t, *J* = 10.5 Hz), 126.72 (t, *J* = 12.6 Hz), 115.41 (d, *J* = 5.9 Hz), 115.24 (d, *J* = 5.9 Hz), 115.07, 114.44 (d, *J* = 3.6 Hz), 114.27 (d, *J* = 3.2 Hz), 109.11, 98.86 (d, *J* = 4.8 Hz), 98.69 (d, *J* = 4.8 Hz), 94.32, 93.30 (t, *J* = 20.3 Hz), 92.34, 80.92, 69.24, 31.92, 29.39, 29.32, 28.98, 26.02, 22.78, 14.22.


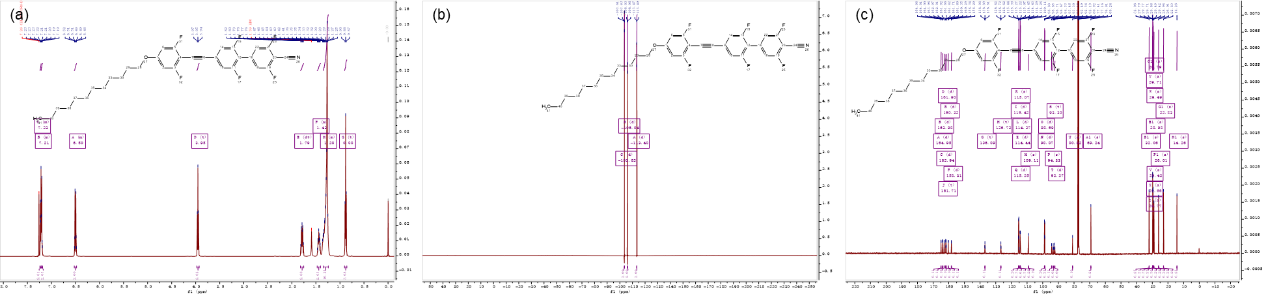


**Scheme S58**. (a) ^1^H, (b) ^19^F and (c) ^13^C NMR spectra of SCUT17e.

**SCUT17e**: ^1^H NMR (500 MHz, Chloroform-*d*) δ 7.24 – 7.21 (m, 2H), 7.21 – 7.18 (m, 2H), 6.52 – 6.48 (m, 2H), 3.95 (t, *J* = 6.5 Hz, 2H), 1.79 (dt, *J* = 14.6, 6.7 Hz, 2H), 1.47 – 1.40 (m, 2H), 1.35 – 1.24 (m, 16H), 0.88 (t, *J* = 6.9 Hz, 3H). ^19^F NMR (471 MHz, Chloroform-*d*) δ -103.62 (d, *J* = 8.4 Hz), -106.04 (d, *J* = 10.7 Hz), -113.48 (d, *J* = 8.5 Hz). ^13^C NMR (126 MHz, Chloroform-*d*) δ 164.95 (d, *J* = 8.3 Hz), 163.98 (d, *J* = 5.4 Hz), 162.94 (d, *J* = 8.4 Hz), 161.90 (d, *J* = 5.0 Hz), 161.71 (t, *J* = 13.8 Hz), 160.32 (d, *J* = 7.3 Hz), 158.31 (d, *J* = 7.2 Hz), 136.89 (t, *J* = 10.7 Hz), 126.72 (t, *J* = 12.5 Hz), 115.42 (d, *J* = 6.0 Hz), 115.25 (d, *J* = 6.0 Hz), 115.07, 114.44 (d, *J* = 3.4 Hz), 114.27 (d, *J* = 3.3 Hz), 109.11, 98.87 (d, *J* = 4.8 Hz), 98.69 (d, *J* = 4.6 Hz), 94.33, 93.30 (t, *J* = 20.3 Hz), 92.27 (d, *J* = 19.2 Hz), 80.92, 69.24, 32.06, 29.79, 29.77, 29.71, 29.66, 29.49, 29.42, 28.98, 26.01, 22.83, 14.26.


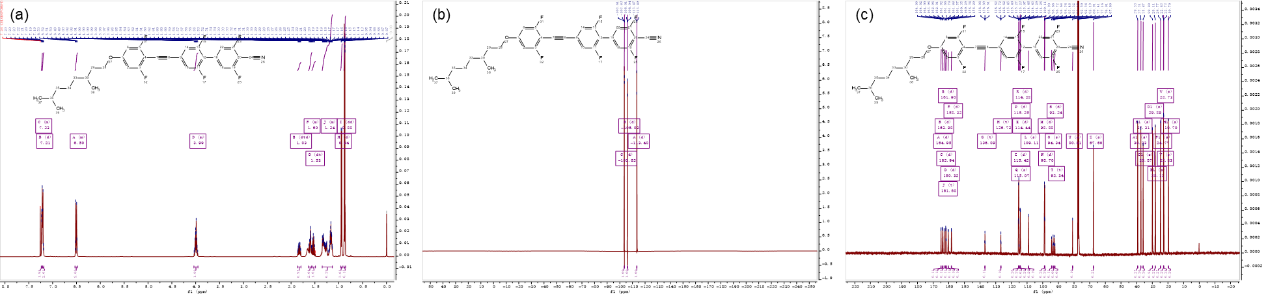


**Scheme S59**. (a) ^1^H, (b) ^19^F and (c) ^13^C NMR spectra of SCUT17f.

**SCUT17f**: ^1^H NMR (500 MHz, Chloroform-*d*) δ 7.24 – 7.22 (m, 2H), 7.21 (d, *J* = 5.0 Hz, 2H), 6.53 – 6.48 (m, 2H), 4.04 – 3.95 (m, 2H), 1.83 (dtd, *J* = 12.3, 7.0, 4.9 Hz, 1H), 1.64 – 1.57 (m, 2H), 1.53 (dt, *J* = 13.3, 6.6 Hz, 1H), 1.35 – 1.13 (m, 6H), 0.94 (d, *J* = 6.5 Hz, 3H), 0.88 (dd, *J* = 6.6, 0.7 Hz, 6H). ^19^F NMR (471 MHz, Chloroform-*d*) δ -103.62 (d, *J* = 10.2 Hz), -106.03 (d, *J* = 10.7 Hz), -113.48 (d, *J* = 8.4 Hz). ^13^C NMR (126 MHz, Chloroform-*d*) δ 164.95 (d, *J* = 8.5 Hz), 163.98 (d, *J* = 5.2 Hz), 162.94 (d, *J* = 8.3 Hz), 161.90 (d, *J* = 4.9 Hz), 161.68 (t, *J* = 13.9 Hz), 160.32 (d, *J* = 7.2 Hz), 158.32 (d, *J* = 7.2 Hz), 136.89 (t, *J* = 10.7 Hz), 126.72 (t, *J* = 12.6 Hz), 115.42 (d, *J* = 5.8 Hz), 115.25 (d, *J* = 5.8 Hz), 115.07, 114.44 (d, *J* = 3.3 Hz), 114.28 (d, *J* = 3.6 Hz), 109.11, 98.88 (d, *J* = 4.8 Hz), 98.70 (d, *J* = 4.7 Hz), 94.34, 93.24 (d, *J* = 20.3 Hz), 92.34 (t, *J* = 19.2 Hz), 80.91, 67.60, 39.33, 37.31, 35.87, 29.89, 28.11, 24.77, 22.83, 22.73, 19.70.


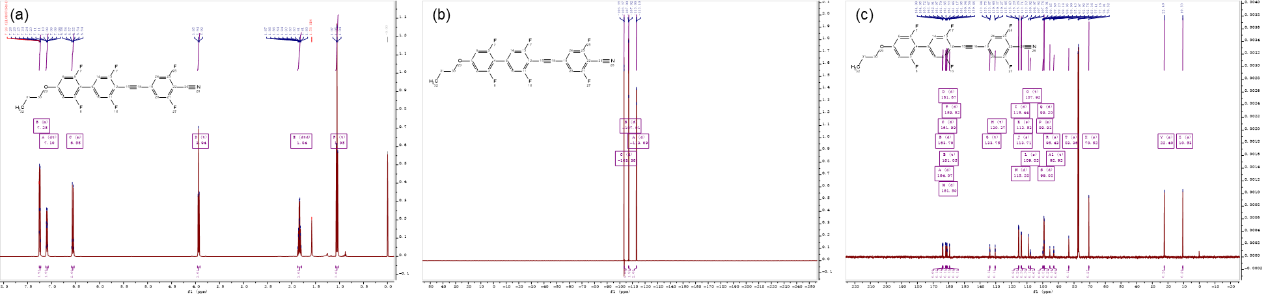


**Scheme S60**. (a) ^1^H, (b) ^19^F and (c) ^13^C NMR spectra of SCUT18a.

**SCUT18a**: ^1^H NMR (500 MHz, Chloroform-*d*) δ 7.26 – 7.22 (m, 2H), 7.10 (dt, *J* = 8.3, 1.3 Hz, 2H), 6.58 – 6.53 (m, 2H), 3.94 (t, *J* = 6.5 Hz, 2H), 1.84 (dtd, *J* = 13.9, 7.4, 6.5 Hz, 2H), 1.05 (t, *J* = 7.4 Hz, 3H). ^19^F NMR (471 MHz, Chloroform-*d*) δ -103.36 (d, *J* = 8.1 Hz), -107.01 (d, *J* = 9.8 Hz), -113.09 (d, *J* = 9.2 Hz). ^13^C NMR (126 MHz, Chloroform-*d*) δ 164.07 (d, *J* = 5.5 Hz), 163.70 (d, *J* = 5.8 Hz), 161.99 (d, *J* = 5.4 Hz), 161.67 (d, *J* = 6.0 Hz), 161.50 (d, *J* = 9.5 Hz), 161.05 (t, *J* = 14.3 Hz), 159.52 (d, *J* = 9.6 Hz), 133.75 (t, *J* = 10.7 Hz), 130.37 (t, *J* = 11.8 Hz), 115.44 (d, *J* = 3.8 Hz), 115.28 (d, *J* = 3.7 Hz), 113.71, 113.53, 109.02, 107.92 (t, *J* = 18.2 Hz), 100.05 – 99.64 (m), 99.20 (d, *J* = 6.2 Hz), 99.02 (d, *J* = 6.1 Hz), 95.43, 92.92 (t, *J* = 19.4 Hz), 83.36, 70.52, 22.40, 10.53.


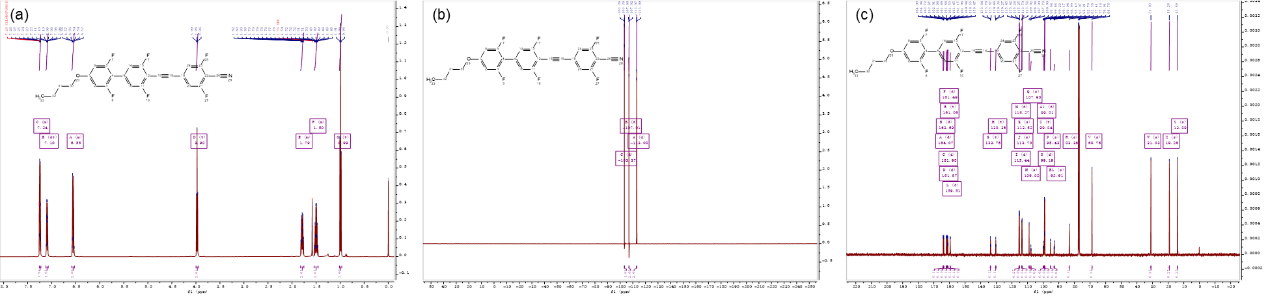


**Scheme S61**. (a) ^1^H, (b) ^19^F and (c) ^13^C NMR spectra of SCUT18b.

**SCUT18b**: ^1^H NMR (500 MHz, Chloroform-*d*) δ 7.26 – 7.22 (m, 2H), 7.10 (dt, *J* = 8.3, 1.3 Hz, 2H), 6.58 – 6.53 (m, 2H), 3.98 (t, *J* = 6.5 Hz, 2H), 1.82 – 1.75 (m, 2H), 1.54 – 1.46 (m, 2H), 0.99 (t, *J* = 7.4 Hz, 3H). ^19^F NMR (471 MHz, Chloroform-*d*) δ -103.37 (d, *J* = 8.9 Hz), -107.01 (d, *J* = 9.4 Hz), -113.08 (d, *J* = 11.0 Hz). ^13^C NMR (126 MHz, Chloroform-*d*) δ 164.07 (d, *J* = 5.4 Hz), 163.69 (d, *J* = 5.9 Hz), 161.98 (d, *J* = 5.2 Hz), 161.67 (d, *J* = 6.0 Hz), 161.49 (d, *J* = 9.5 Hz), 161.06 (t, *J* = 14.4 Hz), 159.51 (d, *J* = 9.7 Hz), 133.76 (t, *J* = 10.8 Hz), 130.36 (t, *J* = 12.0 Hz), 115.44 (d, *J* = 3.8 Hz), 115.27 (d, *J* = 3.7 Hz), 113.70, 113.52, 109.02, 107.90, 99.84 (t, *J* = 19.6 Hz), 99.19 (d, *J* = 6.0 Hz), 99.01 (d, *J* = 6.1 Hz), 95.43, 92.91, 83.36, 68.76, 31.03, 19.26, 13.89.


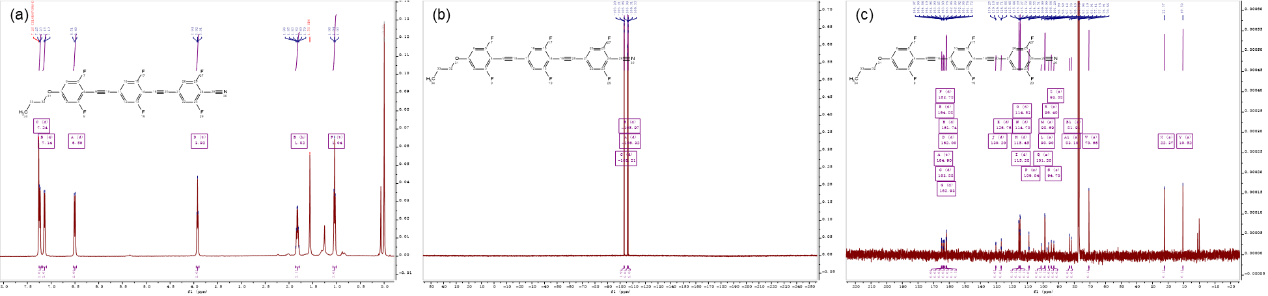


**Scheme S62**. (a) ^1^H, (b) ^19^F and (c) ^13^C NMR spectra of SCUT19a.

**SCUT19a**: ^1^H NMR (500 MHz, Chloroform-*d*) δ 7.24 (d, *J* = 8.2 Hz, 2H), 7.14 (d, *J* = 7.6 Hz, 2H), 6.50 (d, *J* = 9.4 Hz, 2H), 3.92 (t, *J* = 6.5 Hz, 2H), 1.82 (h, *J* = 7.2 Hz, 2H), 1.04 (t, *J* = 7.4 Hz, 3H). ^19^F NMR (471 MHz, Chloroform-*d*) δ -103.21 (d, *J* = 8.4 Hz), -105.97 (d, *J* = 10.7 Hz), -106.32 (d, *J* = 8.4 Hz). ^19^F NMR (126 MHz, Chloroform-*d*) δ 164.90 (t, *J* = 8.2 Hz), 164.08 (d, *J* = 4.8 Hz), 163.88 (d, *J* = 6.2 Hz), 163.78 (d, *J* = 5.3 Hz), 162.93 (d, *J* = 8.4 Hz), 162.00 (d, *J* = 5.5 Hz), 161.74 (d, *J* = 4.5 Hz), 130.20 (d, *J* = 11.9 Hz), 126.76 (d, *J* = 12.5 Hz), 115.45 (d, *J* = 3.2 Hz), 115.28 (d, *J* = 3.5 Hz), 114.70 (d, *J* = 4.7 Hz), 114.53 (d, *J* = 4.8 Hz), 109.16 – 108.76 (m), 101.20, 98.90, 98.69, 96.40, 94.70, 93.08, 83.10, 81.91, 70.66, 22.37, 10.52.


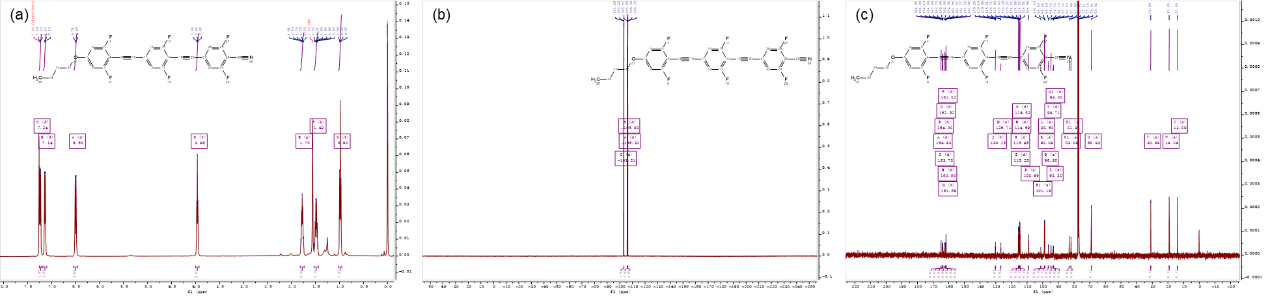


**Scheme S63**. (a) ^1^H, (b) ^19^F and (c) ^13^C NMR spectra of SCUT19b.

**SCUT19b**: ^1^H NMR (500 MHz, Chloroform-*d*) δ 7.24 (d, *J* = 8.3 Hz, 2H), 7.14 (d, *J* = 7.4 Hz, 2H), 6.50 (d, *J* = 9.5 Hz, 2H), 3.96 (t, *J* = 6.5 Hz, 2H), 1.78 (p, *J* = 6.8 Hz, 2H), 1.49 (h, *J* = 7.4 Hz, 2H), 0.98 (t, *J* = 7.4 Hz, 3H). ^19^F NMR (471 MHz, Chloroform-*d*) δ -103.21 (d, *J* = 8.5 Hz), -105.98 (d, *J* = 8.8 Hz), -106.32 (d, *J* = 9.1 Hz). ^13^C NMR (126 MHz, Chloroform-*d*) δ 164.93 (d, *J* = 8.5 Hz), 164.08 (d, *J* = 5.1 Hz), 163.78 (d, *J* = 6.0 Hz), 162.92 (d, *J* = 8.3 Hz), 162.00 (d, *J* = 5.3 Hz), 161.82 (d, *J* = 13.3 Hz), 161.69 (d, *J* = 8.6 Hz), 130.15 (t, *J* = 11.8 Hz), 126.71, 115.45 (d, *J* = 3.5 Hz), 115.28 (d, *J* = 3.3 Hz), 114.69 (d, *J* = 4.8 Hz), 114.53 (d, *J* = 4.7 Hz), 108.99, 101.19, 98.89, 98.68, 96.38, 94.71 (d, *J* = 3.0 Hz), 93.32, 93.08, 83.09, 81.91, 68.92, 30.99, 19.24, 13.89.


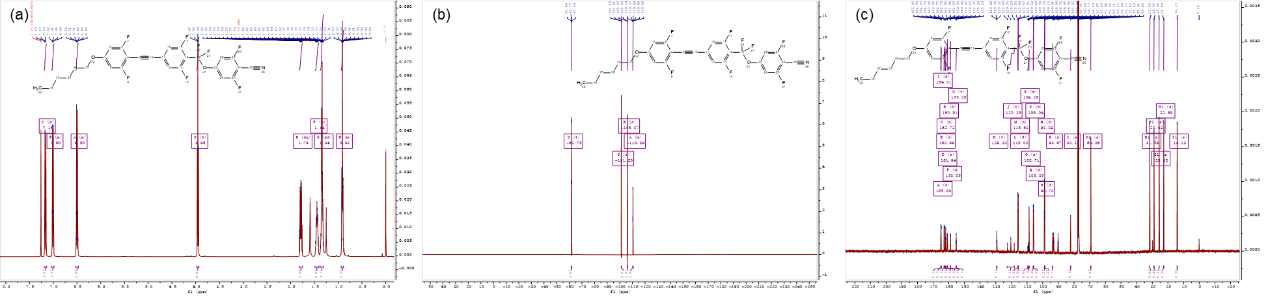


**Scheme S64**. (a) ^1^H, (b) ^19^F and (c) ^13^C NMR spectra of SCUT20a.

**SCUT20a**: ^1^H NMR (500 MHz, Chloroform-*d*) δ 7.16 (d, *J* = 9.6 Hz, 2H), 7.03 – 6.98 (m, 2H), 6.53 – 6.47 (m, 2H), 3.96 (t, *J* = 6.5 Hz, 2H), 1.79 (dq, *J* = 8.3, 6.6 Hz, 2H), 1.49 – 1.41 (m, 2H), 1.34 (pd, *J* = 6.1, 5.5, 3.4 Hz, 4H), 0.94 – 0.88 (m, 3H). ^19^F NMR (471 MHz, Chloroform-*d*) δ -61.75 (t, *J* = 26.7 Hz), -101.20 (d, *J* = 10.9 Hz), -105.87 (d, *J* = 10.6 Hz), -110.22 – -110.69 (m). ^13^C NMR (126 MHz, Chloroform-*d*) δ 165.00 (d, *J* = 8.4 Hz), 164.81 (d, *J* = 6.6 Hz), 162.99 (d, *J* = 8.4 Hz), 162.73 (d, *J* = 6.6 Hz), 161.94 (t, *J* = 13.8 Hz), 160.91 (d, *J* = 5.5 Hz), 158.85 (d, *J* = 4.9 Hz), 155.26 (t, *J* = 13.7 Hz), 129.32 (t, *J* = 12.8 Hz), 120.35 (t, *J* = 269.8 Hz), 115.82 (d, *J* = 3.9 Hz), 115.62 (d, *J* = 3.4 Hz), 109.61 – 108.85 (m), 108.71, 106.04 (d, *J* = 3.8 Hz), 105.85 (d, *J* = 3.9 Hz), 98.89 (d, *J* = 4.7 Hz), 98.72 (d, *J* = 4.6 Hz), 93.67, 82.15, 69.26, 31.59, 28.93, 25.68, 22.69, 14.13.


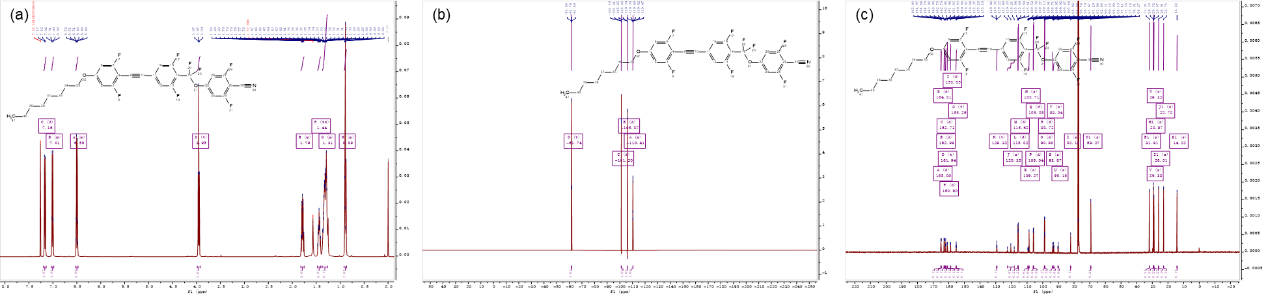


**Scheme S65**. (a) ^1^H, (b) ^19^F and (c) ^13^C NMR spectra of SCUT20b.

**SCUT20b**: ^1^H NMR (500 MHz, Chloroform-*d*) δ 7.16 (d, *J* = 9.6 Hz, 2H), 7.03 – 6.98 (m, 2H), 6.53 – 6.47 (m, 2H), 3.95 (t, *J* = 6.5 Hz, 2H), 1.82 – 1.75 (m, 2H), 1.44 (td, *J* = 9.9, 8.6, 4.6 Hz, 2H), 1.37 – 1.26 (m, 8H), 0.92 – 0.87 (m, 3H). ^19^F NMR (471 MHz, Chloroform-*d*) δ -61.74 (t, *J* = 26.4 Hz), -101.20 (d, *J* = 9.4 Hz), -105.87 (d, *J* = 11.3 Hz), -110.31 – -110.55 (m). ^13^C NMR (126 MHz, Chloroform-*d*) δ 165.00 (d, *J* = 8.3 Hz), 164.81 (d, *J* = 6.5 Hz), 162.99 (d, *J* = 8.2 Hz), 162.73 (d, *J* = 6.6 Hz), 161.94 (t, *J* = 13.7 Hz), 160.90 (d, *J* = 5.9 Hz), 158.85 (d, *J* = 4.5 Hz), 155.26 (t, *J* = 13.4 Hz), 129.32 (t, *J* = 12.8 Hz), 120.35, 115.82 (d, *J* = 4.6 Hz), 115.62 (d, *J* = 3.4 Hz), 109.27, 108.71, 106.04 (d, *J* = 3.8 Hz), 105.85 (d, *J* = 3.4 Hz), 98.90 (d, *J* = 4.6 Hz), 98.72 (d, *J* = 4.8 Hz), 93.67, 93.04, 90.16, 82.16, 69.27, 31.91, 29.38, 29.32, 28.97, 26.01, 22.78, 14.22.


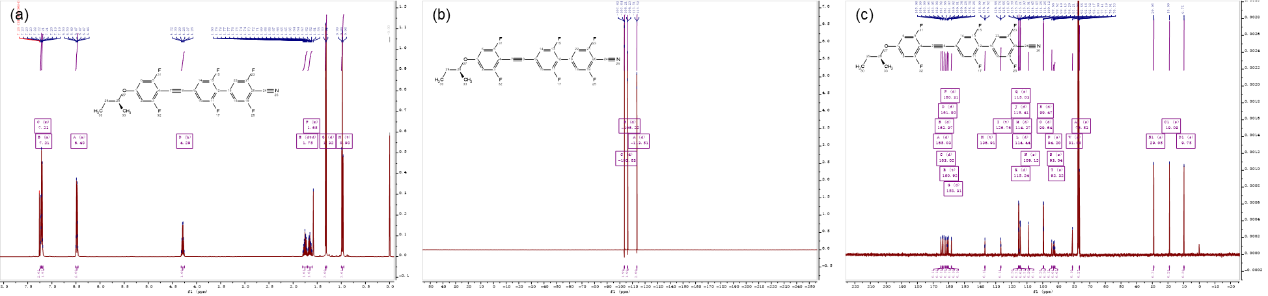


**Scheme S66**. (a) ^1^H, (b) ^19^F and (c) ^13^C NMR spectra of SCUT21a.

**SCUT21a**: ^1^H NMR (500 MHz, Chloroform-*d*) δ 7.24 – 7.21 (m, 2H), 7.21 – 7.18 (m, 2H), 6.51 – 6.46 (m, 2H), 4.29 (h, *J* = 6.1 Hz, 1H), 1.75 (dtd, *J* = 14.9, 7.5, 6.2 Hz, 1H), 1.70 – 1.60 (m, 1H), 1.32 (d, *J* = 6.1 Hz, 3H), 0.98 (t, *J* = 7.5 Hz, 3H). ^19^F NMR (471 MHz, Chloroform-*d*) δ -103.63 (d, *J* = 8.7 Hz), -106.22 (d, *J* = 8.5 Hz), -113.51 (d, *J* = 8.4 Hz). ^13^C NMR (126 MHz, Chloroform-*d*) δ 165.03 (d, *J* = 8.5 Hz), 163.97 (d, *J* = 5.0 Hz), 163.02 (d, *J* = 8.5 Hz), 161.90 (d, *J* = 5.2 Hz), 160.92 (t, *J* = 13.8 Hz), 160.31 (d, *J* = 7.2 Hz), 158.31 (d, *J* = 7.2 Hz), 136.91 (t, *J* = 10.8 Hz), 126.76 (t, *J* = 12.6 Hz), 115.41 (d, *J* = 5.9 Hz), 115.24 (d, *J* = 5.9 Hz), 115.03, 114.44 (d, *J* = 3.3 Hz), 114.27 (d, *J* = 3.2 Hz), 109.12, 99.64 (d, *J* = 4.8 Hz), 99.47 (d, *J* = 4.8 Hz), 94.20, 93.04, 92.32, 81.00, 76.53, 29.05, 19.09, 9.75.


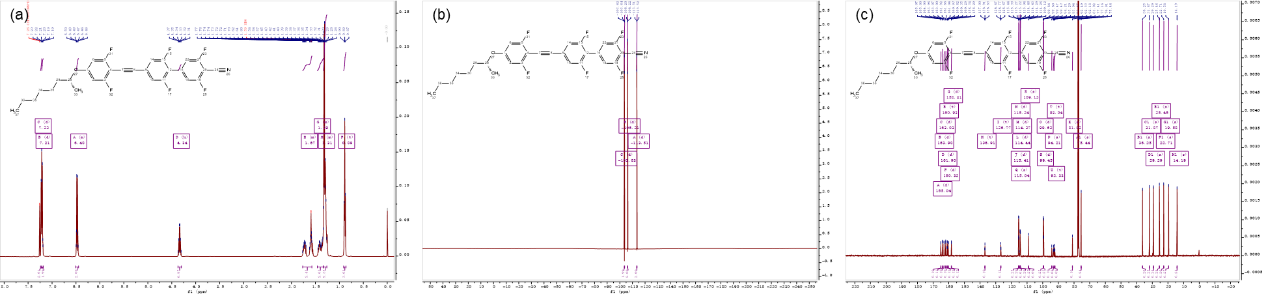


**Scheme S67**. (a) ^1^H, (b) ^19^F and (c) ^13^C NMR spectra of SCUT21b.

**SCUT21b**: ^1^H NMR (500 MHz, Chloroform-*d*) δ 7.22 (d, *J* = 5.8 Hz, 2H), 7.21 (d, *J* = 5.1 Hz, 2H), 6.51 – 6.45 (m, 2H), 4.34 (h, *J* = 6.1 Hz, 1H), 1.77 – 1.57 (m, 2H), 1.46 – 1.33 (m, 2H), 1.33 – 1.26 (m, 9H), 0.89 (t, *J* = 6.8 Hz, 3H). ^19^F NMR (471 MHz, Chloroform-*d*) δ -103.63 (d, *J* = 10.9 Hz), -106.21 (d, *J* = 8.6 Hz), -113.51 (d, *J* = 8.4 Hz). ^13^C NMR (126 MHz, Chloroform-*d*) δ 165.04 (d, *J* = 8.6 Hz), 163.98 (d, *J* = 5.3 Hz), 163.03 (d, *J* = 8.4 Hz), 161.90 (d, *J* = 5.0 Hz), 160.93 (t, *J* = 13.7 Hz), 160.32 (d, *J* = 7.2 Hz), 158.31 (d, *J* = 7.3 Hz), 136.91 (t, *J* = 10.6 Hz), 126.77 (t, *J* = 12.7 Hz), 115.41 (d, *J* = 5.8 Hz), 115.24 (d, *J* = 6.0 Hz), 115.04, 114.44 (d, *J* = 3.3 Hz), 114.27 (d, *J* = 3.4 Hz), 109.12, 99.62 (d, *J* = 4.8 Hz), 99.45 (d, *J* = 4.7 Hz), 94.21, 93.04 (t, *J* = 20.3 Hz), 92.33 (t, *J* = 19.2 Hz), 81.02, 75.44, 36.25, 31.87, 29.29, 25.46, 22.71, 19.58, 14.19.


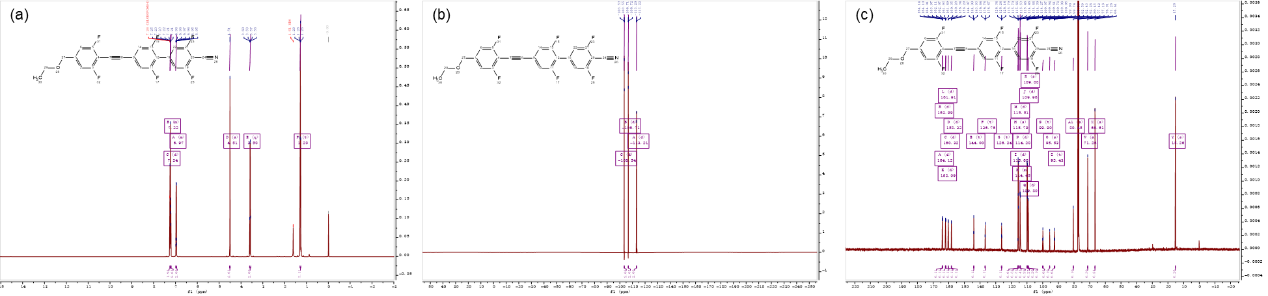


**Scheme S68**. (a) ^1^H, (b) ^19^F and (c) ^13^C NMR spectra of SCUT22a.

**SCUT22a**: ^1^H NMR (500 MHz, Chloroform-*d*) δ 7.24 (d, *J* = 8.4 Hz, 2H), 7.23 – 7.20 (m, 2H), 6.99 – 6.94 (m, 2H), 4.51 (s, 2H), 3.58 (q, *J* = 7.0 Hz, 2H), 1.28 (t, *J* = 7.0 Hz, 3H). ^19^F NMR (471 MHz, Chloroform-*d*) δ -103.54 (d, *J* = 8.9 Hz), -106.71 (d, *J* = 8.6 Hz), -113.21 (d, *J* = 8.9 Hz). ^13^C NMR (126 MHz, Chloroform-*d*) δ 164.12 (d, *J* = 5.4 Hz), 163.99 (d, *J* = 5.2 Hz), 162.09 (d, *J* = 5.4 Hz), 161.91 (d, *J* = 5.2 Hz), 160.32 (d, *J* = 7.2 Hz), 158.32 (d, *J* = 7.2 Hz), 144.00 (t, *J* = 8.9 Hz), 136.76 (t, *J* = 10.7 Hz), 126.24 (t, *J* = 12.6 Hz), 115.79, 115.68 (d, *J* = 6.0 Hz), 115.51 (d, *J* = 5.8 Hz), 114.62 – 114.38 (m), 114.28 (d, *J* = 3.3 Hz), 109.96 (d, *J* = 4.4 Hz), 109.80 (d, *J* = 4.3 Hz), 109.08, 99.90 (t, *J* = 19.8 Hz), 95.53, 92.43 (t, *J* = 19.2 Hz), 80.45, 71.26, 66.61, 15.26.


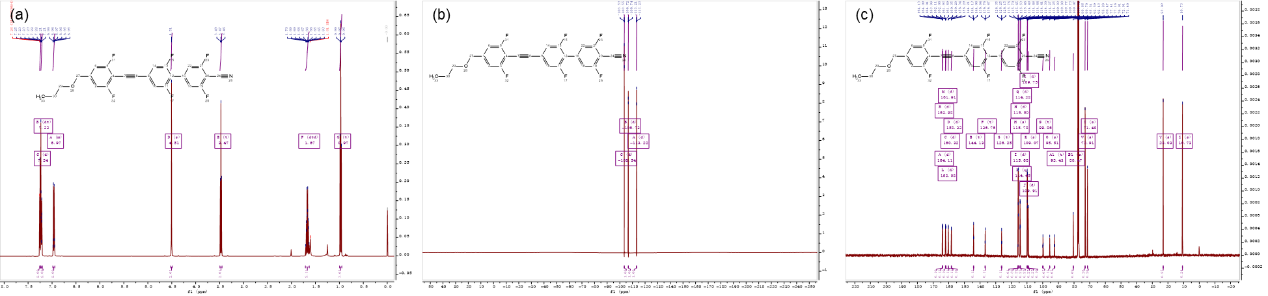


**Scheme S69**. (a) ^1^H, (b) ^19^F and (c) ^13^C NMR spectra of SCUT22b.

**SCUT22b**: ^1^H NMR (500 MHz, Chloroform-*d*) δ 7.24 (d, *J* = 8.4 Hz, 2H), 7.22 (dt, *J* = 7.2, 1.3 Hz, 2H), 6.98 – 6.94 (m, 2H), 4.51 (s, 2H), 3.47 (t, *J* = 6.6 Hz, 2H), 1.67 (dtd, *J* = 14.0, 7.4, 6.6 Hz, 2H), 0.97 (t, *J* = 7.4 Hz, 3H). ^19^F NMR (471 MHz, Chloroform-*d*) δ -103.54 (d, *J* = 8.5 Hz), -106.73 (d, *J* = 8.9 Hz), -113.22 (d, *J* = 8.4 Hz). ^13^C NMR (126 MHz, Chloroform-*d*) δ 164.11 (d, *J* = 5.5 Hz), 163.98 (d, *J* = 5.2 Hz), 162.08 (d, *J* = 5.4 Hz), 161.91 (d, *J* = 5.1 Hz), 160.32 (d, *J* = 7.2 Hz), 158.32 (d, *J* = 7.0 Hz), 144.13 (t, *J* = 8.9 Hz), 136.76 (t, *J* = 10.6 Hz), 126.25 (t, *J* = 12.6 Hz), 115.78, 115.68 (d, *J* = 5.9 Hz), 115.50 (d, *J* = 5.8 Hz), 114.45 (q, *J* = 3.0 Hz), 114.28 (d, *J* = 3.1 Hz), 109.91 (d, *J* = 4.4 Hz), 109.75 (d, *J* = 4.6 Hz), 109.07, 99.86 (t, *J* = 19.8 Hz), 95.51, 92.43 (t, *J* = 19.3 Hz), 80.47, 72.91, 71.40, 23.03, 10.73.


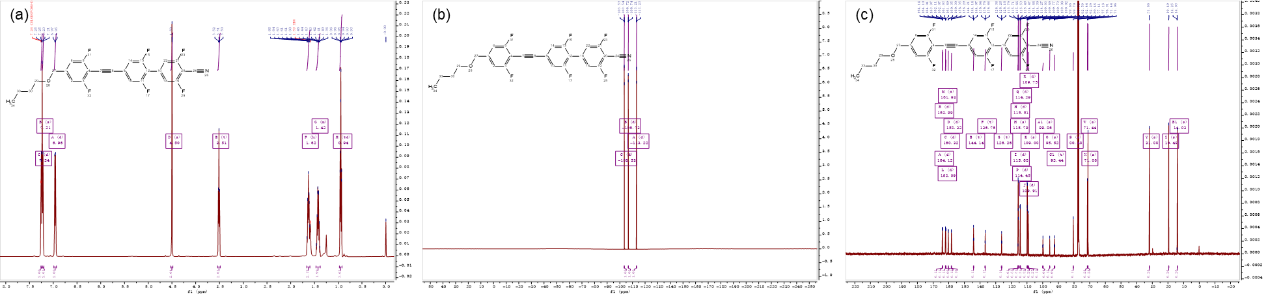


**Scheme S70**. (a) ^1^H, (b) ^19^F and (c) ^13^C NMR spectra of SCUT22c.

**SCUT22c**: ^1^H NMR (500 MHz, Chloroform-*d*) δ 7.24 (d, *J* = 8.1 Hz, 2H), 7.21 (s, 2H), 6.96 (d, *J* = 8.4 Hz, 2H), 4.50 (s, 2H), 3.51 (t, *J* = 6.3 Hz, 2H), 1.62 (h, *J* = 6.3 Hz, 2H), 1.47 – 1.38 (m, 2H), 0.94 (td, *J* = 7.4, 1.9 Hz, 3H). ^19^F NMR (471 MHz, Chloroform-*d*) δ -103.53 (d, *J* = 9.1 Hz), -106.73 (d, *J* = 8.5 Hz), -113.22 (d, *J* = 8.5 Hz). ^13^C NMR (126 MHz, Chloroform-*d*) δ 164.12 (d, *J* = 5.4 Hz), 163.99 (d, *J* = 5.0 Hz), 162.09 (d, *J* = 5.5 Hz), 161.93, 160.32 (d, *J* = 7.1 Hz), 158.32 (d, *J* = 7.2 Hz), 144.14 (t, *J* = 9.0 Hz), 136.76 (t, *J* = 10.6 Hz), 126.26 (t, *J* = 12.6 Hz), 115.79, 115.68 (d, *J* = 6.1 Hz), 115.51 (d, *J* = 5.7 Hz), 114.45 (d, *J* = 3.4 Hz), 114.29 (d, *J* = 3.2 Hz), 109.91 (d, *J* = 4.3 Hz), 109.75 (d, *J* = 4.2 Hz), 109.08, 99.86, 95.52, 92.44 (t, *J* = 19.2 Hz), 80.48, 71.44, 71.06, 31.88, 19.49, 14.03.


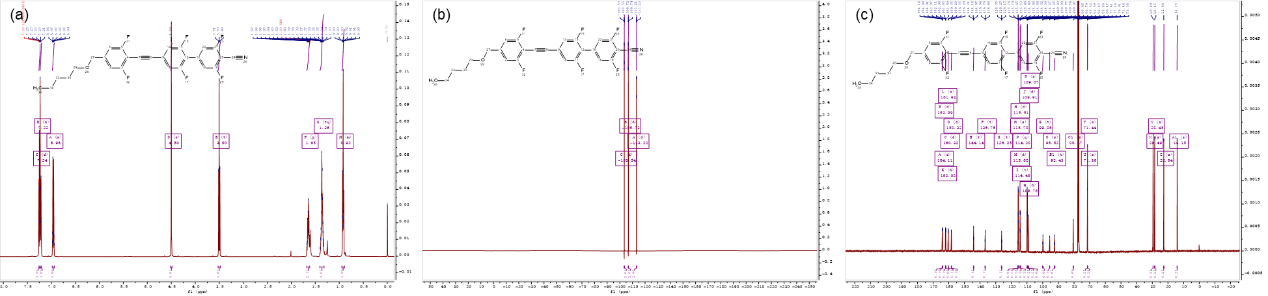


**Scheme S71**. (a) ^1^H, (b) ^19^F and (c) ^13^C NMR spectra of SCUT22d.

**SCUT22d**: ^1^H NMR (500 MHz, Chloroform-*d*) δ 7.24 (d, *J* = 8.4 Hz, 2H), 7.22 (d, *J* = 7.5 Hz, 2H), 6.98 – 6.94 (m, 2H), 4.50 (s, 2H), 3.50 (t, *J* = 6.6 Hz, 2H), 1.65 (p, *J* = 6.9 Hz, 2H), 1.36 (tq, *J* = 6.3, 2.9 Hz, 4H), 0.95 – 0.89 (m, 3H). ^19^F NMR (471 MHz, Chloroform-*d*) δ -103.54 (d, *J* = 8.2 Hz), -106.73 (d, *J* = 8.4 Hz), -113.22 (d, *J* = 8.2 Hz). ^13^C NMR (126 MHz, Chloroform-*d*) δ 164.11 (d, *J* = 5.5 Hz), 163.99 (d, *J* = 5.1 Hz), 162.08 (d, *J* = 5.5 Hz), 161.93, 160.32 (d, *J* = 7.1 Hz), 158.32 (d, *J* = 7.2 Hz), 144.14 (t, *J* = 8.9 Hz), 136.76 (t, *J* = 10.6 Hz), 126.25 (t, *J* = 12.6 Hz), 115.78, 115.68 (d, *J* = 6.1 Hz), 115.51 (d, *J* = 5.8 Hz), 114.46 (t, *J* = 3.1 Hz), 114.28 (q, *J* = 3.1 Hz), 109.91 (d, *J* = 4.5 Hz), 109.76 (d, *J* = 4.4 Hz), 109.07, 99.86 (t, *J* = 19.8 Hz), 95.52, 92.43 (t, *J* = 19.2 Hz), 80.47, 71.44, 71.36, 29.49, 28.45, 22.64, 14.15.


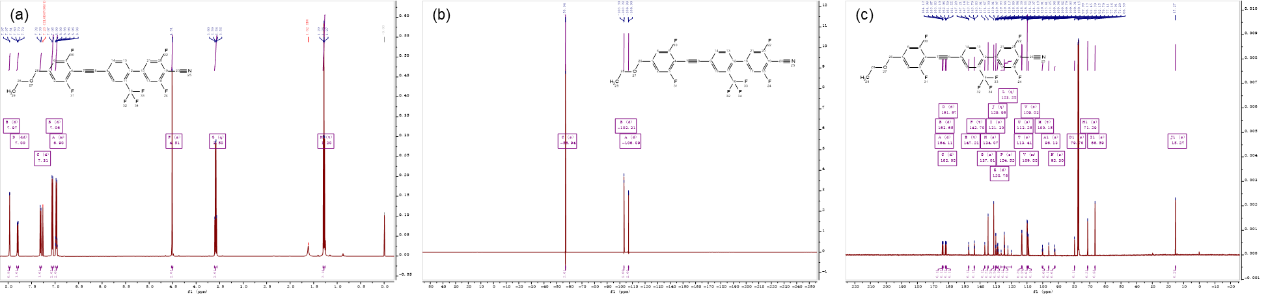


**Scheme S72**. (a) ^1^H, (b) ^19^F and (c) ^13^C NMR spectra of SCUT22e.

**SCUT22e**: ^1^H NMR (500 MHz, Chloroform-*d*) δ 7.97 (d, *J* = 1.6 Hz, 1H), 7.80 (dd, *J* = 8.0, 1.7 Hz, 1H), 7.31 (d, *J* = 8.0 Hz, 1H), 7.06 (d, *J* = 8.0 Hz, 2H), 7.00 – 6.95 (m, 2H), 4.51 (s, 2H), 3.58 (q, *J* = 7.0 Hz, 2H), 1.28 (t, *J* = 7.0 Hz, 3H). ^19^F NMR (471 MHz, Chloroform-*d*) δ -56.94, -103.31 (d, *J* = 8.4 Hz), -106.89 (d, *J* = 8.9 Hz). ^13^C NMR (126 MHz, Chloroform-*d*) δ 164.11 (d, *J* = 5.5 Hz), 163.65 (d, *J* = 5.0 Hz), 162.08 (d, *J* = 5.4 Hz), 161.57 (d, *J* = 5.0 Hz), 147.21 (t, *J* = 10.0 Hz), 143.70 (t, *J* = 8.8 Hz), 137.01, 134.87, 131.30, 129.95 (q, *J* = 5.4 Hz), 128.76 (d, *J* = 31.2 Hz), 124.52, 123.28 (q, *J* = 274.2 Hz), 113.41, 113.25, 110.06 – 109.71 (m), 109.03, 100.15 (t, *J* = 19.9 Hz), 96.13, 92.30, 79.70, 71.29, 66.59, 15.27.


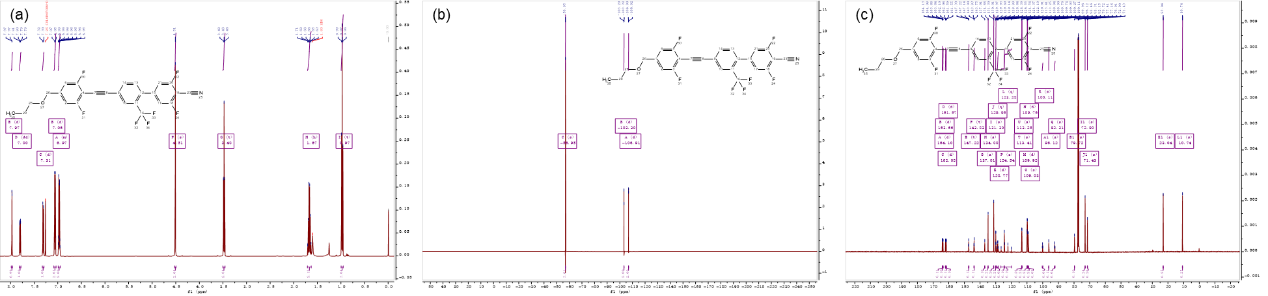


**Scheme S73**. (a) ^1^H, (b) ^19^F and (c) ^13^C NMR spectra of SCUT22f.

**SCUT22f**: ^1^H NMR (500 MHz, Chloroform-*d*) δ 7.97 (d, *J* = 1.6 Hz, 1H), 7.80 (dd, *J* = 7.9, 1.6 Hz, 1H), 7.31 (d, *J* = 7.9 Hz, 1H), 7.06 (d, *J* = 7.9 Hz, 2H), 6.99 – 6.95 (m, 2H), 4.51 (s, 2H), 3.48 (t, *J* = 6.6 Hz, 2H), 1.67 (h, *J* = 7.2 Hz, 2H), 0.97 (t, *J* = 7.4 Hz, 3H). ^19^F NMR (471 MHz, Chloroform-*d*) δ -56.95, -103.30 (d, *J* = 11.0 Hz), -106.91 (d, *J* = 8.0 Hz). ^13^C NMR (126 MHz, Chloroform-*d*) δ 164.10 (d, *J* = 5.5 Hz), 163.66 (d, *J* = 5.2 Hz), 162.08 (d, *J* = 5.6 Hz), 161.57 (d, *J* = 5.0 Hz), 147.22 (t, *J* = 10.0 Hz), 143.83 (t, *J* = 8.9 Hz), 137.01, 134.88, 131.30, 129.95 (q, *J* = 5.4 Hz), 128.77 (d, *J* = 31.3 Hz), 124.54, 123.28 (q, *J* = 274.1 Hz), 113.41, 113.25, 109.92 (d, *J* = 4.6 Hz), 109.76 (d, *J* = 4.4 Hz), 109.03, 100.11, 96.12, 92.31, 79.72, 72.90, 71.43, 23.04, 10.74.

**Scheme S74**. (a) ^1^H, (b) ^19^F and (c) ^13^C NMR spectra of SCUT23a.

**SCUT23a**: ^1^H NMR (500 MHz, Chloroform-*d*) δ 7.26 – 7.23 (m, 2H), 7.12 (dt, *J* = 8.0, 1.3 Hz, 2H), 7.05 – 7.00 (m, 2H), 4.52 (s, 2H), 3.49 (t, *J* = 6.6 Hz, 2H), 1.68 (dtd, *J* = 14.0, 7.4, 6.6 Hz, 2H), 0.98 (t, *J* = 7.4 Hz, 3H). ^19^F NMR (471 MHz, Chloroform-*d*) δ -103.30 (d, *J* = 8.4 Hz), -106.63, -113.77 (d, *J* = 11.0 Hz). ^13^C NMR (126 MHz, Chloroform-*d*) δ 164.07 (d, *J* = 5.5 Hz), 163.72 (d, *J* = 5.9 Hz), 161.99 (d, *J* = 5.2 Hz), 161.69 (d, *J* = 5.6 Hz), 160.73 (d, *J* = 6.6 Hz), 158.73 (d, *J* = 6.7 Hz), 143.44 (t, *J* = 9.4 Hz), 133.35 (t, *J* = 10.8 Hz), 130.26 (t, *J* = 11.9 Hz), 115.48 (d, *J* = 3.9 Hz), 115.32 (d, *J* = 3.9 Hz), 114.33 (t, *J* = 17.7 Hz), 113.86, 113.68, 110.65 (d, *J* = 5.4 Hz), 110.49 (d, *J* = 5.2 Hz), 108.99, 100.48, 95.62, 93.01, 83.13, 77.41, 77.16, 76.91, 72.89, 71.38, 23.05, 10.75.

**Scheme S75**. (a) ^1^H, (b) ^19^F and (c) ^13^C NMR spectra of SCUT23b.

**SCUT23b**: ^1^H NMR (500 MHz, Chloroform-*d*) δ 7.27 – 7.24 (m, 2H), 7.12 (dt, *J* = 8.0, 1.3 Hz, 2H), 7.04 – 6.99 (m, 2H), 4.51 (s, 2H), 3.53 (t, *J* = 6.6 Hz, 2H), 1.67 – 1.62 (m, 2H), 1.47 – 1.39 (m, 2H), 0.95 (t, *J* = 7.4 Hz, 3H). ^19^F NMR (471 MHz, Chloroform-*d*) δ -103.30 (d, *J* = 8.4 Hz), -106.64 (d, *J* = 8.4 Hz), -113.77 (d, *J* = 8.5 Hz). ^13^C NMR (126 MHz, Chloroform-*d*) δ 164.07 (d, *J* = 5.4 Hz), 163.72 (d, *J* = 5.8 Hz), 161.99 (d, *J* = 5.4 Hz), 161.69 (d, *J* = 5.8 Hz), 160.73 (d, *J* = 6.6 Hz), 158.73 (d, *J* = 6.5 Hz), 143.45 (t, *J* = 9.4 Hz), 133.35 (t, *J* = 10.6 Hz), 130.26 (t, *J* = 11.9 Hz), 115.48 (d, *J* = 3.9 Hz), 115.31 (d, *J* = 3.8 Hz), 114.33 (t, *J* = 17.7 Hz), 113.86, 113.68, 110.65 (d, *J* = 5.4 Hz), 110.48 (d, *J* = 5.0 Hz), 108.99, 100.48, 95.62, 93.01 (t, *J* = 19.4 Hz), 83.13, 71.41, 71.04, 31.89, 19.50, 14.05.

**Scheme S76**. (a) ^1^H, (b) ^19^F and (c) ^13^C NMR spectra of SCUT24a.

**SCUT24a**: ^1^H NMR (500 MHz, Chloroform-*d*) δ 7.25 – 7.21 (m, 2H), 7.19 – 7.14 (m, 2H), 6.98 – 6.93 (m, 2H), 4.50 (s, 2H), 3.47 (t, *J* = 6.6 Hz, 2H), 1.67 (dtd, *J* = 14.0, 7.4, 6.6 Hz, 2H), 0.97 (t, *J* = 7.4 Hz, 3H). ^19^F NMR (471 MHz, Chloroform-*d*) δ -103.18 (d, *J* = 8.4 Hz), -106.08 (d, *J* = 8.2 Hz), -106.68 (d, *J* = 8.3 Hz). ^13^C NMR (126 MHz, Chloroform-*d*) δ 164.09, 164.05, 163.76 (d, *J* = 6.0 Hz), 162.05 (d, *J* = 5.3 Hz), 161.98 (d, *J* = 5.4 Hz), 161.73 (d, *J* = 6.0 Hz), 144.20 (t, *J* = 8.8 Hz), 130.06 (t, *J* = 12.0 Hz), 126.21 (t, *J* = 11.9 Hz), 115.46 (d, *J* = 3.8 Hz), 115.29 (d, *J* = 3.7 Hz), 114.93 (d, *J* = 5.2 Hz), 114.77 (d, *J* = 5.0 Hz), 109.90 (d, *J* = 4.3 Hz), 109.74 (d, *J* = 4.2 Hz), 108.94, 101.65 (t, *J* = 19.8 Hz), 99.84, 96.57, 95.84, 93.13 (t, *J* = 19.3 Hz), 82.90, 81.40, 72.91, 71.39, 23.03, 10.73.

**Scheme S77**. (a) ^1^H, (b) ^19^F and (c) ^13^C NMR spectra of SCUT24b.

**SCUT24b**: ^1^H NMR (500 MHz, Chloroform-*d*) δ 7.25 – 7.22 (m, 2H), 7.19 – 7.14 (m, 2H), 6.98 – 6.93 (m, 2H), 4.49 (t, *J* = 0.8 Hz, 2H), 3.51 (t, *J* = 6.6 Hz, 2H), 1.66 – 1.61 (m, 2H), 1.46 – 1.38 (m, 2H), 0.94 (t, *J* = 7.4 Hz, 3H). ^19^F NMR (471 MHz, Chloroform-*d*) δ -103.18 (d, *J* = 8.0 Hz), -106.08 (d, *J* = 8.3 Hz), -106.68 (d, *J* = 8.5 Hz). ^13^C NMR (126 MHz, Chloroform-*d*) δ 164.09, 164.05, 163.76 (d, *J* = 6.0 Hz), 162.05 (d, *J* = 5.5 Hz), 161.98 (d, *J* = 5.2 Hz), 161.73 (d, *J* = 6.0 Hz), 144.21 (t, *J* = 9.0 Hz), 130.06 (t, *J* = 12.0 Hz), 126.21 (t, *J* = 11.9 Hz), 115.46 (d, *J* = 3.8 Hz), 115.29 (d, *J* = 3.7 Hz), 114.93 (d, *J* = 5.2 Hz), 114.77 (d, *J* = 5.0 Hz), 109.90 (d, *J* = 4.8 Hz), 109.74 (d, *J* = 4.3 Hz), 108.94, 101.65, 99.84, 96.57, 95.84, 82.90, 81.41, 71.43, 71.05, 31.87, 19.48, 14.03.

**Scheme S78**. (a) ^1^H, (b) ^19^F and (c) ^13^C NMR spectra of SCUT25a.

**SCUT25a**: ^1^H NMR (500 MHz, Chloroform-*d*) δ 7.23 – 7.18 (m, 2H), 7.03 – 6.99 (m, 2H), 6.98 – 6.94 (m, 2H), 4.50 (s, 2H), 3.51 (t, *J* = 6.5 Hz, 2H), 1.66 – 1.60 (m, 2H), 1.46 – 1.38 (m, 2H), 0.94 (t, *J* = 7.4 Hz, 3H). ^19^F NMR (471 MHz, Chloroform-*d*) δ -61.80 (d, *J* = 53.3 Hz), -101.16 (d, *J* = 9.1 Hz), -106.55 (d, *J* = 8.3 Hz), -110.12 (td, *J* = 27.0, 26.2, 11.2 Hz). ^13^C NMR (126 MHz, Chloroform-*d*) δ 164.81 (d, *J* = 6.6 Hz), 164.15 (d, *J* = 5.4 Hz), 162.73 (d, *J* = 6.7 Hz), 162.11 (d, *J* = 5.4 Hz), 160.92 (d, *J* = 6.0 Hz), 158.86 (d, *J* = 5.2 Hz), 155.21 (t, *J* = 13.6 Hz), 144.50 (t, *J* = 8.9 Hz), 128.85 (t, *J* = 12.8 Hz), 120.29 (t, *J* = 269.7 Hz), 116.10 (d, *J* = 4.0 Hz), 115.91 (d, *J* = 3.2 Hz), 109.92 (d, *J* = 4.3 Hz), 109.76 (d, *J* = 4.4 Hz), 109.65 – 109.44 (m), 108.69, 106.06 (d, *J* = 3.8 Hz), 105.87 (d, *J* = 3.9 Hz), 99.59 (t, *J* = 19.7 Hz), 94.78, 90.20 (t, *J* = 19.4 Hz), 81.64, 71.42, 71.08, 31.87, 19.48, 14.03.

**Reference**

[1] Y. Song, J. Li, R. Xia, H. Xu, X. Zhang, H. Lei, W. Peng, S. Dai, S. Aya, M. Huang, Phys. Chem. Chem. Phys. 2022, 24, 11536, <https://doi.org/10.1039/d2cp01110g>.

[2] Y. Song, M. Deng, Z. Wang, J. Li, H. Lei, Z. Wan, R. Xia, S. Aya, M. Huang, J. Phys. Chem. Lett. 2022, 9983, <https://doi.org/10.1021/acs.jpclett.2c02846>.
